# iTRAQ-based quantitative tissue proteomic analysis of differentially expressed proteins (DEPs) in non-transgenic and transgenic soybean seeds

**DOI:** 10.1038/s41598-018-35996-y

**Published:** 2018-12-05

**Authors:** Weixiao Liu, Wentao Xu, Liang Li, Mei Dong, Yusong Wan, Xiaoyun He, Kunlun Huang, Wujun Jin

**Affiliations:** 1 Biotechnology Research Institute, Chinese Agricultural and Academic Sciences, Beijing, 100081 PR China; 2 Laboratory of Food Safety and Molecular Biology, College of Food Science and Nutritional Engineering, China Agricultural University, Beijing, 100083 PR China

## Abstract

The unintended effects of transgenesis have increased food safety concerns, meriting comprehensive evaluation. Proteomic profiling provides an approach to directly assess the unintended effects. Herein, the isobaric tags for relative and absolute quantitation (iTRAQ) comparative proteomic approach was employed to evaluate proteomic profile differences in seed cotyledons from 4 genetically modified (GM) and 3 natural genotypic soybean lines. Compared with their non-GM parents, there were 67, 61, 13 and 22 differentially expressed proteins (DEPs) in MON87705, MON87701 × MON89788, MON87708, and FG72. Overall, 170 DEPs were identified in the 3 GM soybean lines with the same parents, but 232 DEPs were identified in the 3 natural soybean lines. Thus, the differences in protein expression among the genotypic varieties were greater than those caused by GM. When considering ≥2 replicates, 4 common DEPs (cDEPs) were identified in the 3 different GM soybean lines with the same parents and 6 cDEPs were identified in the 3 natural varieties. However, when considering 3 replicates, no cDEPs were identified. Regardless of whether ≥2 or 3 replicates were considered, no cDEPs were identified among the 4 GM soybean lines. Therefore, no feedback due to GM was observed at the common protein level in this study.

## Introduction

Genetically modified (GM) crop production has increased dramatically over the past 20 years, growing more than 100-fold from 1.7 million hectares to 180 million hectares globally. Of these crops, soybean (*Glycine max*) is an economically important crop, with the planting area of transgenic soybeans now occupying more than 50% of the area covered by all transgenic crops. GM crops are modified by the insertion of exogenous DNA fragments to synthesize new substances that improve the nutrient components of the crops (such as *fatb1-A* and *fad2-1A* in MON87705) and/or enhance the tolerance of the crops to herbicides (such as *cp4 epsps* in MON87705, *dmo* and *cp4 epsps* in MON87708, *cp4 epsps* in MON87701 × MON89788, and *2mepsps* and *hppdPF W336* in FG72) and insects (such as *cry1Ac* in MON87701 × MON89788)^1^. The rapid development of GM crop production has resulted in considerable economic benefits; however, consumer concern that GM products may lead to unforeseen food and environmental safety issues has increased^2–4^. Remaining questions include the possibility that the insertion of exogenous DNA fragments into the genomes of crops might lead to the deletion, insertion or rearrangement of some genes, affecting biochemical pathways or resulting in the formation of new biological products (such as new allergens or toxins)^5,6^. Therefore, the safety of GM crops must be evaluated^7,8^, and substantial equivalence is the cornerstone of these safety assessments. Through the development of new methodologies, indicators of substantial equivalence have become increasingly abundant. Innovative profiling techniques (such as genomics, transcriptomics, proteomics, and metabolomics) enable comprehensive measurements and comparisons of the transcripts, proteins and metabolites of organisms and provide detailed insights into any unintended changes in the GM crops being studied^9–13^. Therefore, in this study, we applied profiling techniques to study the substantial equivalence of GM crops.

Proteins are important components of living organisms, not only for their role in gene function but also for their roles as toxins or allergens^14,15^. Proteomics has developed continuously over the past two decades, providing substantial contributions to the field of omics, and is now a widely accepted and reproducible method for the study of various species^16–20^. For many years, two-dimensional electrophoresis (2-DE) combined with mass spectrometry (MS) has been the most widely used technique in plant proteomics. However, 2-DE has certain distinct disadvantages, including the ability to identify only a limited number of proteins within a sample and insensitivity to small and low-abundance proteins^4,5,10,20^. In contrast, isobaric tags for relative and absolute quantitation (iTRAQ) is a high-throughput method with high accuracy, sensitivity and repeatability. The application of iTRAQ in quantitative proteomics has gained widespread popularity, with several studies reporting the efficiency of this method for the examination of differentially expressed proteins (DEPs) in soybean^21–24^. However, proteomic data for soybean seed cotyledons are currently unavailable, and comparative proteomic research on transgenic and non-transgenic soybeans is also lacking. The challenge in transgenic omics research lies in the selection and preparation of samples. Most of the current research involves comparative single-species studies of transgenic lines and their non-GM parents, with few or no investigations into different transgenic variations in the same parents or simultaneous comparative studies of multiple transgenic lines and their parents. The aim of this study was, therefore, to fill these gaps by both exploring and enriching the field of GM and non-GM soybean proteomics.

In this study, iTRAQ was applied to the proteomic analysis of soybean seed cotyledons from seven different soybean lines (4 GM lines, namely, MON87701 × MON89788, MON87708, MON87705 and FG72, and 3 natural genotypic soybean lines, namely, Zhonghuang13, A3525 and FG72-JACK) to expand the depth and breadth of our knowledge of GM and non-GM soybean protein expression. The five primary aims of the study were: (1) to perform soybean seed cotyledon protein profiling; (2) to compare the obtained seed cotyledon protein expression patterns of natural genotypic soybean lines; (3) to compare DEPs between GM soybeans and their parents; (4) to identify common DEPs (cDEPs) among different GM soybean lines; and (5) to determine whether differences in seed protein expression among natural genotypic soybean lines were more notable than those caused by transgenic modification.

## Results

**Figure 1:**
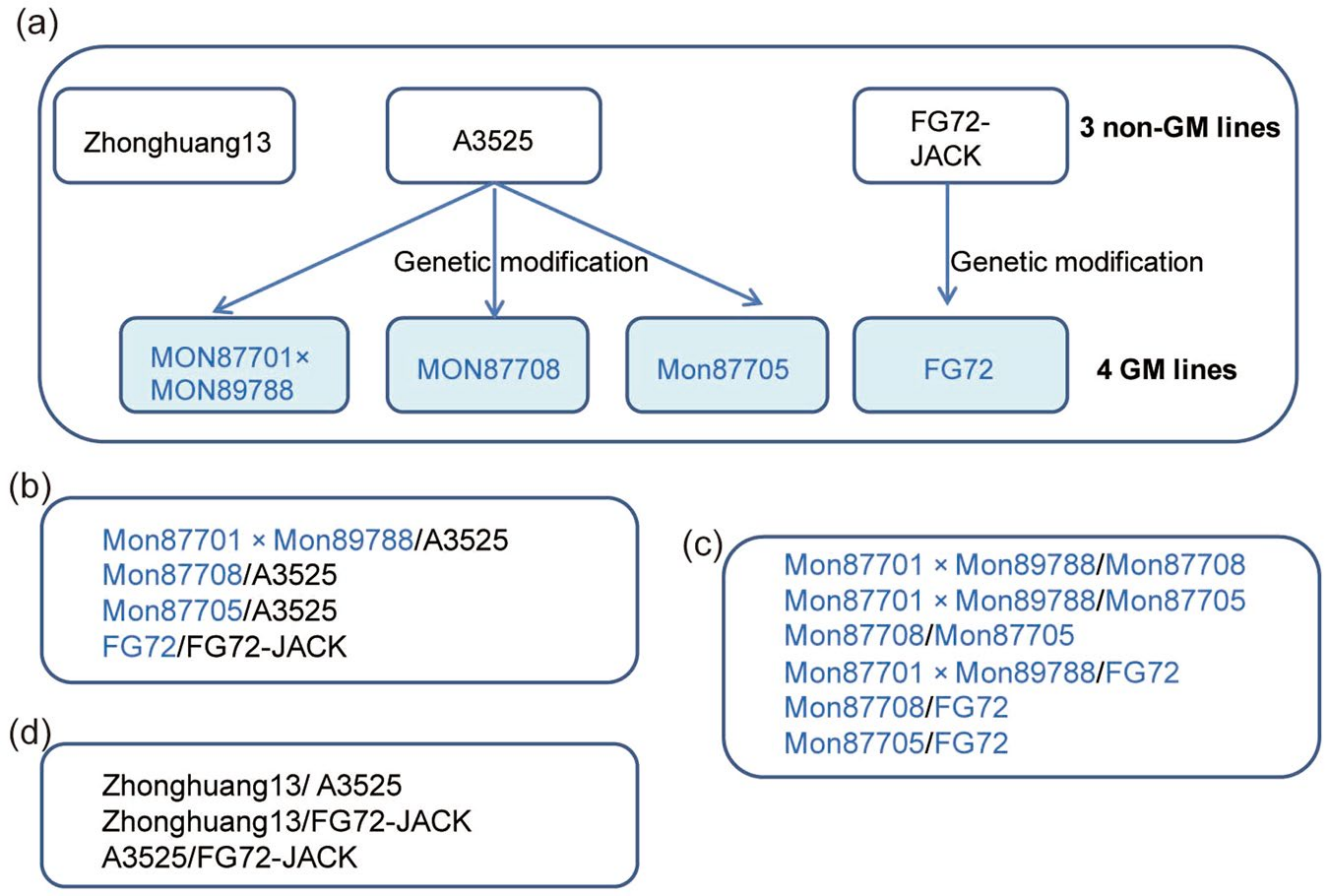
The genetic relations among the studied soybean lines and grouping comparison design. (**a**) The genetic relations among the studied soybean lines. (**b**) Grouping comparison study between GM soybean lines and their non-GM parents. (**c**) Grouping comparison study among GM soybean lines. (**d**) Grouping comparison study among natural genotypic soybean lines.

In this study, soybean seed cotyledons were used to study proteomic differences among GM and natural genotypic soybean varieties. Compared with other tissues, soybean seed cotyledons have large cells with abundant contents, including a wide variety of protein types. Furthermore, as storage tissue, soybean seed cotyledons exhibit stable protein expression and are the most suitable tissue for comparative proteomic research. The genetic relations among the studied soybean lines and the design of the proteomic data analysis are shown in Table 1 and Fig. 1(a). DEPs and cDEPs were selected from three grouping comparison studies, including comparisons between GM soybean lines and their non-GM parents, among GM soybean lines and among natural genotypic soybean lines (Fig. 1(b–d)). The event-specific PCR method, which is the most precise approach for transgenic crop detection^25,26^, was used to detect specific events in transgenic soybean lines. The target DNA fragment was obtained from 4 GM soybean lines (Supplementary Fig. S1). The obtained DNA products were further verified by DNA sequencing.

**Table 1:** Summary of the studied soybean lines.

Natural genotypic lines	GM soybean lines	Foreign genes		
A3525	MON87705^48^	*cp4 epsps*	*fatb1-A*	*fad2-1A*
	MON87708^49^	*cp4 epsps*	*dmo*	
	MON87701 × MON89788^50^	*cp4 epsps*	*cry1Ac*	
FG72-JACK	FG-72^51^	*2mepsps*	*hppdPF W336*	
Zhonghuang 13	no	no

### Repeatability of soybean seed cotyledon protein profiling

**Figure 2:**
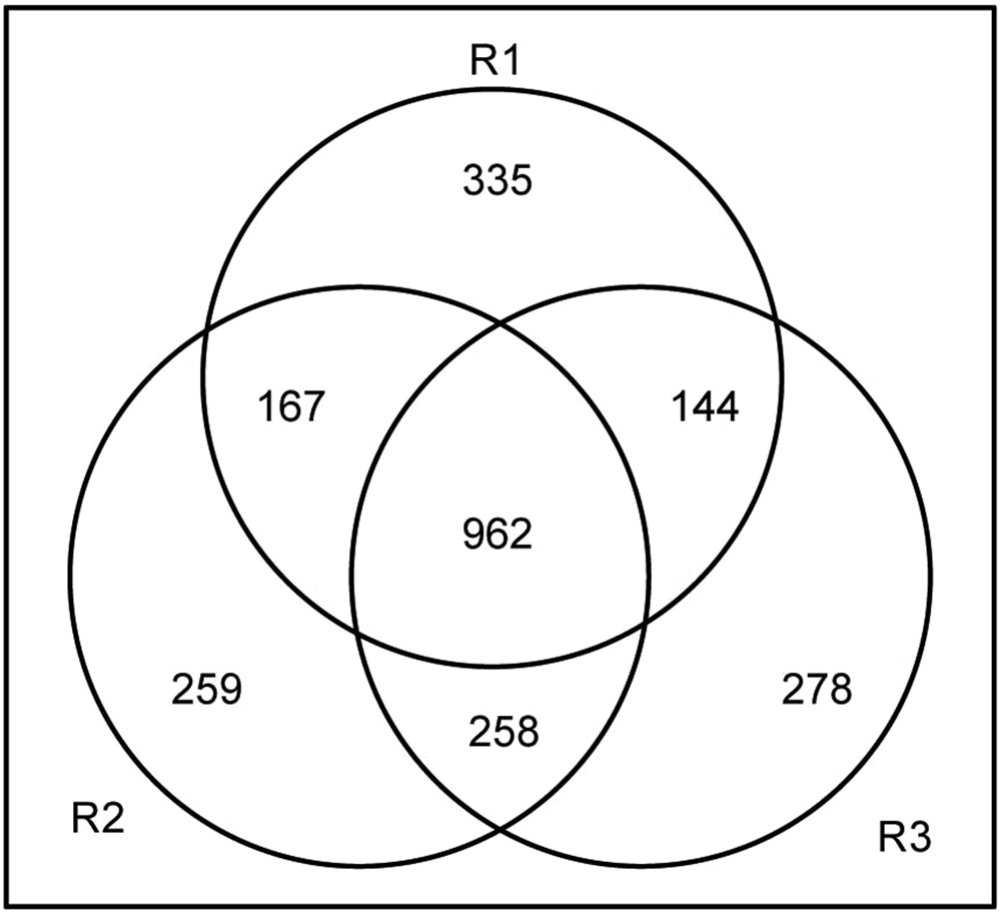
Proteins identified in the three biological replicates. R1, replicate 1, 1608 unique proteins identified. R2, replicate 2, 1646 unique proteins identified. R3, replicate 3, 1642 unique proteins identified.

A proteomic analysis was used to identify soybean seed cotyledon proteins and the differences in the abundances of the proteins among various transgenically modified soybean lines. Three replicate iTRAQ experiments using soybean seed cotyledons from seven different lines as experimental materials generated 41738, 43013 and 39666 spectra. From these iTRAQ replicates, 7591, 7696 and 7558 peptides were identified, of which 4290, 4466 and 4477 were unique peptides. Moreover, 1608, 1646 and 1642 proteins were also identified from the iTRAQ replicates (Table 2), with a total of 2403 proteins, which are listed in Supplementary Table S1. A cluster analysis of each iTRAQ experiment showed that the data obtained for each of the three replicates were reproducible (Supplementary Fig. S2). Among the identified proteins, 962 were identified in all three replicates, and 569 were identified in two replicates (Fig. 2). These overlapping proteins were considered reproducibly identified proteins and were thus selected for the quantitative comparative analysis.

**Table 2:** Summary of the protein identification data.

Database	No.	Spectra (PSM)	Peptides	Unique peptides	Protein groups
*Glycine max*	1st	41738	7591	4290	1608
*Glycine max*	2nd	43013	7696	4466	1646
*Glycine max*	3rd	39666	7558	4477	1642

PSM, peptide spectrum match.

### Identification and analysis of DEPs caused by single gene insertions

To identify the proteins that were differentially expressed due to single gene insertions, the iTRAQ ratios (1531 reproducibly identified proteins) of MON87701 × MON89788/A3525 (115/117), MON87708/A3525 (118/117), MON87705/A3525 (114/117) and FG72/FG72-JACK (116/119) were investigated. Proteins with changes in abundance greater than 1.5-fold (p-value < 0.05) were designated as significantly upregulated. Similarly, proteins with changes in abundance less than 0.67-fold (p-value < 0.05) were designated as significantly downregulated.

**Figure 3:**
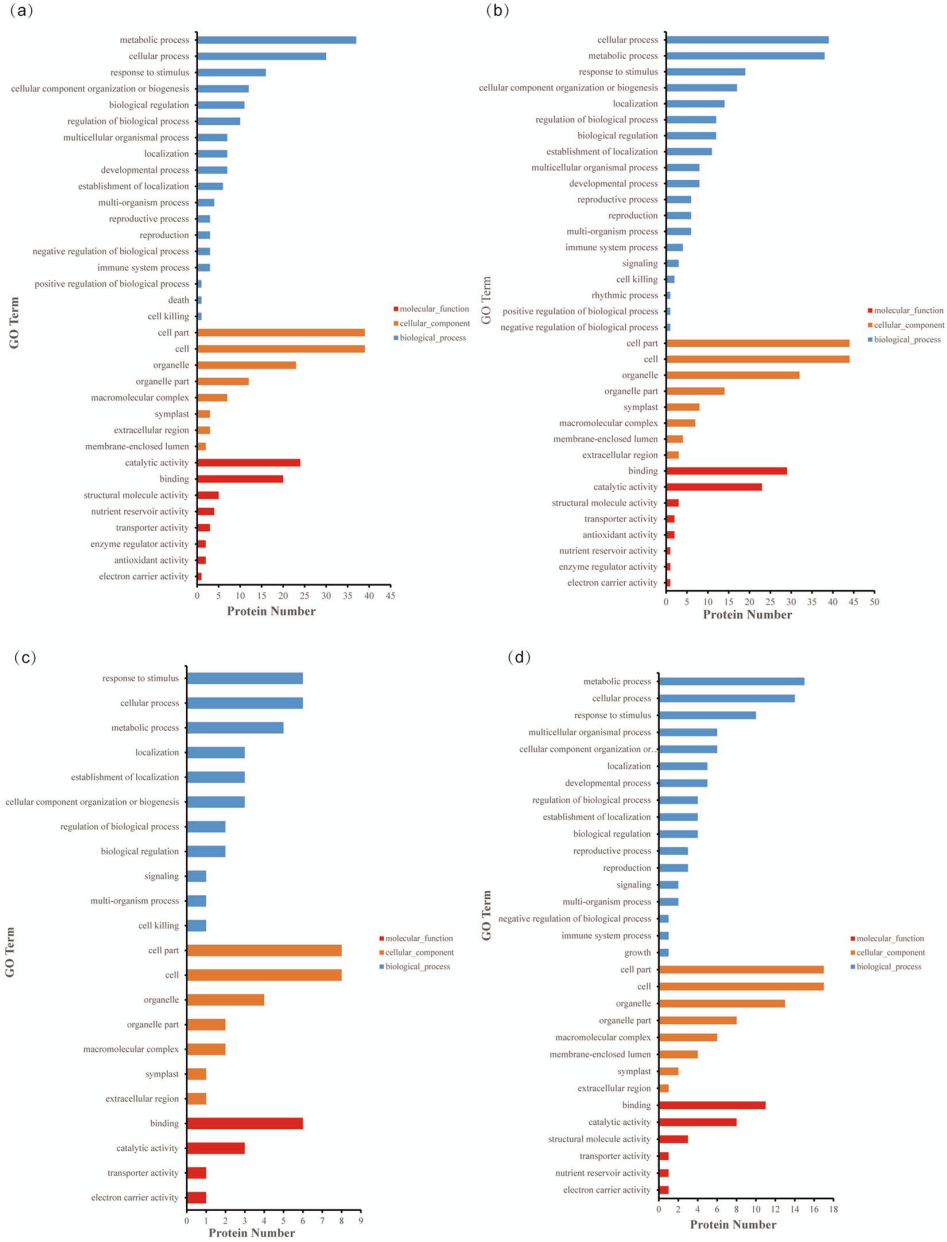
GO annotations of the identified DEPs. DEPs were annotated into 3 main categories, biological process, cellular component and molecular function, to determine the functions of the identified DEPs between the GM soybean lines and their parents (**a**) MON87701 × MON89788/A3525; (**b**) MON87705/A3525; (**c**) MON87708/A3525; and (**d**) FG72/FG72-JACK.

The specific findings are listed below. In the MON87701 × MON89788/A3525 samples, 42 proteins were upregulated and 19 proteins were downregulated, while 61 proteins were differentially expressed. The results of the GO (Gene Ontology) annotation analysis revealed 61 DEPs involved in 34 functional groups, including 18 biological processes, 8 cellular components and 8 molecular functions (as shown in Fig. 3(a) and Supplementary Table S2). In the MON87705/A3525 samples, 67 proteins were differentially expressed, with 52 upregulated proteins and 15 downregulated proteins. All 67 DEPs were annotated into 35 functional groups, including 19 biological processes, 8 cellular components and 8 molecular functions (Fig. 3(b) and Supplementary Table S3). However, in the MON87708/A3525 samples, only 8 proteins were upregulated, and 5 proteins were downregulated. The results of the GO annotation analysis showed that the 13 DEPs were involved in 22 functional groups, including 11 biological processes, 7 cellular components and 4 molecular functions (Fig. 3(c) and Supplementary Table S4). Twenty-two proteins were differentially expressed, with half upregulated and half downregulated, in the FG72/FG72-JACK samples. All 22 DEPs were annotated into 31 functional groups, including 19 biological processes, 8 cellular components and 8 molecular functions (Fig. 3(d) and Supplementary Table S5). The four groups of DEPs were mainly involved in metabolism, cellular processes and responses to stimuli in the biological processes; the cell, cell parts and organelles in the cellular components; and catalytic activity and binding in the molecular functions.

The four GM soybean lines studied were transformed with genes that encoded 3-phosphoshikimate 1-carboxyvinyltransferase (EPSPS, also named 5-enolpyruvylshikimate-3-phosphate synthase), a glyphosate-insensitive enzyme, and were therefore glyphosate tolerant. EPSPS was differentially expressed in the GM soybean lines MON87705 and MON87701 × MON89788 in 3 iTRAQ replicates (Table 3).

**Table 3:** EPSPS expression in different soybean lines.

Soybean line	EPSPS expression fold change			Differential expression
	1st	2nd	3rd	
MON87705/A3525	3.816	3.068	3.344	Upregulated
MON87701 × MON89788/A3525	2.410	2.299	2.398	Upregulated

To screen for cDEPs resulting from transgenic modification, the identified DEPs were classified into 3 replicates and 2 replicates of identified proteins. In three different GM soybean lines (namely, MON87701 × MON89788, MON87708 and MON87705) with the same non-GM parent (A3525), 4 cDEPs, with 3 proteins upregulated and 1 protein downregulated, were identified in ≥2 replicates, but no common DEPs were observed in the 3 replicates. However, no cDEP was identified in four different GM soybean lines (namely, MON87701 × MON89788, MON87708, MON87705 and FG72) with different non-GM parents in either ≥2 replicates or 3 replicates (Table 4). Based on these results, the insertion of a single gene did not lead to significant changes in either pathways or genes.

**Table 4:** cDEPs in different GM soybean lines*.

cDEPs in 115/117, 114/117 and 118/117					
3 Replicates	≥2 Replicates**				
ID	ID	Description	Gene name	Calc. pI	MW [kDa]
—	I1M0N4	Uncharacterized protein	GLYMA_13G192900	6	124.1
—	I1MTN1	Uncharacterized protein	GLYMA_17G094800	5.62	107.3
—	A0A0R0F0E0	Uncharacterized protein	GLYMA_18G153900	8.31	17.4
—	I1M395	Uncharacterized protein	GLYMA_13G278000	7.84	8.3
**cDEPs in 115/117**, **114/117**, **118/117 and 116/119**					
**3 Replicates**	≥2 Replicates**				
**ID**	**ID**	**Description**	**Gene name**	**Calc**. **pI**	**MW [kDa]**
**—**	**—**				

_—_ No cDEPs.

*115/117: MON87701 × MON89788/A3525;

114/117: MON87705/A3525;

118/117: MON87708/A3525;

116/119: FG72/FG72-JACK.

**Considering 2&3 replicates.

### No common genetically modified markers resulted from gene insertions

By comparing the proteomic profiles of MON87701 × MON89788/MON87705 (115/114), MON87701 × MON89788/MON87708 (115/118), MON87705/MON87708 (114/118), MON87701 × MON89788/FG72 (115/116), MON87705/FG72 (114/116), and MON87708/FG72 (118/116) samples, the DEPs in four GM soybean lines were identified. Thresholds of a greater than 1.5-fold difference in abundance and p-value < 0.05 were used to select the DEPs. In the MON87701 × MON89788/MON87705 samples, 52 proteins were differentially expressed, 11 of which were present in 3 replicates, and 41 were present in 2 replicates. In the MON87701 × MON89788/MON87708 samples, 69 proteins were differentially expressed, with 19 proteins in 3 replicates and 50 proteins in 2 replicates. Furthermore, 49 proteins were differentially expressed in the MON87705/MON87708 samples, with 12 proteins in 3 replicates and 37 proteins in 2 replicates (Supplementary Tables S6-S8). Comparative analyses of these DEPs revealed 2 cDEPs in ≥2 replicates, but no cDEPs were present in 3 replicates of the three different GM soybean lines (MON87701 × MON89788, MON87708 and MON87705) with the same non-GM parent (Table 5).

**Table 5:** cDEPs in different GM soybean lines*.

cDEPs in 115/114, 115/118 and 114/118					
3 Replicates	≥2 Replicates**	Description	Gene name	Calc. pI	MW [kDa]
ID	ID				
—	C6TAQ9	Uncharacterized protein	GLYMA_15G156700	6.55	28.5
—	K7MZ42	Uncharacterized protein	GLYMA_19G186900	6.05	176.0
**cDEPs in 115/114**, **115/118**, **114/118**, **115/116**, **114/116 and 118/116**					
**3 Replicates**	≥2 Replicates**	**Description**	**Gene name**	**Calc**. **pI**	**MW [kDa]**
**ID**	**ID**				
**—**	**—**				

_—_ No cDEPs.

*115/114: MON87701 × MON89788/MON87705;

115/118: MON87701 × MON89788/MON87708;

114/118: MON87705/MON87708;

115/116: MON87701 × MON89788/FG72;

114/116: MON87705/FG72;

118/116: MON87708/FG72.

**Considering 2&3 replicates.

In the MON87701 × MON89788/FG72 samples, 89 proteins were differentially expressed, with 33 proteins in 3 replicates and 56 proteins in 2 replicates, while in the MON87705/FG72 samples, 80 proteins were differentially expressed, with 35 proteins in 3 replicates and 45 proteins in 2 replicates. In the MON87708/FG72 samples, 62 proteins were differentially expressed, with 23 proteins in 3 replicates and 39 proteins in 2 replicates (Supplementary Tables S9-S11). However, no cDEPs were identified in these four different GM soybean lines (MON87701 × MON89788, MON87708, MON87705 and FG72) (Table 5).

**Figure 4:**
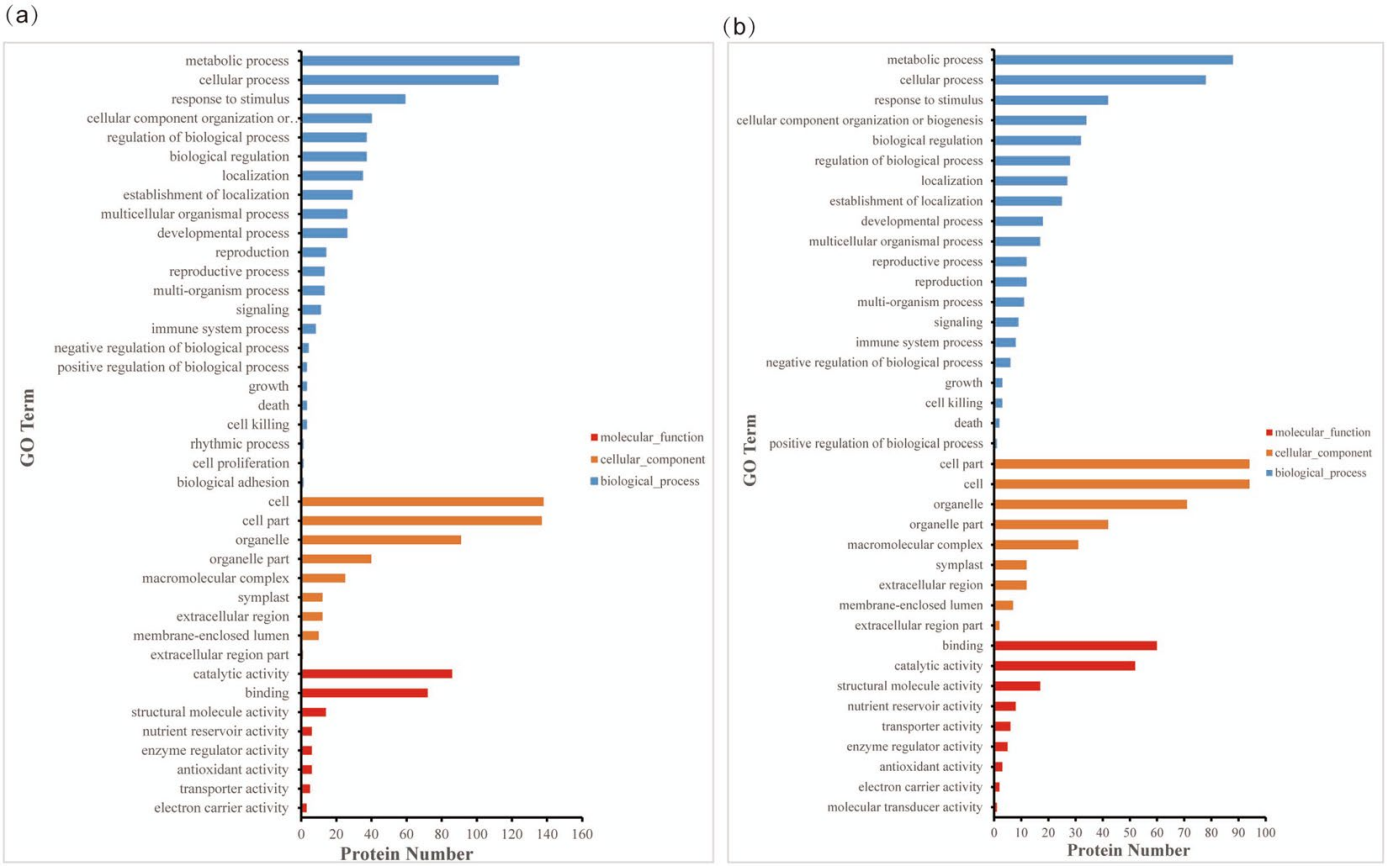
GO annotations of the identified DEPs in the four different GM soybean lines (**a**) and in the three different natural genotypic soybean lines (**b**).

In total, 401 DEPs were annotated into 40 functional groups, including 23 biological processes, 9 cellular components, and 8 molecular functions. Among biological processes, all DEPs were primarily involved in metabolism, cellular process and response to stimulus. Among cellular components, all DEPs were mainly focused on the cell, cell part and organelle. Among molecular functions, all DEPs were mainly involved in catalytic activity and binding (Fig. 4(a)).

The proteomic data also showed that the number of DEPs (231) identified among the GM soybean lines with different non-GM parents (MON87701 × MON89788/FG72, MON87705/FG72, and MON87708/FG72) was greater than the number identified (170) among the lines with the same non-GM parents (MON87701 × MON89788/MON87705, MON87701 × MON89788/MON87708, and MON87705/MON87708).

### Notable differences in protein expression were observed in natural genotypic soybean lines

By comparing the proteomic profiles of samples A3525/FG72-JACK (117/119), A3525/Zhonghuang13 (117/113), and FG72-JACK/Zhonghuang13 (119/113), DEPs in these natural genotypic soybean lines were identified. Thresholds of a 1.5-fold change in abundance and p-value < 0.05 were used to define the DEPs. In the A3525/FG72-JACK samples, 65 proteins were defined as differentially expressed, 27 of which were present in 3 replicates and 38 of which were present in 2 replicates. In the A3525/Zhonghuang13 samples, 64 proteins were defined as differentially expressed, and 26 of these proteins were present in 3 replicates and 38 in 2 replicates, while in the FG72-JACK/Zhonghuang13 samples, 103 proteins were defined as differentially expressed, with 44 proteins in 3 replicates and 59 proteins in 2 replicates (Supplementary Tables S12–S14).

In total, 232 DEPs were annotated into 37 functional groups, including 19 biological processes, 9 cellular components and 8 molecular functions. Among biological processes, all DEPs were primarily involved in metabolism, cellular process and response to stimulus. Among the cellular components, all DEPs were mainly associated with cell part, cell and organelle, while among the molecular functions, all DEPs were primarily involved in binding and catalytic activity (Fig. 4(b)).

Of these DEPs, 6 cDEPs were identified in ≥2 replicates, but no cDEPs were identified in 3 replicates of the different natural genotypic soybean lines Zhonghuang13, A3525 and FG2-JACK (Table 6).

**Table 6:** cDEPs in different natural genotypic soybean lines*.

cDEPs in 117/119, 117/113 and 119/113					
3 Replicates	≥2 Replicates**	Description	Gene name	Calc. pI	MW [kDa]
ID	ID				
—	A0A0B2QKF8	Glucose and ribitol dehydrogenase	glysoja_023775	7.08	31.7
—	Q70EM0	Dehydrin	lea-D-11	6.47	23.8
—	I1KQW4	Uncharacterized protein		6.10	75.4
—	A0A0B2PMR9	Em-like protein GEA6	glysoja_045231	5.60	11.5
—	A0A0R0IIZ3	Uncharacterized protein	GLYMA_09G17810	9.19	57.8
—	C6SX26	Putative uncharacterized protein		5.85	12.1

— No cDEPs.

*117/119: A3525/FG72-JACK;

117/113: A3525/Zhonghuang13;

119/113: FG72-JACK/Zhonghuang13.

**Considering 2&3 replicates.

## Discussion

To understand whether transgenic modification has unintended effects in GM crops, soybean seed cotyledons were assessed via iTRAQ protein profiling to facilitate the study of alterations in protein expression regulated by transgenic modification and/or different natural genotypic soybean lines. The experimental design included three grouping comparisons: GM soybean lines and their non-GM parents, different GM soybean lines, and different natural genotypic soybean lines. The soybean lines and soybean seed cotyledons used in this study were carefully selected. The GM soybean lines, namely, MON87701 × MON89788, MON87705 and MON87708, which have the same parent, namely, A3525, were selected to study the effects of different genes on plants from the same genetic background. Four GM soybean lines were used to study the effects of genetic modifications on plants from different genetic backgrounds, while three natural genotypic soybean lines were studied to understand the differences among different genetic backgrounds. Soybean seed cotyledons are a storage tissue containing numerous protein types and particularly stable expression, making these tissues suitable for comparative proteomic studies.

In 1993, the Organisation for Economic Cooperation and Development (OECD) proposed the principle of “substantial equivalence” for food safety assessments, stating that if a new food or food ingredient is approximately the same as existing foods or food ingredients, they are equally safe. With the development of transgenic crops, the concept of substantial equivalence has been modified by scientists, moving from the original directional substantial equivalence (single component) to non-directional substantial equivalence (overall consideration)^27–29^. Non-directional substantial equivalence evaluated by iTRAQ, was used in the present study to assess potential unintended effects on transgenic soybean seed cotyledons. With an increasing number of similar studies, databases and thresholds for non-directional substantial equivalence analyses might be established to perform the most comprehensive and effective safety evaluation of transgenic crops in the future via the development of innovative methodology.

Proteomics has rapidly developed in the past 20 years and has become a widely accepted method that has been used to study many species. For many years, 2-DE combined with MS was widely used in plant proteomics and identified few to dozens, or more than a hundred proteins^18,20,30,31^. The application of iTRAQ has led to unprecedented developments in proteomics. Not only has the number of identified proteins increased dramatically but quantitative comparisons have also been made possible. More than 1200 proteins have been identified from soybean leaves^21,22^. In this study, 2403 proteins were identified from soybean seed cotyledons. With the development of the proteomic research methods, the numbers of identified proteins are increasing further and quantitative methods are becoming more precise, making the application of this technique to the analysis of non-directional substantial equivalence of an increasing number of GM crops possible.

Compared with their non-GM parents, 61, 67, 13 and 22 DEPs were identified in MON87705, MON87701 × MON89788, MON87708, and FG72, respectively. Compared with the other 2 GM soybean lines, MON87701 × MON89788 and MON87705 are relatively special. MON87701 × MON89788 is a stacked transgenic soybean line. The breeding process itself leads to differential expression of proteins^21^. The superposition effect of transgenetic and breeding processes leads to an increase in the number of DEPs. The *fatb1-A* and *fad2-1A* genes, encoding the acyl-acyl carrier protein thioesterase and delta-12 desaturase to desaturate saturated fatty acids to 18:1 oleic acid and further to 18:2 linoleic acid^32^, were introduced to MON87705 to improve nutrition. Nutritional improvement inevitably affects the metabolic pathway and increases the number of DEPs^33–35^. Thus, much more DEPs were identified in the GM soybean lines MON87701 × MON89788 and MON87705. A total of 170 DEPs were identified in 3 GM soybean lines, namely, MON87701 × MON89788, MON87705, and MON87708, with the same parents, but 232 DEPs were identified in the 3 natural genotypic soybean lines. Therefore, more differences in protein expression were observed among natural genotypic varieties than in transgenic strains.

The shikimate pathway is a metabolic pathway for the biosynthesis of aromatic amino acids in microorganisms and plants. Seven enzymes, DAHP synthase, 3-dehydroquinate synthase, 3-dehydroquinate dehydratase, shikimate dehydrogenase, shikimate kinase, EPSPS and chorismate synthase, are involved in the shikimate pathway^36–38^. EPSPS was differentially expressed in the GM soybean lines MON87705 and MON87701 × MON89788.

Comparisons of the protein profiles of GM soybean lines and their parents revealed 4 cDEPs in ≥ 2 replicates, but no cDEPs in 3 replicates among the three GM soybean lines (namely, MON87701 × MON89788, MON87708 and MON87705) with the same non-GM parent (A3525). However, no cDEPs were identified in the four GM soybean lines (namely, MON87701 × MON89788, MON87708, MON87705 and FG72) with different non-GM parents (A3525 and FG72-JACK), whether in the 2 replicates or in the 3 replicates. Based on these results, gene insertion did not result in a common protein-level response and feedback in soybean seeds.

## Conclusions

In this study, iTRAQ quantitative proteomics was used to evaluate the proteome variations created by both transgenic modifications and differences in natural genotypic lines in soybean seed cotyledons. No common protein marker was identified in the different GM soybean lines, and indeed, the variations in protein expression among the different natural genotypic soybean lines were more notable than variations caused by transgenic modifications. Furthermore, this study revealed the benefits of iTRAQ-based protein profiling of natural genotypic soybean lines.

## Materials and Methods

### Reagents and materials

HPLC-quality water was obtained from a Cascada TM IX water purification system (Pall Co., NY, USA). Methanol (HPLC-grade) was purchased from Thermo Fisher Scientific (MA, USA). Urea and CHAPS (3-[(3-cholamidopropyl)dimethylammonio]−1-propanesulfonate) were purchased from Bio-Rad Laboratories, Inc. (CA, USA). Thiourea, ammonia, formic acid, methyl alcohol and bovine serum albumin were purchased from Sigma-Aldrich Corporation (MO, USA). Trypsin, reducing reagent, cysteine-blocking reagent and the dissolution buffer in the iTRAQ Kit and iTRAQ 8plex Kit were purchased from AB Sciex Corporation (Washington, D.C., USA). Acetonitrile was purchased from Merck (NJ, USA). The Durashell-C18 column was purchased from Agela (DE, USA). The soybean seeds used in this study represented 4 GM soybean lines, namely, MON87705, MON87701 × MON89788, FG72 and MON87708, and 3 natural genotypic soybean lines, namely, A3525, JACK and Zhonghuang13 (Table 1), and were collected by our labs and stored at −80 °C.

### DNA extraction and event-specific PCR of transgenic soybeans

Genomic DNA was extracted from soybeans using the EasyPure Plant Genomic DNA Kit (Tiangen, Beijing, China) according to the manufacturer’s instructions. The genomic DNA concentrations were quantified by using a NanoDrop 2000 (Thermo Scientific, USA). The DNA was stored at −20 °C until further analysis. Event-specific PCR was performed according to the Chinese National Standards MOA-2122-4-2014^39^, MOA-2259-6-2015^40^, MOA-2259-8-2015^41^, MOA-2259-7-2015^42^ and MOA-1485-6-2010^43^. The sequences of the primers used and the sizes of the amplified DNA fragments are listed in Table S15.

### Protein preparation

Three biological replicates of the seven different lines of soybean seed cotyledons were used for protein profiling in this study. Twenty grains of soybean seed cotyledons were ground in liquid N_2_, and the total proteins were extracted with one millilitre of lysis buffer containing 7 M urea, 2 M thiourea, 0.1% CHAPS and protease inhibitor. After centrifugation at 15,000 × g for 20 min at 4 °C, the supernatant was collected and transferred to a fresh tube. The concentration of the extracted protein was measured by using the Bradford protein assay^44^.

### Trypsin digestion and iTRAQ labelling

The extracted protein solution containing 200 µg of protein was digested with 4 µg of trypsin overnight at 37 °C. Protein reduction, blocking of cysteine residues, and digestion were performed according to the manufacturer’s protocol included with the iTRAQ kit. The digested peptides were transferred to vials containing individual iTRAQ reagents, according to the standard iTRAQ protocol for the 8-plex kit. The tags used were 114 Da for MON87705, 115 Da for MON87701 × MON89788, 116 Da for FG72, 118 Da for MON87708, 113 Da for Zhonghuang13, 117 Da for A3525 and 119 Da for JACK. The labelled samples were pooled in equal amounts, centrifuged under a vacuum, and freeze-dried.

### LC and MS/MS analyses

The peptide mixture was redissolved in solution A (98% ddH_2_O and 2% acetonitrile, pH 10.0) and then fractionated by high pH (10.0) separation using a RIGOL L-3000RP-HPLC system (Beijing Puyuan Power Technology Co., Ltd.), after which 100 μg of the mixture was desalted and fractionated using a Durashell-C18 reverse phase column. Next, solution B (98% acetonitrile and 2% ddH_2_O) was added, and the pH was adjusted to 10.0. After separation, the fractions were resuspended in 20 μL of solution C (0.1% formic acid and 2% methanol in water), separated by using a nano-LC system (Thermo Fisher Scientific Corp., MA, USA) and analysed on-line using electrospray tandem MS.

Nano-LC-MS/MS experiments were performed using an EASY-nLC 1000 coupled with a Q-Exactive system. Peptides were loaded on a nanocolumn (EASY-Spray column, C18) balanced with solvent D (0.1% formic acid acetonitrile solution). The Q-Exactive mass spectrometer was operated in the data-dependent mode to switch automatically between MS and MS/MS acquisitions. Surveys of the full-scan MS spectra (m/z 350-1800) were acquired with a mass resolution of 70000 FWHM, followed by 15 sequential high-energy collisional dissociation MS/MS scans with a resolution of 17500 FWHM.

### Analysis of the proteomic data and bioinformatics

The original files generated by the Q-Exactive system were analysed using Proteome Discoverer 1.4 software (Fisher Scientific Thermo, Waltham, MA, USA), and protein identification was performed using the Mascot search engine (Matrix Science, London, UK; version 2.3.02) against the UniProt *Glycine max* (soybean) database. The minimum requirements for DEPs were: at least one matched unique peptide^22,45^ and a significant change (p ≤ 0.05 and ≥1.5-fold or ≤0.67-fold change)^19,23,46^ in protein quantities in at least two repetitions. MATLAB software was used for the hierarchical clustering (HC) analysis of the quantitative data from three biological replicates. The biological process, molecular function, and cellular component annotation of the identified DEPs were performed by utilizing blast v2.2.26 and Blast2GO v4.5 against the NCBI NR database and the most recent GO database^47^.

## Electronic supplementary material

Supplementary Information

## Data Availability

All data generated or analysed during this study are included in this published article (and its Supplementary Information files).
