# Supplementary material for: iTRAQ-based quantitative tissue proteomic analysis of differentially expressed proteins (DEPs) in non-transgenic and transgenic soybean seeds
Source: Sci Rep. 2018 Dec 5;8:17681. doi: 10.1038/s41598-018-35996-y (PMC6281665; doi:10.1038/s41598-018-35996-y)
Supplement: Supplementary file 1 — Supplementary Information [file 41598_2018_35996_MOESM1_ESM.pdf]

Supplementary Information

iTRAQ-based quantitative tissue proteomic analysis of differentially expressed proteins (DEPs) in non-transgenic and transgenic soybean seeds

Weixiao Liu[1, a], Wentao Xu[2, a], Liang Li[1], Mei Dong[1], Yusong Wan[1], Xiaoyun He[2], Kunlun Huang[2, **] and Wujun Jin[1, *]

1 Biotechnology Research Institute, Chinese Agricultural and Academic Sciences, Beijing 100081, PR China

2 Laboratory of Food Safety and Molecular Biology, College of Food Science and Nutritional Engineering, China Agricultural University, Beijing 100083, PR China

[a] These authors contributed equally to this work.

[*] Correspondence and requests for materials should be addressed to W.J. (email: Jinwujun@caas.cn).

[**] Correspondence and requests for materials should be addressed to K.H. (email: hkl009@163.com).

Figure S1

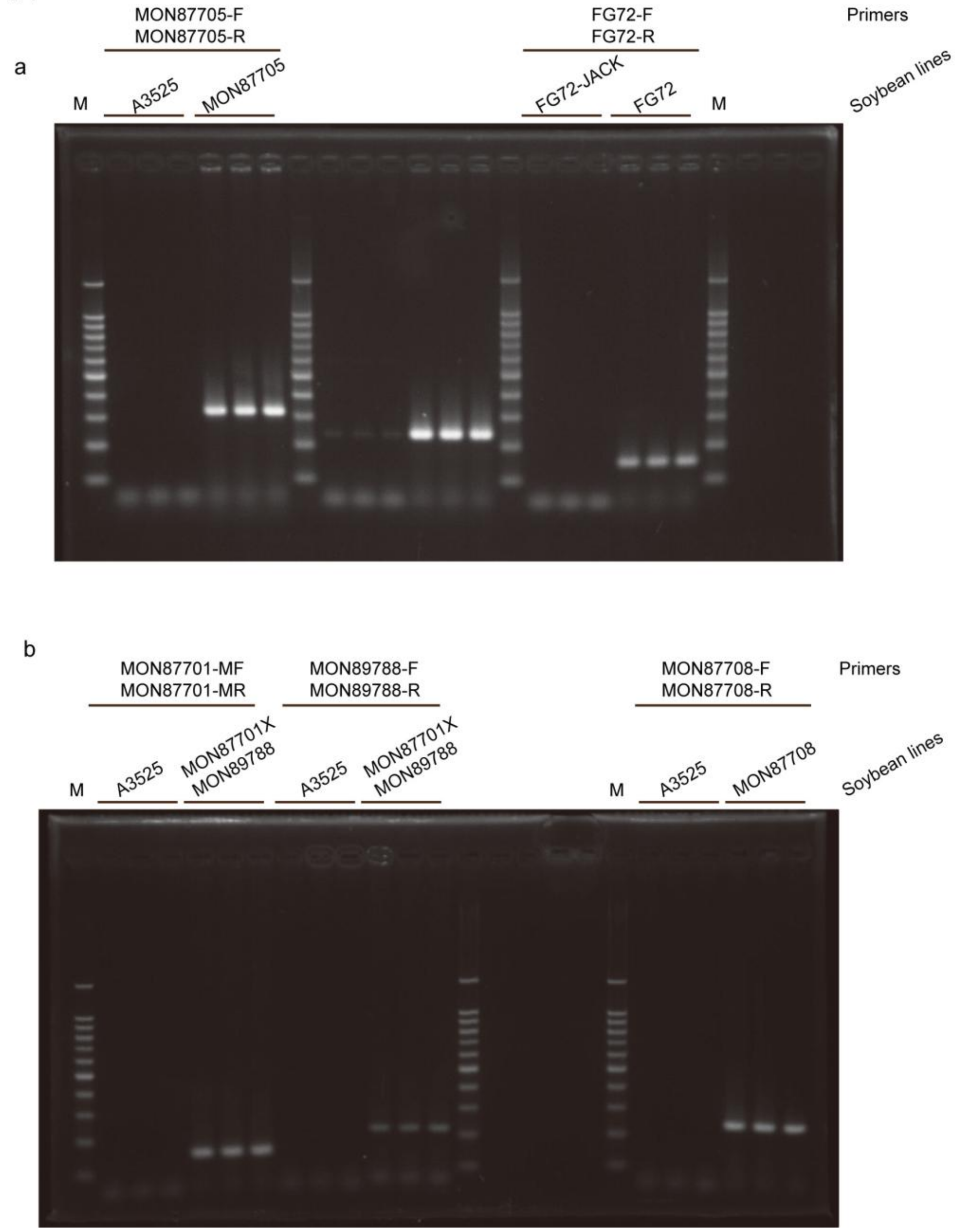

Figure S1. Event-specific PCR detection of the soybean lines studied. M, 100-bp marker. (a), Event-specific PCR of the soybean lines MON87705 and FG72. (b), Event-specific PCR of the soybean lines MON87701×MON89788 and MON87708.

Figure S2

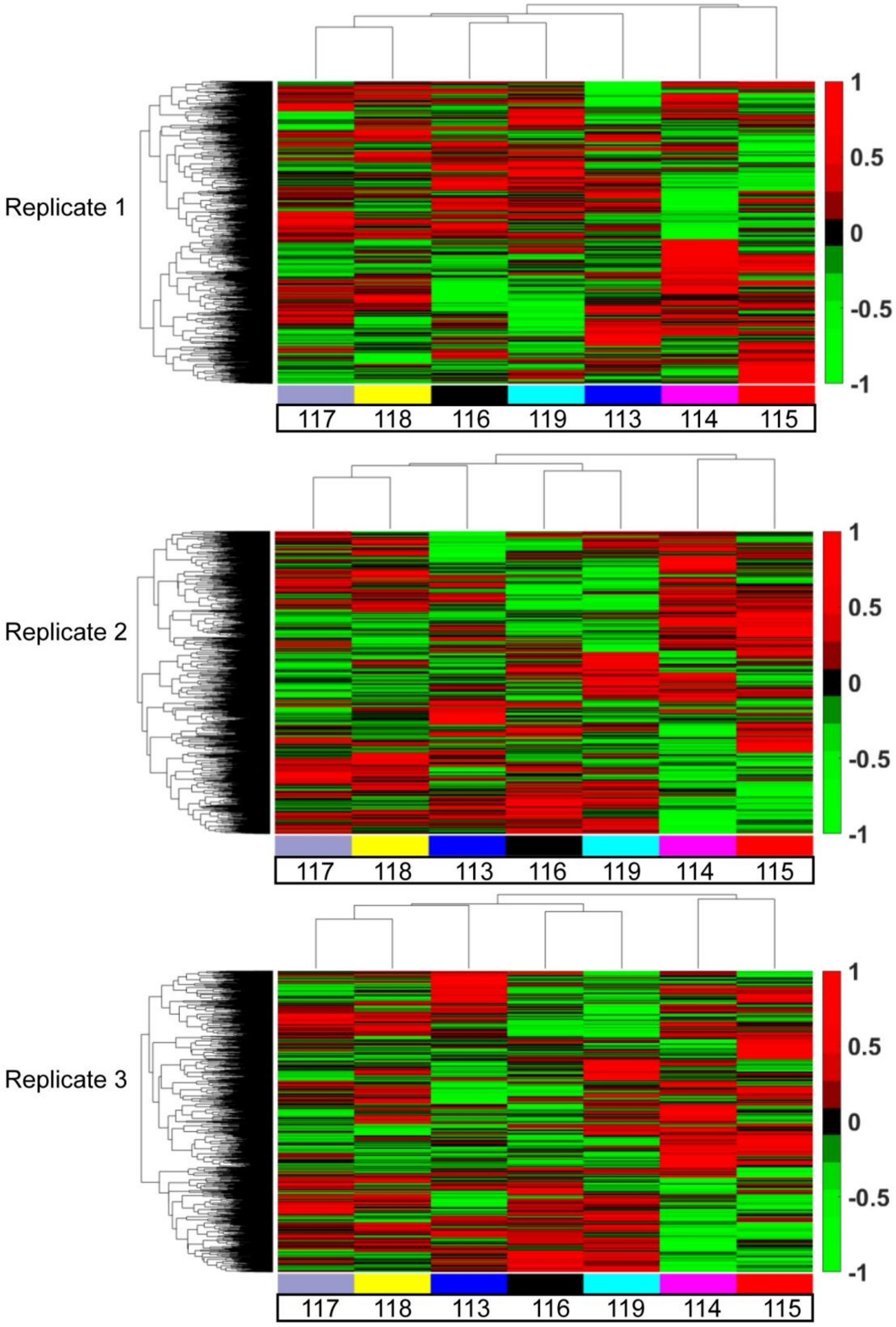

Figure S2. Clustering analysis of data from 3 replicates. All MS data were normalized and then subjected to the clustering analysis. Cluster results were visualized by using TreeView software.

Table S1. The indentified soybean seed cotyledon proteins from 3 iTRAQ replicates.

Accession	Description	MW [kDa]	calc. pI
P04405	Glycinin G2 OS	54.4	5.58
Q84XV8	FGAM synthase OS	113.8	5.01
A0A0R0FJR0	40S ribosomal protein SA OS	34.0	5.17
A0A0R0J3D8	40S ribosomal protein SA OS	30.9	5.88
I1L378	Uncharacterized protein OS	17.9	5.07
I1K5E6	Uncharacterized protein OS	35.6	7.72
C6TJD3	Uncharacterized protein OS	35.7	7.72
I1NC93	Uncharacterized protein OS	35.8	4.60
Q852U4	Glycinin A1bB2-784 OS	54.3	6.35
A0A0B2QCP9	Brefeldin A-inhibited guanine nucleotide-exchange protein 2 OS	197.5	5.54
I1M5P9	Uncharacterized protein OS	107.1	7.64
C6T6L8	Putative uncharacterized protein OS	21.9	6.29
A0A0B2PSP9	Glycinin G2 OS	59.0	6.04
I1KT36	Uncharacterized protein OS	20.8	6.52
A0A0R0EDI1	Uncharacterized protein OS	30.9	7.06
I1JIA6	Uncharacterized protein OS	7.7	9.72
C6T6P9	40S ribosomal protein S21 OS	9.1	6.77
A0A0B2P319	60S ribosomal protein L6 (Fragment) OS	23.3	10.04
A0A0B2SUD0	Thylakoid membrane phosphoprotein 14 kDa, chloroplastic OS	16.0	8.46
K7MGR3	Uncharacterized protein OS	14.1	9.31
K7MMC4	Uncharacterized protein OS	8.1	4.88
I1KVS3	Uncharacterized protein OS	7.4	8.91
A0A0B2Q7M5	Uncharacterized protein OS	7.2	9.47
A0A0B2PKL0	Defensin-like protein (Fragment) OS	6.6	8.35
Q04672	Sucrose-binding protein OS	60.5	6.87
I1JLC8	Protein SLE2 OS	11.5	5.60
A0A0B2SE14	Low-temperature-induced 65 kDa protein OS	62.1	6.58
A0A0R0G538	Uncharacterized protein OS	26.2	4.74
I1JDR2	Uncharacterized protein OS	116.4	6.81
A0A0B2SQI5	Uncharacterized protein OS	9.4	6.76
C6ZJY8	Serine hydroxymethyltransferase OS	57.4	7.97
C6TAX7	Uncharacterized protein OS	24.2	5.77
K7MNF4	Uncharacterized protein OS	58.3	7.81
Q9FQE9	Glutathione S-transferase GST 9 OS	23.4	6.00
P45458	Malate synthase, glyoxysomal (Fragment) OS	63.9	7.37
C6T9W4	Putative Fe(II)-and 2-oxoglutaratedependent dioxygenase OS	39.4	5.47
C6T9J8	Putative uncharacterized protein OS	23.5	5.31
A0A0B2NW50	Lipoxygenase OS	94.3	6.73
C6SVF6	Uncharacterized protein OS	11.3	6.52
C6SX26	Putative uncharacterized protein OS	12.1	5.82

I1LCI1	Uncharacterized protein OS	61.4	5.90
A0A0R0G0I7	Uncharacterized protein OS	34.7	5.20
A0A0B2R7H8	Protein LURP-one-related 15 OS	14.0	6.38
K7KU09	ATP-dependent 6-phosphofructokinase OS	55.9	6.76
O22378	Metallothionein-II protein OS	8.3	7.33
I1L5Z8	Uncharacterized protein OS	30.7	4.73
C6TNA5	Superoxide dismutase [Cu-Zn] OS	16.5	7.37
C6SWE8	Superoxide dismutase [Cu-Zn] OS	15.2	6.24
I1JL08	Non-specific lipid-transfer protein OS	12.3	9.36
A0A0B2QLA9	Peroxiredoxin-2B OS	17.4	5.59
A0A0B2P492	Histone deacetylase complex subunit SAP18 OS	11.6	10.32
A0A0B2S8E2	Tripeptidyl-peptidase 2 OS	146.6	6.16
A0A0B2S5Z1	Acetyltransferase component of pyruvate dehydrogenase complex OS	59.6	7.68
A0A0B2SFR9	Putative glycerophosphoryl diester phosphodiesterase 3 OS	81.8	6.20
A0A0R0ENW6	Uncharacterized protein OS	46.3	8.53
K7M324	Uncharacterized protein OS	15.3	5.05
C6T1V2	Uncharacterized protein OS	17.7	6.81
A0A0B2QMF0	Delta-1-pyrroline-5-carboxylate synthase (Fragment) OS	67.4	5.87
Q9XET1	Seed maturation protein PM31 OS	17.7	6.55
A0A0B2Q4Z1	Elongation factor 1-alpha OS	49.2	9.07
I1LRP3	Uncharacterized protein OS	25.0	9.03
A0A0R0HXF6	Uncharacterized protein OS	13.5	9.89
I1JXM1	HVA22-like protein OS	16.9	9.03
A0A0R0I177	Uncharacterized protein OS	46.8	5.91
C6TF65	Putative uncharacterized protein OS	17.6	5.36
C6T7B0	Putative uncharacterized protein (Fragment) OS	48.5	5.78
A0A0R0H1C4	Uncharacterized protein OS	15.1	9.95
A0A0B2NVP3	Glycosyltransferase OS	52.2	6.07
I1K6M2	Uncharacterized protein OS	23.8	7.87
I1MYI8	Glyceraldehyde-3-phosphate dehydrogenase OS	36.8	7.47
A0A0B2Q9T2	Chaperone protein ClpB1 OS	42.9	6.30
P28759	Superoxide dismutase [Fe], chloroplastic OS	27.8	5.87
A0A0B2RJR9	Urease OS	90.0	6.13
A0A0B2PKZ5	Vacuolar-processing enzyme OS	55.1	6.19
I1JQF4	Uncharacterized protein OS	46.5	5.31
A0A0R0I9J5	Uncharacterized protein OS	11.8	8.43
A0A0R0EDH2	Uncharacterized protein (Fragment) OS	56.4	5.55
I1JSN3	Uncharacterized protein OS	27.9	7.55
A0A0B2RV43	Mitochondrial outer membrane protein porin 2 OS	29.1	9.23
C6TAY5	Putative uncharacterized protein OS	37.3	6.30
A0A0B2PJF4	Uncharacterized protein OS	153.3	4.70
A0A0R0K6C1	Uncharacterized protein OS	61.6	4.91

A0A0B2PX90	Bifunctional aspartokinase/homoserine dehydrogenase, chloroplastic OS	100.3	7.74
C6TFT6	4-hydroxy-4-methyl-2-oxoglutarate aldolase OS	15.8	6.54
I1N583	Uncharacterized protein OS	48.5	6.01
A0A0B2S2I4	Nicalin-1 (Fragment) OS	61.1	6.40
C6TIY1	Uncharacterized protein OS	21.8	4.55
A0A0B2Q9B8	Oleosin OS	17.5	8.06
Q42785	Nonsymbiotic hemoglobin OS	18.0	8.92
A0A0R0K737	6-phosphogluconate dehydrogenase, decarboxylating OS	52.2	6.70
A0A0B2QVE6	Beta-glucosidase 44 OS	51.9	9.32
A0A0B2S829	Reticulon-like protein OS	28.8	7.65
P13917	Basic 7S globulin OS	46.4	8.32
I1NFH5	Uncharacterized protein OS	48.9	8.70
A0A0B2RCN5	Transmembrane 9 superfamily member OS	73.1	7.62
C6SVT0	Uncharacterized protein OS	18.0	7.15
H2D5S3	Malate dehydrogenase OS	35.2	6.30
K7LIS9	Uncharacterized protein OS	9.5	9.00
Q01527	Maturation protein OS	15.6	9.66
C6THC7	Putative uncharacterized protein OS	35.8	4.73
I1N466	Uncharacterized protein OS	39.9	6.30
C6TFC1	Non-specific lipid-transfer protein OS	12.4	8.63
K7L4F4	Uncharacterized protein OS	104.1	6.04
I1MIA8	Uncharacterized protein OS	15.2	4.64
D2DJQ5	Aspartate-semialdehyde dehydrogenase (Fragment) OS	37.6	6.33
I1MQB5	Uncharacterized protein OS	26.2	9.64
C6T107	Uncharacterized protein OS	13.0	4.94
I1JJB5	Uncharacterized protein OS	50.5	9.23
C6SXJ9	Cytochrome b-c1 complex subunit 7 OS	14.6	9.61
A0A0B2PMM9	E3 ubiquitin-protein ligase UPL1 OS	393.9	5.40
A0A0B2PHB3	Eukaryotic translation initiation factor 4E-1 OS	26.3	5.17
A0A0B2SI48	Alcohol dehydrogenase 1 OS	41.2	6.95
A0A0B2PFY3	60S acidic ribosomal protein P3 OS	11.9	4.46
A0A0B2RM77	Cysteine proteinase inhibitor OS	12.4	9.03
K7LWN2	Uncharacterized protein OS	10.5	4.84
F7J077	Beta-conglycinin beta subunit OS	50.4	6.24
Q7M1R5	Superoxide dismutase [Cu-Zn] OS	15.2	5.60
K7N4A7	Uncharacterized protein OS	27.6	6.05
C6TH20	Uncharacterized protein OS	39.4	6.19
C6SWW7	Putative uncharacterized protein OS	19.7	5.39
B1Q2X5	Protein disulfide-isomerase OS	61.6	4.84
K7MLV5	Uncharacterized protein OS	46.2	5.05
I1LRA0	Uncharacterized protein OS	54.5	8.05
I1NGJ9	1,2-dihydroxy-3-keto-5-methylthiopentene dioxygenase OS	22.5	5.24

I1J8Y8	Uncharacterized protein OS	16.8	8.15
A0A0B2QYX5	60S acidic ribosomal protein P2B OS	9.0	4.77
A0A0B2QNW3	Ras-related protein RABE1c OS	23.9	7.83
A0A0B2RHI0	Lipoxygenase OS	96.7	6.58
I1MAE7	Acyl-[acyl-carrier-protein] desaturase OS	38.6	5.97
A0A0R4J2L9	Malate dehydrogenase OS	35.4	6.30
C6T1F3	Putative uncharacterized protein OS	12.1	9.61
I1N6T0	Uncharacterized protein OS	22.3	9.76
A0A0B2SEG7	Leucine--tRNA ligase, cytoplasmic OS	97.0	6.38
I1KPE7	Uncharacterized protein OS	19.9	9.67
K7L453	Uncharacterized protein OS	27.5	9.23
C6TB95	Uncharacterized protein OS	15.9	5.64
A0A0B2RCV2	Puromycin-sensitive aminopeptidase OS	98.0	5.63
A0A0B2RQ64	FAS-associated factor 2-B OS	65.8	4.73
I1K3Q1	Cysteine proteinase inhibitor OS	14.2	9.13
A0A0R0L186	Uncharacterized protein OS	17.3	7.42
I1LUM3	Uncharacterized protein OS	29.3	4.82
I1KZJ1	Uncharacterized protein OS	43.0	5.54
A0A0B2RIW3	Kunitz-type trypsin inhibitor KTI1 OS	17.7	5.36
O64458	34 kDa maturing seed protein OS	42.7	5.99
I1LJX3	Uncharacterized protein OS	16.2	5.99
C6TAJ8	Putative uncharacterized protein OS	53.5	6.04
A0A0B2Q229	Carboxypeptidase OS	50.9	6.47
Q8L7J5	Pyruvate kinase OS	55.3	7.75
I1LEC4	Uncharacterized protein OS	49.4	5.10
C6TGA6	60S acidic ribosomal protein P0 OS	34.2	5.11
C6SZN6	Uncharacterized protein OS	17.9	5.91
A0A0B2R847	2S albumin OS	17.8	6.38
C6SVM2	Putative uncharacterized protein OS	17.4	8.94
C6T6U5	Putative uncharacterized protein (Fragment) OS	19.1	8.90
I1M5K0	Uncharacterized protein OS	55.9	5.24
A0A0R0HZK7	Uncharacterized protein OS	68.3	6.92
A0A0B2S4F2	Proliferation-associated protein 2G4 OS	43.3	6.86
C6SXG5	Uncharacterized protein OS	15.8	7.56
A0A0R4J4C5	Uncharacterized protein OS	23.1	5.68
K7MB76	Uncharacterized protein OS	20.6	5.30
A0A0B2P4A9	Aldo-keto reductase family 4 member C9 OS	35.1	6.14
I1JXS8	Uncharacterized protein OS	108.9	6.49
A0A0R0G3L8	Phospho-2-dehydro-3-deoxyheptonate aldolase OS	54.9	8.28
C6T9Z8	Putative uncharacterized protein OS	26.0	7.75
A0A0B2P7M4	Elongation factor 1-gamma OS	47.7	6.71
I1KYW3	Uncharacterized protein OS	22.6	7.24
I1MRV6	Uncharacterized protein OS	52.9	7.34

I1MEF8	Uncharacterized protein OS	35.3	5.19
A0A0B2R7V1	PRA1 family protein D OS	18.1	5.76
I1JN67	Uncharacterized protein OS	29.7	6.15
C6TBX7	Uncharacterized protein OS	9.7	9.79
A0A0R0G6T3	Uncharacterized protein OS	27.2	6.23
B3TDK5	Lipoxygenase OS	96.7	6.64
A0A0R0GA14	Uncharacterized protein OS	17.9	7.50
I1JDU8	Uncharacterized protein OS	65.1	5.22
C6TKL2	Reticulon-like protein OS	28.5	8.35
C6TCU7	Homoserine dehydrogenase OS	40.6	6.84
K7LSM3	Uncharacterized protein OS	148.4	4.69
C6SZX7	Glutathione peroxidase OS	18.5	7.09
A0A0B2Q5M9	3-hydroxyisobutyryl-CoA hydrolase-like protein 3, mitochondrial OS	41.0	5.94
K7LZH1	Uncharacterized protein OS	69.2	5.25
A0A0B2QJ68	14-3-3-like protein OS	29.1	4.75
I1MVS9	Uncharacterized protein OS	43.8	7.65
C6SY24	Putative uncharacterized protein OS	24.9	6.07
I1KDL8	Uncharacterized protein OS	13.6	6.55
C6TKQ3	Uncharacterized protein OS	34.7	6.37
I1MXZ6	Uncharacterized protein OS	13.4	4.88
I1NC78	Uncharacterized protein OS	27.7	7.96
I1LY10	Uncharacterized protein OS	39.6	4.41
C3VHQ8	Oleosin OS	17.4	8.06
A0A0B2PI28	Vesicle-associated membrane protein 722 OS	11.9	9.14
A0A0R0HM79	Uncharacterized protein OS	50.3	7.12
Q38IW8	Triosephosphate isomerase OS	27.2	6.23
A0A0B2QX34	Translation machinery-associated protein 22 OS	19.7	5.02
A0A0B2RG57	Proline iminopeptidase OS	37.7	5.55
C6T9T3	Putative uncharacterized protein (Fragment) OS	18.0	5.69
I1LB97	Uncharacterized protein OS	14.1	9.55
I1M0W1	Uncharacterized protein OS	15.1	5.86
I1LCI3	Superoxide dismutase OS	27.5	5.66
A0A0B2PPY9	Protein transport protein Sec24-like OS	117.4	7.83
C6T034	Uncharacterized protein OS	24.3	4.68
K7MBV6	Uncharacterized protein OS	31.9	10.05
A0A0R4J3M4	Uncharacterized protein OS	65.1	6.27
A0A0B2SQD7	Phosphoglycerate kinase OS	42.4	6.32
K7M8I2	Uncharacterized protein OS	12.3	4.70
A0A0R0JK36	Uncharacterized protein OS	19.3	8.95
I1NHB5	Uncharacterized protein OS	48.4	8.63
I1N3G2	Uncharacterized protein OS	42.5	6.34
A0A0R0GFW6	Phosphoglycerate kinase OS	50.0	8.15

A0A0B2QCE3	Leucine aminopeptidase 3, chloroplastic OS	58.1	5.91
I1MUN6	Uncharacterized protein OS	52.5	7.18
C6T020	Glutathione peroxidase OS	18.7	5.72
A0A0B2QXK7	Pyruvate kinase OS	57.6	6.76
A0A0R0GZI4	Uncharacterized protein OS	17.4	7.50
I1K1N3	Methionine aminopeptidase OS	40.2	5.91
I1M6P9	Uncharacterized protein OS	49.8	9.16
I1MUZ3	Uncharacterized protein OS	18.9	8.76
C6SW37	Uncharacterized protein OS	7.1	9.54
Q42800	4-hydroxy-tetrahydrodipicolinate synthase, chloroplastic OS	36.4	6.32
A0A0B2PVA2	Coatomer subunit delta OS	55.9	5.59
A0A0B2Q8X3	Uncharacterized protein OS	27.6	6.87
A0A0B2SNS8	Fructose-bisphosphate aldolase OS	38.3	7.55
A0A0R0J9Y0	Uncharacterized protein OS	77.9	5.99
I1N7Y5	Uncharacterized protein OS	15.3	9.72
C6ZHS4	Eukaryotic translation initiation factor 5A OS	17.5	5.99
I1JGP8	Uncharacterized protein OS	59.6	7.12
I1LMH7	Uncharacterized protein OS	22.2	7.93
A0A0R0EDR0	Uncharacterized protein OS	15.7	7.56
A0A0R4J4U4	Uncharacterized protein OS	41.1	6.99
C6SYT7	Glutathione peroxidase (Fragment) OS	25.0	8.79
I1JAY8	Uncharacterized protein OS	71.2	5.77
A0A0B2RX31	Far upstream element-binding protein 2 OS	69.0	5.88
A0A0B2QUL3	Tropinone reductase like OS	28.4	6.11
K7KBN4	Uncharacterized protein OS	21.2	5.39
C6T420	Putative uncharacterized protein OS	17.4	7.12
A0A0B2RE45	Cytochrome c oxidase subunit 5C-2 OS	7.1	8.59
I1K7N6	Uncharacterized protein OS	13.0	5.88
C6TLC1	Putative uncharacterized protein OS	18.3	5.03
I1K3S5	Adenosylhomocysteinase OS	53.2	5.88
I1JPW5	Uncharacterized protein OS	47.6	5.69
I1K5Z3	Uncharacterized protein OS	34.9	8.66
I1JW44	Uncharacterized protein OS	46.9	8.54
C6TJ08	Putative uncharacterized protein OS	35.2	6.27
A0A0B2PWX8	Agmatine coumaroyltransferase OS	56.6	5.94
Q9S7N8	Seed maturation protein PM21 OS	10.1	5.01
Q42795	Beta-amylase OS	56.0	5.55
C6TCR6	Putative uncharacterized protein OS	36.2	5.07
C6T6Q8	Putative uncharacterized protein (Fragment) OS	22.9	8.25
A0A0B2PPT4	Nuclease domain-containing protein 1 OS	108.8	6.55
I1LGD6	Uncharacterized protein OS	19.8	9.64
A0A0B2QY19	Uncharacterized protein OS	27.5	6.54
A0A0B2QUJ7	Cytochrome b5 isoform 1 OS	13.3	5.01

I1KU74	Uncharacterized protein OS	12.7	6.14
A0A0R0HEL8	Peptidyl-prolyl cis-trans isomerase OS	18.2	8.46
I1JCG0	Uncharacterized protein OS	42.8	4.67
A0A0B2R6N4	Lipoxygenase OS	94.4	6.33
C6TLN8	Putative uncharacterized protein OS	26.8	6.68
I1N696	Uncharacterized protein OS	54.9	5.63
I1LD41	Uncharacterized protein OS	19.0	7.30
A0A0B2QYT0	GDSL esterase/lipase OS	39.8	9.20
I1MTN1	Uncharacterized protein OS	107.3	5.62
I1K4B0	Uncharacterized protein OS	32.3	5.68
A0A0B2S880	5-methyltetrahydropteroyltriglutamate--homocysteine methyltransferase OS	88.6	6.77
I1MAE6	Uncharacterized protein OS	40.8	6.52
A0A0R0KP81	Uncharacterized protein OS	23.5	8.73
C6SVD3	Peptidyl-prolyl cis-trans isomerase OS	12.0	6.01
A0A0R0JRB0	L-lactate dehydrogenase OS	37.8	7.02
C6SXJ2	Uncharacterized protein OS	15.5	9.83
A0A0B2S0V2	Ribonuclease UK114 OS	18.0	5.60
I1KD10	Uncharacterized protein OS	28.0	10.14
I1N6Q6	Ribulose bisphosphate carboxylase small chain OS	19.1	8.43
A0A0B2RZX6	Fructose-bisphosphate aldolase OS	38.3	7.53
A0A0R0KRW0	Uncharacterized protein OS	43.4	5.22
C6T2S4	Putative uncharacterized protein OS	22.2	7.47
C6TBW6	Putative uncharacterized protein OS	11.9	4.56
K7KU59	Glucose-6-phosphate isomerase OS	64.9	5.63
C3VLR2	Citrate synthase (Fragment) OS	22.4	5.62
A0A0B2QC89	Defensin-like protein (Fragment) OS	6.2	9.19
K7LRU7	Uncharacterized protein OS	77.7	4.89
I1MLP0	Glucose-6-phosphate 1-dehydrogenase OS	53.9	6.29
I1K8S6	Uncharacterized protein OS	18.4	8.43
A0A0B2RXW0	Stem 28 kDa glycoprotein OS	26.7	8.65
Q9XET0	Seed maturation protein PM30 OS	15.1	8.90
I1KTY9	Sulfurtransferase OS	41.8	6.86
A0A0B2S5K3	Uncharacterized protein OS	5.5	4.59
C6TCN5	Ferritin OS	28.1	6.04
A0A0R0K398	Uncharacterized protein OS	37.4	8.09
I1M561	Uncharacterized protein OS	35.4	5.45
I1LN49	Uncharacterized protein OS	27.0	7.06
C6TKJ1	Putative uncharacterized protein OS	45.6	7.36
A0A0B2Q2E1	Putative glutamate--tRNA ligase, cytoplasmic OS	82.8	7.24
A0A0R0K666	Uncharacterized protein OS	47.0	8.12
A0A0B2S007	UDP-glycosyltransferase 71C4 OS	52.4	5.99
I1L8G3	Succinyl-CoA ligase subunit beta OS	45.3	6.23

A0A0B2NYX7	Putative aquaporin PIP2-1 OS	20.7	9.95
C6TG97	Proteasome subunit alpha type OS	27.2	5.87
A0A0B2QC63	Nodal modulator 1 OS	130.4	5.60
I1KK63	Uncharacterized protein OS	25.3	6.24
I1N1F9	Uncharacterized protein OS	58.3	7.15
A0A0B2PSI9	Putative calcium-binding protein CML27 OS	15.8	5.20
Q2XSE5	Seed maturation protein OS	14.5	9.10
A0A0B2PWM7	Uncharacterized protein OS	11.3	5.05
K7LSI7	Uncharacterized protein OS	82.4	6.61
A0A0B2SLD1	Ribokinase OS	15.4	6.11
C6TK60	Putative uncharacterized protein OS	19.1	4.55
I1KYT6	RuvB-like helicase OS	51.0	5.47
C6SZA3	Uncharacterized protein OS	8.5	6.60
I1KTM3	Uncharacterized protein OS	42.0	9.61
I1KQW4	Uncharacterized protein OS	75.4	6.10
A0A0R0GK20	Uncharacterized protein (Fragment) OS	4.2	4.46
K7L6V9	Uncharacterized protein OS	12.2	6.16
A0A0B2QWA5	LysM domain-containing GPI-anchored protein 2 OS	38.6	7.03
A0A0R0FLE5	Ubiquitin carboxyl-terminal hydrolase OS	20.8	4.68
I1M984	Uncharacterized protein OS	60.2	6.10
C6SWU2	Putative uncharacterized protein OS	17.7	4.81
I1KJJ8	Uncharacterized protein OS	33.2	8.06
K7KKV5	Uncharacterized protein OS	7.7	5.14
C6SVK3	Uncharacterized protein OS	22.7	5.27
I1KAD3	Uncharacterized protein OS	79.9	6.67
A0A0R0I0B9	Uncharacterized protein OS	26.3	9.55
C6TN99	Putative uncharacterized protein OS	34.5	6.44
I1NFH9	Uncharacterized protein OS	37.4	4.82
I1KKN7	Pyrophosphate--fructose 6-phosphate 1-phosphotransferase subunit alpha OS	67.4	7.27
C6TK02	Putative uncharacterized protein OS	47.1	6.01
I1MFN0	Uncharacterized protein OS	45.8	6.28
A0A0R0KF79	Uncharacterized protein OS	39.9	9.09
C6T2Q6	Putative uncharacterized protein OS	26.8	6.29
A0A0B2PJM6	Seed biotin-containing protein SBP65 OS	117.8	8.51
A0A0B2QKG4	Mitochondrial Rho GTPase OS	71.8	5.54
D2DJQ6	Branched-chain-amino-acid aminotransferase (Fragment) OS	35.9	6.58
K7M836	Uncharacterized protein OS	23.1	6.79
A0A0R0EE15	Chorismate synthase OS	41.5	6.54
C6T1P5	Uncharacterized protein OS	15.5	9.83
I1N5P1	Uncharacterized protein OS	19.9	5.77
I1KWT3	Uncharacterized protein OS	37.5	6.37
I1JY56	Uncharacterized protein OS	41.7	5.49

C6K8D1	Seed biotinylated protein 68 kDa isoform OS	67.9	6.58
A1KR24	Dehydrin OS	25.4	6.61
A0A0R0K4D6	Uncharacterized protein (Fragment) OS	16.1	9.31
I1NJA4	Uncharacterized protein OS	58.1	7.02
A0A0R0KRC0	Serine/threonine-protein phosphatase OS	28.7	5.06
K7KHU3	Uncharacterized protein OS	21.6	5.47
K7KWG2	Uncharacterized protein OS	6.5	5.19
A0A0B2PAJ8	Starch synthase, chloroplastic/amyloplastic OS	68.0	7.06
P05046	Lectin OS	30.9	6.05
I1M7M8	Uncharacterized protein OS	35.0	6.81
A0A0R0KLT7	Uncharacterized protein OS	71.0	6.18
P27066	Ribulose bisphosphate carboxylase large chain OS	52.6	6.43
A0A0B2SAB8	Aconitate hydratase OS	98.6	6.24
I1NBD9	Uncharacterized protein OS	95.2	6.02
C6TLQ4	Eukaryotic translation initiation factor 6 OS	26.6	4.74
I1LQR8	Uncharacterized protein OS	26.2	7.85
I1N3E7	Uncharacterized protein OS	46.5	8.43
C6SWA9	Uncharacterized protein OS	11.4	4.41
C6SZ13	Uncharacterized protein OS	15.8	9.36
I1JCM5	Uncharacterized protein OS	79.4	5.66
A0A0B2Q0T9	Pectinesterase OS	30.6	6.06
A0A0B2S9A1	Basic 7S globulin 2 OS	40.0	8.12
I1LIK8	Uncharacterized protein OS	134.8	6.67
A0A0B2SAW3	Aconitate hydratase OS	98.3	5.99
I1MTU1	Malate dehydrogenase OS	38.3	6.27
A0A0R0H0L6	Defective in cullin neddylation protein OS	21.4	7.71
K7N388	Uncharacterized protein OS	6.1	9.77
I1NEH4	Uncharacterized protein OS	36.0	6.77
A0A0R0JQV7	Uncharacterized protein OS	59.3	4.94
C6SXH8	Putative uncharacterized protein OS	25.9	7.77
A0A0B2PMP3	40S ribosomal protein S17 (Fragment) OS	12.4	10.18
I1M5V9	Uncharacterized protein OS	25.2	8.32
K7KQG4	Uncharacterized protein OS	78.2	4.83
I1L700	Uncharacterized protein OS	64.7	5.43
C6SXW1	Uncharacterized protein OS	15.1	6.74
A0A0B2QXH5	Polygalacturonase inhibitor OS	36.6	8.31
A0A0B2RJS3	Protein disulfide isomerase-like 1-4 OS	62.6	5.01
I1J6K4	Uncharacterized protein OS	102.9	6.70
I1KY71	Uncharacterized protein OS	75.5	5.35
A0A0B2PJ13	Adipocyte plasma membrane-associated protein OS	31.7	5.21
Q1WAB8	Glycinin OS	63.1	5.95
A0A0B2SQZ3	TOM1-like protein 2 OS	55.8	5.06
A0A0R4J4X3	Cysteine proteinase inhibitor OS	10.7	6.09

A0A0B2R0Q8	Altered inheritance of mitochondria protein 32 (Fragment) OS	35.6	7.20
A0A0R0HHB6	Uncharacterized protein OS	17.8	8.97
I1NC66	Uncharacterized protein OS	24.7	7.55
I1N747	Oleosin OS	23.6	8.94
I1KGP0	Uncharacterized protein OS	28.4	9.99
A0A0B2QKB2	Sulfite reductase [ferredoxin] (Fragment) OS	75.8	9.11
I1MS47	Uncharacterized protein OS	23.3	9.41
I1M4K9	Protein disulfide-isomerase OS	62.3	4.87
I1K5F0	Uncharacterized protein OS	53.3	5.49
K7KFX8	Uncharacterized protein OS	17.0	5.22
A0A0B2QNX0	Uncharacterized protein OS	34.3	6.79
C6TL66	Putative uncharacterized protein OS	24.2	9.19
A0A0B2RH65	Chaperone protein ClpC, chloroplastic OS	100.9	6.64
I1JML1	Uncharacterized protein OS	20.8	7.58
C6T871	Uncharacterized protein OS	40.1	6.68
A0A0R0IIZ3	Uncharacterized protein OS	57.8	9.19
A0A0R0JL19	Uncharacterized protein OS	41.0	6.40
C6TN55	Putative uncharacterized protein OS	43.5	7.06
P28551	Tubulin beta chain (Fragment) OS	45.7	5.96
A0A0R0KUQ0	Eukaryotic translation initiation factor 3 subunit H OS	37.3	4.78
A0A0B2RFQ6	Oxysterol-binding protein-related protein 3B OS	34.3	5.54
I1K3E1	Uncharacterized protein OS	65.9	8.69
I1M7B9	Uncharacterized protein OS	60.2	6.58
A0A0B2P3S7	Peroxiredoxin-2E, chloroplastic OS	19.6	5.24
A0A0R0K869	Uncharacterized protein OS	18.0	9.99
I1MEH3	Uncharacterized protein OS	56.6	5.96
I1KQC5	Uncharacterized protein OS	14.5	10.04
C6TG44	Putative uncharacterized protein OS	38.4	5.16
I1N5I8	Oleosin OS	19.9	9.20
A0A0B2RVL7	Aldehyde oxidase 2 OS	147.9	6.64
I1JVP6	Uncharacterized protein OS	36.8	4.68
I1N5B5	Mannose-6-phosphate isomerase OS	46.2	5.96
I1JYP4	Uncharacterized protein OS	84.6	4.74
I1KR17	Uncharacterized protein OS	32.5	8.76
A0A0R4J3L4	Uncharacterized protein OS	27.4	6.30
A0A0R0K474	Uncharacterized protein OS	46.6	5.48
A0A0B2SSJ4	Histone H3.2 OS	12.4	10.51
C6TFP4	Uncharacterized protein OS	13.6	6.34
A0A0B2P3Y6	Uncharacterized protein OS	28.4	7.06
K7LFV9	Uncharacterized protein OS	20.4	8.69
A0A0B2NS26	Nucleoside diphosphate kinase OS	18.2	6.80
I1KWM7	6-phosphogluconate dehydrogenase, decarboxylating OS	53.5	6.48
C6TKA3	NADH-cytochrome b5 reductase OS	35.1	8.56

A0A0B2SME9	Sorting nexin 1 OS	46.2	8.25
I1LDV5	Uncharacterized protein OS	120.2	6.47
I1MQ52	Uncharacterized protein OS	35.2	5.68
K7L7P5	Uncharacterized protein OS	28.4	4.28
A0A0R0H1B4	Uncharacterized protein OS	12.5	5.53
C6SVH1	Putative uncharacterized protein OS	15.9	10.13
A0A0B2RC63	Alpha-1,4-glucan-protein synthase [UDP-forming] OS	41.5	5.97
C6T481	Putative uncharacterized protein OS	10.1	10.14
C6SVL1	Ribosomal protein OS	24.6	9.82
A3KEY7	Beta-conglycinin alpha' subunit (Fragment) OS	62.9	5.26
I1KMS6	Uncharacterized protein OS	19.0	7.50
I1JDH6	Uncharacterized protein OS	30.2	10.36
A0A0R0G7L1	Uncharacterized protein OS	12.9	8.07
A0A0B2PRA7	Uncharacterized protein OS	110.3	6.93
A0A0R0KKU3	Uncharacterized protein OS	71.4	5.16
A0A0B2PT76	Transaldolase OS	48.0	7.09
I1L6Y9	Alpha-galactosidase OS	45.6	7.15
K7M6Q3	Uncharacterized protein OS	32.0	8.94
I1LS55	Uncharacterized protein OS	39.7	9.80
C6T1S0	Putative uncharacterized protein OS	21.8	8.59
A0A0B2QQZ7	Selenium-binding protein 1 OS	53.4	6.09
A0A0B2SH03	Flavin-containing monooxygenase OS	39.5	8.91
A0A0R0GZT5	Uncharacterized protein OS	27.3	6.10
P19594	2S albumin OS	18.4	5.34
I1JQL0	Uncharacterized protein OS	20.5	10.27
C6SW44	Uncharacterized protein OS	12.9	10.67
A0A0B2PMW8	Tubulin beta-1 chain OS	50.4	4.83
A0A0B2QR45	Proteasome subunit beta type OS	29.3	7.25
K7KHJ7	Uncharacterized protein OS	18.0	8.12
A0A0R4J5Z8	Uncharacterized protein OS	84.2	6.37
A0A0R0H141	Nascent polypeptide-associated complex subunit beta OS	17.5	8.32
A0A0B2PKU1	Monothiol glutaredoxin-S17 OS	53.7	5.34
C6TNT2	EF1Bgamma class glutathione S-transferase OS	47.6	6.24
I1LYA6	Uncharacterized protein OS	16.7	5.12
A0A0R0GTY7	Uncharacterized protein OS	26.1	8.12
A0A0B2RXW5	Uncharacterized protein OS	8.8	9.76
I1LTA4	EF1Bgamma class glutathione S-transferase OS	47.4	6.02
C6T3L5	Uncharacterized protein OS	16.9	4.98
I1MTE2	Uncharacterized protein OS	17.3	9.47
I1JB85	Uncharacterized protein OS	30.3	5.69
I1MXX5	Proteasome subunit alpha type OS	31.0	5.19
I1K5K3	Uncharacterized protein OS	29.8	10.32
Q9LLX2	Trypsin inhibitor OS	18.0	6.54

A0A0B2SGV9	NADH dehydrogenase [ubiquinone] iron-sulfur protein 5-B OS	6.6	5.30
A0A0R4J410	Obg-like ATPase 1 OS	44.4	6.80
A0A0B2R7A9	Uncharacterized protein OS	70.3	9.07
A0A0R0FN75	Uncharacterized protein (Fragment) OS	30.1	8.31
I1M1V8	Uncharacterized protein OS	63.4	6.11
A0A0R4J3X6	Ribosomal protein L37 OS	10.7	11.91
P01064	Bowman-Birk type proteinase inhibitor D-II OS	9.5	5.14
Q6PV94	Thioredoxin OS	14.8	6.54
I1L957	Uncharacterized protein OS	48.8	6.42
I1KQE3	Uncharacterized protein OS	17.4	10.20
Q39805	Dehydrin-like protein OS	23.7	6.58
A0A0B2SUN2	Putative GPI-anchored protein OS	20.5	7.53
C6T4J1	Uncharacterized protein OS	10.6	6.79
A0A0B2S0P7	Putative ATP synthase 24 kDa subunit, mitochondrial OS	21.9	5.57
Q9SPJ6	Maturation protein pPM32 OS	18.9	5.60
K7N254	Uncharacterized protein OS	24.4	8.09
Q01417	18 kDa seed maturation protein OS	17.6	9.58
A0A0B2PDU7	Peptide methionine sulfoxide reductase OS	20.8	5.41
A0A0R0F9R1	Uncharacterized protein (Fragment) OS	6.7	8.91
I1K547	Uncharacterized protein OS	39.5	7.36
A0A0B2PMP1	Uncharacterized protein OS	19.0	9.67
C6TKK5	Uncharacterized protein OS	22.1	6.55
A0A0B2P8D8	Glycinin G4 OS	63.8	5.25
K7N354	Uncharacterized protein OS	10.6	5.41
C6SZG4	Uncharacterized protein OS	8.1	9.99
I1LP44	Uncharacterized protein OS	36.6	5.72
A0A0R0H0R3	Uncharacterized protein (Fragment) OS	13.6	8.91
A8IKE1	Alanine aminotransferase 1 OS	53.3	5.45
I1MGG5	Uncharacterized protein OS	14.6	7.14
A0A0R0HC18	Uncharacterized protein OS	45.8	5.07
A0A0R0G797	Uncharacterized protein OS	43.2	6.95
A0A0B2PTF7	Enoyl-[acyl-carrier-protein] reductase [NADH], chloroplastic OS	39.7	8.53
A0A0B2S6T6	Glutamyl-tRNA reductase OS	58.3	8.34
A0A0B2RE59	Uncharacterized protein OS	40.3	5.92
I1LSB5	Uncharacterized protein OS	62.7	7.28
I1KTZ5	Proteasome subunit alpha type OS	25.0	8.48
I1MTK8	Uncharacterized protein OS	12.2	7.94
I1KXF3	Uncharacterized protein OS	21.0	6.92
B2CM89	Isoflavone synthase (Fragment) OS	56.2	9.10
P29531	P24 oleosin isoform B OS	23.4	8.94
I1J7U9	3-phosphoshikimate 1-carboxyvinyltransferase OS	55.8	8.07
I1N674	Uncharacterized protein OS	19.1	6.10

I1KVR7	Coatomer subunit beta OS	96.6	5.72
A0A0B2NUG1	Glutathione reductase, cytosolic OS	53.9	5.72
A0A0R0H2Z4	Uncharacterized protein OS	59.8	8.94
I1JWW7	Uncharacterized protein OS	21.5	10.21
I1KR53	Uncharacterized protein OS	82.5	7.66
I1KYB6	Uncharacterized protein OS	32.6	6.01
C6TD48	Putative uncharacterized protein (Fragment) OS	16.3	9.95
I1JP48	Uncharacterized protein OS	74.8	6.33
A0A0R0G924	Uncharacterized protein OS	128.2	5.50
A0A0R0HJ74	Uncharacterized protein OS	55.2	5.60
A0A0B2R4I4	Phosphate carrier protein, mitochondrial OS	36.3	9.13
O22121	Beta subunit of beta conglycinin (Fragment) OS	47.9	5.92
A0A0R4J5X9	Succinate dehydrogenase [ubiquinone] iron-sulfur subunit, mitochondrial OS	31.1	8.62
A0A0R0JZT7	Uncharacterized protein (Fragment) OS	23.4	5.43
C6TD56	Glyceraldehyde-3-phosphate dehydrogenase OS	37.0	7.47
C6TC98	Putative uncharacterized protein (Fragment) OS	35.9	5.63
I1N1W7	Uncharacterized protein OS	58.8	6.99
I1L2C2	Transmembrane 9 superfamily member OS	74.9	6.18
C6TKD5	Putative uncharacterized protein OS	52.1	8.81
I1LCN7	Coatomer subunit alpha OS	136.6	6.96
C6SZJ2	Uncharacterized protein OS	19.9	5.20
A0A0B2PWE7	Pyruvate dehydrogenase E1 component subunit alpha, mitochondrial OS	28.0	7.40
I1JTB9	Glucose-6-phosphate isomerase OS	53.6	6.14
A0A0R0HH51	Uncharacterized protein OS	29.4	7.21
I1LX52	Uncharacterized protein OS	32.3	9.13
I1MN17	Uncharacterized protein OS	16.6	9.14
K7KWC6	Uncharacterized protein OS	20.7	10.65
A0A0B2QVA3	RuBisCO large subunit-binding protein subunit beta, chloroplastic OS	58.1	5.24
I1NAI7	Uncharacterized protein OS	47.6	5.57
I1N5D5	Uncharacterized protein OS	59.0	5.96
A0A0B2RXX5	Pyruvate kinase OS	57.9	7.36
I1MP12	Uncharacterized protein OS	20.7	4.49
I1JR89	Uncharacterized protein OS	28.2	5.53
A0A0B2S1D9	UDP-arabinopyranose mutase 1 OS	40.1	5.92
A0A0R0L4C1	Uncharacterized protein OS	28.3	4.91
A0A0R0J9K8	Uncharacterized protein OS	29.5	9.28
I1LGG2	Glutathione peroxidase OS	18.5	6.19
A0A0B2QBU6	Transaldolase OS	35.5	4.98
A0A0B2QPA3	30S ribosomal protein 1, chloroplastic OS	26.7	5.48
K7L505	Uncharacterized protein OS	9.9	7.21

C6T172	Uncharacterized protein OS	26.7	9.01
C6F117	Putative ribosomal protein S15 OS	14.8	9.89
A0A0R0FER5	Elongation factor 1-alpha OS	49.4	9.06
A0A0B2PX53	Subtilisin inhibitor 1 OS	10.8	5.01
A0A0R0H296	Uncharacterized protein OS	14.2	10.51
I1LPI1	Uncharacterized protein OS	20.0	5.68
A0A0R0ETZ0	Uncharacterized protein (Fragment) OS	8.0	9.14
A0A0B2NX61	Biotin carboxylase 1, chloroplastic OS	58.9	7.50
C6TJA5	Putative uncharacterized protein OS	29.4	5.29
I1K302	Uncharacterized protein OS	13.1	5.38
I1J8A4	Thioredoxin reductase OS	39.7	8.40
A0A0B2P0X2	Glutathione S-transferase U18 OS	26.4	5.27
A0A0B2SEY7	Aspartic proteinase OS	55.4	6.73
I1L6T4	Uncharacterized protein OS	42.5	5.97
I1M669	Uncharacterized protein OS	38.9	6.25
I1K3R6	Uncharacterized protein OS	54.2	5.90
I1MC33	Uncharacterized protein OS	93.4	4.96
A0A0B2R290	Putative glutathione S-transferase OS	23.7	8.16
A0A0R0LAF9	Proteasome subunit alpha type OS	27.3	6.24
C6SWG6	Putative uncharacterized protein OS	14.1	8.76
I1M3S6	Uncharacterized protein OS	24.6	10.21
A0A0B2PVB1	Proteasome subunit alpha type (Fragment) OS	25.5	4.75
A0A0B2PW08	Protein transport protein SEC31 OS	119.0	5.19
A0A0B2QY95	Galactokinase OS	40.9	5.71
C6TB50	S-formylglutathione hydrolase OS	32.2	7.05
A0A0B2R0D6	Coatomer subunit gamma OS	98.7	5.19
C6T903	Uncharacterized protein OS	35.1	6.95
I1NJ85	Uncharacterized protein OS	67.4	6.98
I1LX29	Uncharacterized protein OS	19.8	9.95
A3KEY8	Glycinin A3B4 subunit OS	57.7	6.16
A0A0B2QVZ6	Putative methionine--tRNA ligase OS	41.9	6.29
A0A0R0EIF3	Uncharacterized protein OS	9.5	10.39
A0A0B2NUD4	Transmembrane protein 205 OS	43.0	8.22
C6THU0	Uncharacterized protein OS	35.0	5.50
I1L8N6	Uncharacterized protein OS	50.0	5.78
Q07CZ3	Glyceraldehyde-3-phosphate dehydrogenase OS	36.7	7.24
A0A0B2PIH3	Methionine aminotransferase OS	48.5	7.77
I1K1K4	Oleosin OS	19.1	8.29
I1LLS1	Uncharacterized protein OS	29.7	5.78
I1NH63	Polyadenylate-binding protein OS	71.2	8.60
A0A0B2PJB7	Bifunctional 3-dehydroquinate dehydratase/shikimate dehydrogenase, chloroplastic OS	57.3	7.05
I1NBI3	Uncharacterized protein OS	46.5	6.65

A0A0B2PSD2	Glycosyltransferase OS	50.3	5.76
I1NFD0	Uncharacterized protein OS	25.3	4.83
A0A0R0KHW0	Uncharacterized protein OS	14.8	10.90
C6TFC9	Putative uncharacterized protein (Fragment) OS	9.2	9.99
C6TEI8	Putative uncharacterized protein OS	14.7	7.47
C6SYC1	Uncharacterized protein OS	19.9	5.29
Q944T2	Translationally-controlled tumor protein homolog OS	19.0	4.73
I1JW13	Uncharacterized protein OS	30.4	8.35
K7L8B5	Uncharacterized protein OS	15.3	4.64
K7LA75	Uncharacterized protein OS	9.4	9.01
A0A0B2PPR3	60S acidic ribosomal protein P1-1 OS	8.7	4.16
A0A0B2S8F5	2-hydroxyacyl-CoA lyase OS	60.6	6.44
A0A0R0JTF7	Aspartate aminotransferase OS	47.5	8.28
Q9FQE4	Glutathione S-transferase GST 14 (Fragment) OS	25.3	7.59
A0A0B2PBS2	FAS-associated factor 2-B OS	35.4	5.41
I1MKY2	Uncharacterized protein OS	75.7	5.27
I1LL59	Protein disulfide-isomerase OS	64.8	4.79
I1LAJ3	Uncharacterized protein OS	36.6	7.74
A0A0B2NX08	T-complex protein 1 subunit gamma OS	60.2	6.33
A0A0B2QZM4	Synaptotagmin-7 OS	37.2	7.69
I1LE41	Uncharacterized protein OS	26.2	4.89
A0A0B2QM69	Aspartate aminotransferase OS	50.4	7.56
I1KL89	Uncharacterized protein OS	49.8	6.65
C6SZC5	Uncharacterized protein OS	12.3	9.31
I1NC19	Uncharacterized protein OS	48.8	8.32
A0A0R0IRH4	Phosphoglycerate kinase OS	42.4	6.73
I1LQ58	Dolichyl-diphosphooligosaccharide--protein glycosyltransferase 48 kDa subunit OS	47.6	6.42
C6TIQ8	Uncharacterized protein OS	47.9	7.58
I1LXQ1	Uncharacterized protein OS	79.5	7.75
I1MHH1	Uncharacterized protein OS	39.2	6.02
A0A0R0F3S5	Ribosomal protein OS	24.5	9.83
C6T441	Putative uncharacterized protein OS	12.7	7.24
C6TNV4	Uncharacterized protein OS	33.8	8.76
A0A0B2PK57	GTP-binding nuclear protein (Fragment) OS	25.0	6.86
B6EBD7	Heat shock protein 90-1 OS	80.4	5.02
I1J585	Uncharacterized protein OS	17.7	8.41
A0A0B2Q1U6	Uncharacterized protein OS	27.6	5.57
A0A0B2R210	Elongation factor 2 OS	94.0	6.11
I1LRM2	Uncharacterized protein OS	34.3	8.46
A0A0B2PKD9	10 kDa chaperonin OS	10.6	7.44
A0A0R0G869	Uncharacterized protein OS	37.9	5.59
P25273	Kunitz-type trypsin inhibitor KTI2 OS	22.8	6.55

B6EBD6	Heat shock protein 90-2 OS	80.1	5.07
C6TA02	Putative uncharacterized protein OS	27.7	9.77
A0A0B2SJ19	Uncharacterized protein OS	70.0	4.75
A0A0B2RKI5	Patellin-5 OS	35.2	8.76
A0A0B2RUX1	Secologanin synthase OS	57.1	9.39
Q3V5S6	Beta-conglycinin alpha subunit OS	70.2	5.15
A0A0B2R8L6	NADP-dependent alkenal double bond reductase P2 OS	37.9	6.32
I1N1Y6	40S ribosomal protein S8 OS	24.7	10.40
I1L5Z2	Aldose 1-epimerase OS	40.7	9.32
A0A0B2S7M0	Heat shock protein STI OS	65.6	6.73
A0A0B2QYK0	U-box domain-containing protein 72 OS	57.1	6.58
A0A0R0JQT2	Uncharacterized protein OS	44.5	5.43
A0A0R4J364	Uncharacterized protein OS	40.9	6.40
A0A0B2P7Y1	Ran-binding protein 1 like b OS	15.1	4.83
I1J7G5	Uncharacterized protein OS	18.6	5.02
I1K441	Uncharacterized protein OS	20.0	6.71
Q2VA66	Malonyltransferase OS	36.4	6.80
I1MU00	Uncharacterized protein OS	17.8	10.76
C6SXS7	Putative uncharacterized protein OS	21.7	9.50
Q39817	Calnexin homolog OS	62.0	4.89
A0A0B2NU60	Glutamate dehydrogenase OS	40.6	6.25
K7KM65	Uncharacterized protein OS	39.8	7.83
A0A0B2S5T6	Uncharacterized protein OS	20.9	9.06
C6TAY3	Uncharacterized protein OS	31.8	9.42
C6T9C2	Putative uncharacterized protein OS	34.6	6.19
I1KQB4	Uncharacterized protein OS	29.0	5.59
C6T4N0	Uncharacterized protein OS	21.9	4.55
K7L8K5	Uncharacterized protein OS	48.8	8.16
A0A0B2SQP1	Glyceraldehyde-3-phosphate dehydrogenase OS	43.2	8.03
C6SVA9	Acyl carrier protein OS	14.3	5.57
I1JYL3	Uncharacterized protein OS	33.9	7.55
I1N9E6	Elongation factor Tu OS	49.1	6.87
A0A0B2Q5I4	Putative AAA domain-containing protein C24B10.10c OS	95.3	6.48
I1K364	Uncharacterized protein OS	36.8	5.35
A0A0B2PSZ5	Glycinin G1 OS	55.8	6.28
C6SY71	Putative uncharacterized protein OS	16.8	10.81
A0A0R0FG80	Uncharacterized protein OS	23.9	6.55
I1MBR7	Uncharacterized protein OS	51.4	5.34
A0A0R0HJA5	Superoxide dismutase [Cu-Zn] OS	20.8	6.52
K7KB26	Uncharacterized protein OS	57.9	9.07
I1JIP5	Uncharacterized protein OS	60.2	6.58
C6TH76	Cysteine synthase OS	27.6	6.58
A0A0B2S409	26S protease regulatory subunit 8 like A OS	47.1	8.60

I1LM73	Uncharacterized protein OS	51.9	5.49
I1KJH7	Uncharacterized protein OS	20.8	7.58
I1LTG9	Uncharacterized protein OS	10.6	11.72
A0A0B2RNS9	LL-diaminopimelate aminotransferase, chloroplastic OS	20.8	5.31
C6TFP9	Non-specific lipid-transfer protein OS	12.2	8.78
I1KXG9	Uncharacterized protein OS	58.4	7.55
C6TKV3	Uncharacterized protein OS	46.1	5.96
Q71EW8	Methionine synthase OS	84.2	6.33
I1LJ68	Fructose-bisphosphate aldolase OS	42.9	8.16
C6TJG0	Uncharacterized protein OS	26.6	7.39
C6TFY7	Uncharacterized protein OS	10.6	8.40
A0A0B2QXP8	Eukaryotic translation initiation factor 3 subunit D OS	34.9	5.08
C6T211	Uncharacterized protein OS	9.3	7.80
I1KXQ3	Uncharacterized protein OS	29.8	7.68
A0A0R0HMU8	Uncharacterized protein OS	16.6	9.58
I1M7S3	Clathrin heavy chain OS	192.4	5.44
I1JG11	Uncharacterized protein OS	41.6	5.49
A0A0R0H183	Coatomer subunit alpha OS	129.0	6.81
C6TAA6	Uncharacterized protein OS	41.7	5.49
A0A0B2NYC0	60S ribosomal protein L8-3 OS	25.0	10.87
C6SV78	Ribosomal protein L15 OS	24.1	11.63
C6T187	Uncharacterized protein OS	8.1	4.88
A0A0B2QJJ0	Carbamoyl-phosphate synthase large chain OS	109.6	5.20
I1KSQ3	Uncharacterized protein OS	33.3	6.65
K7MF95	Alpha-mannosidase OS	107.2	6.38
I1LPX6	Fructose-bisphosphate aldolase OS	42.8	7.33
I1MJU7	Uncharacterized protein OS	73.7	5.34
A0A0B2RYD9	Branched-chain-amino-acid aminotransferase-like protein 3, chloroplastic OS	28.2	5.34
I1LS06	Uncharacterized protein OS	35.0	6.92
C6TMQ6	Uncharacterized protein OS	44.0	9.20
C6SVK2	Uncharacterized protein OS	13.5	9.45
A0A0R4J3N3	Uncharacterized protein OS	18.2	7.50
I1LDP2	Uncharacterized protein OS	27.9	8.92
I1JS70	Uncharacterized protein OS	42.2	5.63
A0A0R0EPL0	Uncharacterized protein OS	44.4	8.09
A0A0B2Q9C1	Uncharacterized protein OS	15.0	8.97
A0A0B2RHW2	Proteasome subunit beta type OS	29.2	6.14
A0A0B2RDY5	60S ribosomal protein L28-2 OS	16.9	11.05
A0A0B2R1B7	Quinone oxidoreductase-like protein, chloroplastic OS	33.9	6.33
I1M8L0	Uncharacterized protein OS	15.9	5.07
C6SVM3	Uncharacterized protein OS	14.0	9.47
Q9FQE8	Glutathione S-transferase OS	25.6	5.97

A0A0B2QAZ3	Fructose-bisphosphate aldolase OS	38.6	6.79
C6TDC8	Putative uncharacterized protein OS	24.4	6.33
I1JE09	Uncharacterized protein OS	17.7	10.20
A0A0R0F2K3	Uncharacterized protein OS	23.6	5.78
A0A0B2NR47	Ribonuclease UK114-like protein OS	14.2	5.22
I1JRI3	Uncharacterized protein OS	14.1	9.25
A0A0B2SMD0	Uncharacterized protein OS	14.7	5.35
C6TFE2	Uncharacterized protein OS	22.6	8.95
A0A0R0IHX7	Uncharacterized protein OS	22.2	9.14
C6TL29	Phosphoribulokinase OS	45.3	6.28
B2BF98	40S ribosomal protein S6 OS	28.0	10.71
I1MPU2	Uncharacterized protein OS	10.0	9.07
A0A0R0JND1	Signal peptidase I OS	11.7	8.22
A0A0B2QVK7	3-oxoacyl-[acyl-carrier-protein] reductase, chloroplastic OS	33.5	9.31
A0A0B2R5K2	Proteasome subunit beta type-2-A OS	22.5	6.29
C6TAU1	Putative uncharacterized protein OS	29.9	6.83
K7MBW4	Uncharacterized protein OS	12.6	8.50
A0A0B2SC27	Vacuolar-sorting receptor 1 OS	69.8	5.60
A0A0B2R2C7	C-1-tetrahydrofolate synthase, cytoplasmic OS	31.5	7.90
A0A0B2QMB6	Outer envelope pore protein 37, chloroplastic OS	28.4	7.28
K7MVK8	Uncharacterized protein OS	31.5	6.24
K7LP96	Uncharacterized protein OS	16.6	5.63
I1JFI7	Uncharacterized protein OS	14.3	5.92
A0A0R0HR30	Uncharacterized protein OS	47.0	5.62
A0A0B2QYV1	Actin-101 OS	41.6	5.49
A0A0R4J3P1	Glutamine synthetase OS	39.1	5.48
I1L1Q8	Uncharacterized protein OS	54.4	6.04
C6TBW0	Putative uncharacterized protein OS	23.3	7.66
I1LXK6	Uncharacterized protein OS	99.0	5.45
A0A0B2PXN3	Putative clathrin assembly protein OS	63.0	5.21
I1LUL9	Ketol-acid reductoisomerase OS	63.3	7.30
G3E7M9	Annexin OS	35.9	6.96
C6TAR9	Putative uncharacterized protein OS	34.0	7.03
I1LUH6	Uncharacterized protein OS	39.4	9.79
I1JFL5	Uncharacterized protein OS	14.7	6.52
C6SXC3	Uncharacterized protein OS	12.8	10.58
A0A0B2S9Q2	UTP--glucose-1-phosphate uridylyltransferase OS	51.2	5.33
A0A0B2QQ52	Ornithine carbamoyltransferase, chloroplastic OS	39.9	8.27
I1K672	Uncharacterized protein OS	96.3	4.78
A0A0R4J4D6	Uncharacterized protein OS	71.0	5.21
C6SVS6	Putative uncharacterized protein OS	17.4	9.52
I1JVU2	60S ribosomal protein L13 OS	23.9	10.93
I1JUY7	ATP-dependent 6-phosphofructokinase OS	56.0	6.80

A0A0B2SNJ3	Ubiquitin-activating enzyme E1 2 OS	113.5	5.27
A0A0R4J2U8	Uncharacterized protein OS	78.9	6.87
I1LDS8	Uncharacterized protein OS	25.9	5.11
I1KIE4	Uncharacterized protein OS	32.2	6.80
A0A0R0FSJ3	Uncharacterized protein OS	38.5	5.88
C6SZ11	Putative uncharacterized protein OS	27.0	6.92
I1JAU2	Uncharacterized protein OS	62.0	5.68
C6T374	Uncharacterized protein OS	15.4	5.69
A0A0B2QU31	Peptidyl-prolyl cis-trans isomerase FKBP62 OS	52.6	5.94
C6T522	Uncharacterized protein OS	10.0	6.57
A0A0B2RBC7	26S proteasome non-ATPase regulatory subunit 12 OS	50.3	7.62
I1JU43	Uncharacterized protein OS	30.7	9.47
I1MT11	Uncharacterized protein OS	86.8	7.59
C6THW5	Cytochrome b-c1 complex subunit Rieske, mitochondrial OS	29.2	8.44
A0A0R0GZ30	Ribosomal protein L37 OS	10.7	11.96
C6TBF6	Putative uncharacterized protein OS	19.5	7.27
I1JDD3	Uncharacterized protein OS	34.1	7.44
A0A0B2S257	Protein disulfide isomerase-like 2-3 OS	42.6	5.06
A0A0R0I4F6	Uncharacterized protein OS	31.8	7.36
I1LHX1	Uncharacterized protein OS	77.0	7.66
A0A0R0EJ71	Uncharacterized protein OS	45.1	8.19
A0A0B2RDX0	Eukaryotic initiation factor 4A-10 OS	46.6	5.55
A0A0B2P5G0	Polyadenylate-binding protein (Fragment) OS	66.1	6.86
A0A0R0JFL0	Uncharacterized protein OS	52.5	6.16
I1NFS4	ATP synthase subunit beta OS	59.8	6.15
I1L849	Uncharacterized protein OS	27.4	5.29
C6TKH0	Uncharacterized protein OS	31.6	6.84
Q01915	ATP synthase subunit alpha, mitochondrial OS	55.3	6.61
I1LR74	Lactoylglutathione lyase OS	21.0	5.64
A0A0R0GKM0	Xyloglucan endotransglucosylase/hydrolase OS	31.9	5.91
A0A0R0KV09	Uncharacterized protein OS	14.2	6.74
B8XJY3	Acyl-[acyl-carrier-protein] desaturase OS	44.9	6.33
A0A0B2RQ67	Argininosuccinate synthase, chloroplastic OS	44.0	5.96
A0A0R0KA84	Glyceraldehyde-3-phosphate dehydrogenase OS	36.8	7.58
A0A0R4J321	Proteasome subunit beta type OS	25.1	5.36
I1KCK6	Uncharacterized protein OS	49.7	5.57
A0A0B2SN35	T-complex protein 1 subunit alpha OS	59.1	6.51
C6TAX0	Putative uncharacterized protein OS	39.8	5.91
C6SV94	Uncharacterized protein OS	16.3	10.87
A0A0B2SNB7	Acidic endochitinase OS	16.6	6.04
A0A0R0F5Y8	Uncharacterized protein (Fragment) OS	32.5	7.05
K7MGG1	Uncharacterized protein OS	8.8	8.73
I1M5L8	Uncharacterized protein OS	94.9	5.21

K7KIK0	Uncharacterized protein OS	39.1	6.89
I1KIH0	Uncharacterized protein OS	90.3	6.38
Q8GUE3	Superoxide dismutase (Fragment) OS	15.4	6.54
A0A0B2PC35	60S ribosomal protein L34 OS	13.8	11.60
I1M322	Uncharacterized protein OS	46.7	6.95
I1K0Q3	Uncharacterized protein OS	21.9	4.93
K7K1R2	Uncharacterized protein OS	47.8	5.83
C6THI8	Putative uncharacterized protein OS	26.0	4.83
A0A0R0EJA3	Uncharacterized protein OS	48.5	4.64
A0A0B2RTD9	Prefoldin subunit 1 OS	14.0	5.34
C6SWY9	Uncharacterized protein OS	18.2	9.70
A0A0B2QSW5	26S protease regulatory subunit 6B like OS	36.3	6.14
D7EYG6	V-H(+)-ATPase subunit A OS	68.7	5.58
A0A0B2PHR2	Nascent polypeptide-associated complex subunit alpha-like protein OS	21.7	6.09
I1LUY8	Uncharacterized protein OS	21.9	5.62
A0A0B2STF7	Pyruvate kinase OS	57.6	6.90
C6SYU5	Putative uncharacterized protein OS	20.9	11.06
C6T4Q6	Putative uncharacterized protein OS	16.5	4.94
I1KZJ9	Uncharacterized protein OS	81.0	7.39
I1NJ59	DHAR class glutathione S-transferase OS	23.5	6.21
C6TCF1	Putative uncharacterized protein OS	27.8	5.40
A0A0B2P724	Glyoxysomal fatty acid beta-oxidation multifunctional protein MFP-a OS	78.3	8.92
A0A0R0IKB3	Uncharacterized protein (Fragment) OS	45.9	5.34
C6T960	Uncharacterized protein OS	32.0	5.92
A0A0B2R4X0	Putative UDP-arabinopyranose mutase 5 OS	38.5	5.95
A0A762	Calreticulin-1 OS	48.1	4.59
I1M261	Uncharacterized protein OS	56.7	5.85
C6TCX8	Putative uncharacterized protein OS	51.7	6.80
I1L7F3	Uncharacterized protein OS	15.4	10.46
Q39873	Lea protein OS	49.4	7.52
C6SZ18	Uncharacterized protein OS	7.5	11.17
A0A0R4J3C9	Serine hydroxymethyltransferase OS	51.7	7.31
C6T952	Putative uncharacterized protein (Fragment) OS	22.8	9.57
I1ML11	Uncharacterized protein OS	49.1	5.22
C6SYM8	Uncharacterized protein OS	21.9	5.12
I1JEL1	Uncharacterized protein OS	40.2	8.68
A0A0B2S0I2	6-phosphofructokinase 2 OS	43.7	6.99
I1MBN8	Uncharacterized protein OS	60.6	6.54
I1L417	Annexin OS	29.4	8.75
A0A0B2QSA3	Ubiquitin-40S ribosomal protein S27a OS	17.2	9.83
I1LR60	Uncharacterized protein OS	61.7	5.40

I1L932	Glycosyltransferase OS	54.4	5.40
A0A0B2RVW2	Thioredoxin M-type, chloroplastic OS	7.9	9.35
A0A0B2QIL8	EC protein like 2 OS	8.2	7.12
I1KZW8	Uncharacterized protein OS	33.6	5.31
C6SVR2	Uncharacterized protein OS	13.9	9.82
C6SYG6	Uncharacterized protein OS	21.8	4.84
A0A0B2QNW7	Protease 2 OS	78.7	5.57
I1LCM6	Uncharacterized protein OS	44.1	7.62
A0A0B2Q1F2	Aldose 1-epimerase OS	40.6	9.32
A0A0R0I1W6	Uncharacterized protein OS	40.5	7.01
K7KZX8	Uncharacterized protein OS	20.8	6.10
I1JP95	S-(hydroxymethyl)glutathione dehydrogenase OS	40.3	6.76
Q9XES7	Seed maturation protein PM27 OS	38.0	9.38
I1JF86	Uncharacterized protein OS	57.8	6.60
C6SXE1	Cyanate hydratase OS	18.7	7.49
Q9XER5	Seed maturation protein PM22 OS	16.7	5.36
I1NIY6	Uncharacterized protein OS	51.4	6.43
I1KKQ2	Uncharacterized protein OS	43.9	8.87
A0A0R4J5B9	Uncharacterized protein OS	21.1	8.65
A0A0B2NVB9	Chaperonin CPN60-2, mitochondrial OS	61.2	5.90
I1KP14	Uncharacterized protein OS	108.7	6.48
I1MR37	Uncharacterized protein OS	55.4	6.95
A0A0R4J2M8	Proteasome subunit alpha type OS	27.1	7.49
C6T0B5	Uncharacterized protein OS	14.0	6.15
C6SY43	Uncharacterized protein OS	21.3	6.23
A0A0R0G3Z6	Uncharacterized protein OS	38.3	5.94
C6TFE6	Putative uncharacterized protein OS	13.7	9.58
A0A0B2RJ36	Glutathione reductase, chloroplastic OS	56.1	8.35
K7MGT1	Uncharacterized protein OS	38.2	4.93
A0A0B2NW29	Phospholipase D OS	91.6	5.74
C6TAN4	Uncharacterized protein OS	50.9	8.68
A0A0R0HDX9	Uncharacterized protein OS	39.9	6.87
Q9XGS2	Ni-binding urease accessory protein UreG OS	30.1	6.39
A0A0R0H9T5	Peptidyl-prolyl cis-trans isomerase OS	18.1	7.83
A0A0B2RHP8	Membrane steroid-binding protein 2 OS	22.7	4.84
I1N7G4	Uncharacterized protein OS	60.8	6.80
A0A0R0FXB3	Uncharacterized protein OS	46.7	6.68
I1LMP4	Eukaryotic translation initiation factor 3 subunit F OS	31.5	5.34
A0A0B2Q7X0	Starch synthase, chloroplastic/amyloplastic OS	68.0	7.06
A0A0R0F8Z5	Xylose isomerase OS	53.9	6.80
A0A0R0GIN2	Uncharacterized protein OS	27.5	7.33
I1KGU0	40S ribosomal protein S12 OS	15.1	5.77
I1K0G0	Uncharacterized protein OS	43.1	4.97

I1KGH6	Uncharacterized protein OS	54.4	6.27
C6ZS00	Disease resistance protein/LRR protein-related protein OS	53.8	8.44
K7MP24	Uncharacterized protein OS	93.1	5.00
I1LC97	Uncharacterized protein OS	39.6	5.39
Q8W1A4	Uncoupling protein 1a (Fragment) OS	25.7	9.61
I1L860	Uncharacterized protein OS	58.0	6.49
A0A0B2RF20	Aspartyl aminopeptidase OS	45.1	7.31
K7LSQ5	Uncharacterized protein OS	41.5	8.54
C6T142	Small ubiquitin-related modifier OS	11.2	5.10
I1KRJ7	Uncharacterized protein OS	24.4	6.92
Q42806	Pyruvate kinase, cytosolic isozyme OS	55.3	7.56
I1MQ30	Uncharacterized protein OS	39.2	6.74
I1MT10	Uncharacterized protein OS	70.8	5.39
Q7G1G6	Aspartate aminotransferase OS	45.6	7.88
I1LPJ9	Uncharacterized protein OS	77.5	5.49
I1K3M8	Uncharacterized protein OS	13.6	6.34
A0A0B2S8M0	Putative fructokinase-4 OS	41.0	5.49
A0A0B2S398	Alpha-L-arabinofuranosidase 1 OS	73.8	6.11
A0A0B2SGU5	Sucrose synthase OS	89.0	6.38
I1JBI5	Uncharacterized protein OS	44.0	6.33
I1KLL8	Uncharacterized protein OS	32.0	9.19
I1ND62	Uncharacterized protein OS	7.6	4.68
C6T9Q3	Putative uncharacterized protein OS	17.2	10.29
A0A0B2STB1	Lysine--tRNA ligase (Fragment) OS	67.5	6.55
C6TAQ9	Uncharacterized protein OS	28.5	6.55
Q02243	Wound-induced protein (Fragment) OS	11.1	7.71
K7LSL8	Uncharacterized protein OS	50.4	8.24
A0A0B2Q5Y2	Phosphoribosylformylglycinamidine cyclo-ligase OS	31.6	4.89
A0A0R0IK03	Uncharacterized protein OS	42.0	5.38
Q948P5	Ferritin-4, chloroplastic OS	27.5	5.96
C6TFD7	Putative uncharacterized protein OS	24.1	5.03
I1ND14	Pyrophosphate--fructose 6-phosphate 1-phosphotransferase subunit alpha OS	67.6	6.96
I1KQE8	Uncharacterized protein OS	109.5	5.36
A0A0B2QAS8	Glycine-rich RNA-binding protein 2, mitochondrial OS	16.0	9.99
I1K0D3	Uncharacterized protein OS	42.4	8.69
I1JNK9	Uncharacterized protein OS	41.6	5.49
I1LFD6	DHAR class glutathione S-transferase OS	23.4	6.43
K7L746	Uncharacterized protein OS	35.4	8.54
I1M2K9	Uncharacterized protein OS	72.4	5.87
K7KFI7	Uncharacterized protein OS	13.4	10.01
A0A0B2NWM9	Luminal-binding protein 4 OS	73.6	5.21
A0A0B2RFT2	Carbamoyl-phosphate synthase large chain OS	106.7	5.19

C6TDD3	Putative uncharacterized protein OS	23.3	8.16
I1MT35	Uncharacterized protein OS	53.1	7.15
I1LFL5	Uncharacterized protein OS	127.2	5.76
A0A0B2QKV0	Sucrose synthase OS	91.5	6.29
I1KNE0	Uncharacterized protein OS	29.8	5.40
A0A0B2RAW9	60S ribosomal protein L6 OS	25.8	10.14
A0A0B2PFW0	LETM1 and EF-hand domain-containing protein 1, mitochondrial OS	68.7	5.43
K7L382	Uncharacterized protein OS	28.7	5.17
C6T8B0	Putative uncharacterized protein OS	47.7	6.16
A0A0B2RK27	Alanine--tRNA ligase (Fragment) OS	101.0	5.99
I1K6M7	Uncharacterized protein OS	26.0	8.03
I1K7A2	Dolichyl-diphosphooligosaccharide--protein glycosyltransferase subunit 1 OS	57.4	7.58
A0A0R0HLE9	Uncharacterized protein OS	50.6	6.55
Q9M507	Beta-ketoacyl-ACP synthetase I-2 OS	49.7	7.71
A0A0B2PH15	Putative glutathione S-transferase OS	8.0	4.94
I1LM95	Uncharacterized protein OS	24.6	9.74
I1L903	Isocitrate dehydrogenase [NAD] subunit, mitochondrial OS	39.4	7.49
A0A0B2PUQ5	Brain protein 44-like protein OS	11.0	9.42
K7LDT9	Uncharacterized protein OS	103.3	6.76
A0A0B2PM77	Tocopherol cyclase, chloroplastic OS	23.2	9.32
A0A0R0KQC7	Carboxypeptidase OS	44.9	6.19
B0M1A5	Betaine aldehyde dehydrogenase OS	54.6	5.49
I1LNE6	Uncharacterized protein OS	59.2	5.85
I1K7F4	Uncharacterized protein OS	60.8	9.35
P01070	Trypsin inhibitor A OS	24.0	5.11
A0A0R0HQX5	Beta-galactosidase OS	84.8	6.64
I1MGR4	Uncharacterized protein OS	80.6	7.49
I1JV69	Uncharacterized protein OS	37.6	5.59
I1NJ15	Uncharacterized protein OS	39.8	4.98
A0A0B2RFW5	Omega-amidase NIT2 OS	23.5	6.52
A0A0B2P4D2	Glycinin G3 OS	54.1	5.97
I1KTK0	Uncharacterized protein OS	25.9	10.08
A0A0B2QSP2	T-complex protein 1 subunit gamma OS	60.2	6.49
I1MJL6	Uncharacterized protein OS	25.9	10.15
A0A0B2RF11	Cell division cycle protein 48 like OS	90.5	5.26
I1LKF8	Uncharacterized protein OS	18.2	4.41
R9ZV72	Ribosomal protein S11 (Fragment) OS	14.5	12.10
C6SYI8	Putative uncharacterized protein OS	18.4	6.34
A0A0R0JI98	Uncharacterized protein OS	25.0	4.56
A0A0B2PKN2	Uncharacterized protein OS	15.9	7.58
A0A0B2RMW6	Elongation factor 1-delta OS	14.3	4.91

I1MZF9	Uncharacterized protein OS	81.2	6.62
Q9SWB2	Seed maturation protein PM41 OS	8.2	4.97
A0A0B2PNC3	Putative 26S proteasome non-ATPase regulatory subunit 3 OS	55.5	8.81
A0A0B2PRX8	Elongation factor Tu, chloroplastic OS	32.8	5.10
A0A0B2SL14	Serine hydroxymethyltransferase OS	57.3	7.77
I1J4G9	Uncharacterized protein OS	55.1	6.15
A0A0B2Q1C1	Uncharacterized protein OS	14.6	5.12
Q945U3	Acyl-coenzyme A oxidase OS	74.5	8.10
C6SWL4	Uncharacterized protein OS	20.4	10.23
A0A0R0GZF0	Annexin OS	35.7	7.31
I1KAB7	Protein disulfide-isomerase OS	56.1	4.98
A0A0R4J455	Uricase OS	35.1	8.29
I1LNM2	NADH dehydrogenase subunit 9 OS	22.6	7.88
I1J637	Uncharacterized protein OS	41.7	5.78
A0A0B2PTB2	20 kDa chaperonin, chloroplastic OS	14.8	8.78
A0A0B2R6C6	Heat shock protein 83 OS	88.4	4.97
I1L8R1	Uncharacterized protein OS	44.7	10.45
C6TIR2	Putative uncharacterized protein OS	16.9	4.27
I1LVH0	Uncharacterized protein OS	40.2	7.46
A0A0B2PPT7	Glutamine--tRNA ligase OS	90.1	6.33
I1M9F8	Aspartate aminotransferase OS	40.3	7.15
A0A0B2QLB8	Peroxisomal fatty acid beta-oxidation multifunctional protein AIM1 OS	78.1	9.23
K7LMI8	Uncharacterized protein OS	41.6	6.54
A0A0R0JTW3	Uncharacterized protein OS	27.8	9.60
C6T078	Uncharacterized protein OS	14.3	10.83
I1N5X6	Uncharacterized protein OS	15.7	7.28
A0A0B2NUS6	GEM-like protein 5 OS	29.9	8.38
C6T1L0	Putative uncharacterized protein OS	22.1	9.88
A0A0B2Q3Q6	Exportin-2 OS	96.5	5.36
I1LV54	Uncharacterized protein OS	46.6	6.80
C6SVH2	Putative uncharacterized protein OS	20.7	4.67
A0A0R0EAR0	Uncharacterized protein OS	18.2	12.02
A0A0R4J5B7	40S ribosomal protein SA OS	34.1	5.26
I1L7P0	ATP-dependent Clp protease proteolytic subunit OS	32.9	7.99
C6SYJ4	Uncharacterized protein OS	16.5	10.71
C6TMA8	Uncharacterized protein OS	12.2	4.86
I1J7H3	Ferritin OS	28.7	6.18
A0A0B2NYV2	Triosephosphate isomerase, chloroplastic OS	33.1	6.79
A0A0R0EG69	Uncharacterized protein OS	27.4	7.14
A0A0B2PT30	40S ribosomal protein S6 OS	28.1	10.71
A0A0B2SIC4	Putative rhamnose biosynthetic enzyme 1 OS	39.5	6.54
A0A0B2R4N3	30S ribosomal protein 2, chloroplastic OS	15.9	7.24

I1JRR6	Uncharacterized protein OS	27.2	7.59
I1M3C2	Uncharacterized protein OS	33.7	9.23
A0A0B2RLP6	Cytochrome c oxidase subunit 6b-1 OS	19.5	4.39
I1M2Y6	Uncharacterized protein OS	37.8	5.82
I1KEY6	Uncharacterized protein OS	97.3	5.19
A0A0R0H5B2	Uncharacterized protein OS	35.0	5.80
I1LH18	Uncharacterized protein OS	48.0	5.49
I1KG52	Uncharacterized protein OS	79.0	5.43
A0A0R0IF33	Uncharacterized protein OS	61.6	4.94
I1LEC3	Uncharacterized protein OS	49.9	6.92
K7LAT3	Uncharacterized protein OS	26.3	9.61
C6SWQ6	Uncharacterized protein OS	26.9	9.52
C6SYZ8	Uncharacterized protein OS	23.6	10.32
A0A0B2SAE3	Uncharacterized protein OS	24.3	9.07
Q39871	Late embryongenesis abundant protein OS	50.6	6.67
A0A0R0J4X0	Uncharacterized protein OS	29.0	4.75
I1JGR5	Uncharacterized protein OS	71.6	5.31
I1M1B6	Acyl carrier protein OS	14.9	5.31
C6T0R5	Uncharacterized protein OS	21.0	11.18
A0A0B2SW26	Phosphoenolpyruvate carboxylase, housekeeping isozyme OS	110.6	6.05
A0A0B2RNC8	50S ribosomal protein L3-1, chloroplastic OS	28.8	10.45
A0A0R0GHQ9	Sulfurtransferase OS	39.1	8.07
Q39801	51 kDa seed maturation protein OS	51.0	7.11
A0A0B2PYZ1	Importin subunit alpha OS	58.1	5.31
K7LRE0	Uncharacterized protein OS	10.3	5.21
A0A0B2PJN4	Isocitrate dehydrogenase [NAD] regulatory subunit 1, mitochondrial OS	38.5	7.96
I1N3Z3	Uncharacterized protein OS	34.0	6.05
A0A0B2PJR1	31 kDa ribonucleoprotein, chloroplastic OS	31.9	4.83
A0A0R4J439	Uncharacterized protein OS	49.3	6.21
A0A0B2SIP2	Peptidyl-prolyl cis-trans isomerase OS	16.0	6.79
I1L0S5	Uncharacterized protein OS	19.0	6.93
I1KB94	Alpha-galactosidase OS	46.1	6.29
I1MTP0	Plasma membrane ATPase OS	105.0	6.73
C6T699	Uncharacterized protein OS	16.0	4.92
A0A0B2PTP2	Kunitz-type trypsin inhibitor KTI1 OS	25.8	8.54
I1LXY1	Uncharacterized protein OS	89.0	6.77
C6SY82	Eukaryotic translation initiation factor 5A OS	17.3	6.20
I1NA39	Lambda class glutathione S-transferase OS	27.0	5.71
I1JXQ5	Alpha-mannosidase OS	114.2	6.70
A0A0R0EWQ1	Uncharacterized protein OS	92.9	5.76
K7KD02	Uncharacterized protein OS	53.3	6.80
I1MX58	Uncharacterized protein OS	60.2	6.40

I1KQ93	Uncharacterized protein OS	63.4	5.53
A0A0R0FKM0	Uncharacterized protein (Fragment) OS	16.2	8.48
A0A0B2R0Z0	1-Cys peroxiredoxin OS	24.3	6.55
K7L1Z5	Uncharacterized protein OS	28.6	5.83
A0A0B2QV05	Dolichyl-diphosphooligosaccharide--protein glycosyltransferase subunit STT3 OS	44.4	8.98
A0A0B2PG19	Xylulose kinase OS	61.0	6.11
I1M005	Uncharacterized protein OS	41.9	7.59
A0A0R0IWL2	Proliferating cell nuclear antigen OS	22.5	5.43
C6SVZ7	Putative uncharacterized protein OS	24.7	6.07
C6SY42	Putative uncharacterized protein OS	24.3	4.70
I1J7M1	Non-specific lipid-transfer protein OS	12.2	9.50
A0A0B2RB73	DNA damage-binding protein 1 OS	17.2	4.54
A0A0B2PR66	Malate dehydrogenase OS	30.7	6.24
I1N0T6	Uncharacterized protein OS	51.2	6.93
I1JFX0	Uncharacterized protein OS	20.7	7.17
A0A0B2QA24	Asparagine--tRNA ligase, cytoplasmic 1 OS	63.4	6.35
I1K198	Uncharacterized protein OS	17.4	9.33
C6TBY4	Putative uncharacterized protein OS	14.1	9.45
A0A0B2QPZ1	Eukaryotic peptide chain release factor subunit 1-3 OS	48.9	5.50
I1MDY5	Uncharacterized protein OS	57.2	6.25
A0A0B2QRD1	26S protease regulatory subunit S10B like B OS	44.5	7.83
I1KDB2	Uncharacterized protein OS	47.3	6.54
A0A0R0GGB7	Uncharacterized protein OS	96.5	5.55
Q948X9	Beta-conglycinin alpha-subunit OS	72.4	5.40
A0A0B2PIM9	40S ribosomal protein S20-1 OS	13.8	9.58
A0A0R0IQ05	Uncharacterized protein OS	16.7	5.77
I1NGG4	Uncharacterized protein OS	26.0	5.02
C6TJ33	Putative uncharacterized protein OS	33.3	5.02
A0A0B2QKN1	60S ribosomal protein L18-3 (Fragment) OS	20.9	11.17
B0M197	Peroxisomal voltage-dependent anion-selective channel protein OS	29.8	8.65
C6T7D9	Putative uncharacterized protein OS	47.5	6.35
C6T7Y1	Uncharacterized protein OS	41.7	5.49
I1KEN4	Uncharacterized protein OS	110.2	5.74
A0A0B2QVF8	Protein transport protein Sec23A OS	64.1	6.05
C6SXY7	Putative uncharacterized protein OS	18.3	10.20
I1MBN4	Uncharacterized protein OS	22.7	8.65
A0A0R0HVZ9	Uncharacterized protein OS	21.5	9.32
A0A0R0HH38	Uncharacterized protein OS	15.5	4.46
I1JR27	Uncharacterized protein OS	98.7	8.46
C6T5S2	Putative uncharacterized protein OS	16.6	10.71
A0A0R4J4C8	ATP synthase subunit beta OS	59.8	6.15

C6TH90	ATP-dependent (S)-NAD(P)H-hydrate dehydratase OS	39.6	8.02
A0A0B2SI43	Uncharacterized protein OS	15.6	6.29
I1JYI8	Uncharacterized protein OS	11.1	4.32
I1J582	Eukaryotic translation initiation factor 3 subunit I OS	35.8	7.34
I1M5M4	Uncharacterized protein OS	102.9	5.22
A0A0B2SKC6	NHP2-like protein 1 OS	13.9	7.12
I1JYG8	Uncharacterized protein OS	14.8	5.15
A0A0B2RAA1	GDP-mannose 3,5-epimerase 1 OS	40.0	6.54
A0A0R0FXY0	Uncharacterized protein OS	55.1	5.44
I1J5B4	Uncharacterized protein OS	82.3	7.46
A0A0R0K416	Uncharacterized protein OS	18.1	6.87
A0A0B2NVQ0	50S ribosomal protein L5, chloroplastic OS	29.0	10.07
A0A0B2PP75	Cysteine proteinase inhibitor OS	27.6	7.80
A0A0B2QMY5	Histidine--tRNA ligase OS	72.7	7.34
I1KZW7	Pyrophosphate--fructose 6-phosphate 1-phosphotransferase subunit beta OS	61.4	7.03
I1KNH5	Pyrophosphate--fructose 6-phosphate 1-phosphotransferase subunit beta OS	59.9	6.98
C6TAZ2	Putative uncharacterized protein OS	23.2	7.42
A0A0R0J0Z8	Uncharacterized protein OS	35.2	4.78
A0A0R0JAM3	Uncharacterized protein OS	35.4	8.76
A0A0B2P9F0	Polyadenylate-binding protein OS	70.7	7.75
I1KID4	Uncharacterized protein OS	21.6	8.43
Q9SWB6	Probable bifunctional TENA-E protein OS	26.0	5.35
C6TAC5	Putative uncharacterized protein OS	21.4	5.54
A0A0R0ICH7	Uncharacterized protein OS	54.8	6.34
A0A0R4J681	Uncharacterized protein OS	31.8	7.44
A0A0R4J3L5	Proteasome subunit alpha type OS	27.4	5.86
A0A0B2S2M9	T-complex protein 1 subunit beta OS	54.7	5.80
A0A0B2P3I1	60S ribosomal protein L9 OS	21.2	9.32
A0A0B2SKU1	Peroxisomal fatty acid beta-oxidation multifunctional protein AIM1 OS	78.1	9.25
C6SV97	Peptidyl-prolyl cis-trans isomerase OS	21.9	9.17
A0A0R0H2D1	Uncharacterized protein OS	21.3	11.52
A0A0R0FHN7	Uncharacterized protein OS	9.5	8.88
A0A0B2PQW4	Pistil-specific extensin-like protein OS	30.2	9.63
A0A0B2RE21	Threonine synthase, chloroplastic OS	26.5	7.01
I1N3A6	Alpha-galactosidase OS	45.7	7.20
I1JDI7	Uncharacterized protein OS	85.4	6.39
C6SXB4	Uncharacterized protein OS	15.6	10.78
C6TGR3	Uncharacterized protein OS	25.5	9.01
A0A0R0L3E1	Uncharacterized protein OS	59.1	8.18
A0A0B2RC62	2,3-bisphosphoglycerate-independent phosphoglycerate	60.9	5.69

	mutase OS		
A0A0R0FND9	Uncharacterized protein OS	26.6	7.81
I1MPM4	Uncharacterized protein OS	146.4	6.09
A0A0R0LHX3	Elongation factor Ts OS	114.2	4.83
A0A0B2SC46	Snakin-1 OS	9.9	8.29
A0A0B2S5G5	3-isopropylmalate dehydratase OS	58.0	7.72
C6TFY9	Putative uncharacterized protein (Fragment) OS	19.8	9.70
C6TBI4	Uncharacterized protein OS	12.8	10.61
I1JMQ3	Uncharacterized protein OS	39.1	6.02
A0A0R0JFW9	Uncharacterized protein OS	22.7	8.66
I1NH37	Uncharacterized protein OS	44.4	10.15
K7K5E6	Uncharacterized protein OS	15.8	9.26
C6SVH9	Uncharacterized protein OS	8.8	9.25
A0A0R4J4L3	Annexin OS	35.9	7.91
A0A0R0JPF7	Uncharacterized protein (Fragment) OS	22.1	6.35
A0A0R0G3Y8	4-hydroxy-4-methyl-2-oxoglutarate aldolase OS	17.7	6.32
A0A0B2PLS8	Clathrin light chain 1 OS	23.6	9.11
K7L2F1	Uncharacterized protein OS	20.9	9.31
K7LQ70	Poly [ADP-ribose] polymerase OS	88.9	5.38
C6T3L6	Putative uncharacterized protein OS	19.3	4.93
C6TID4	Uncharacterized protein OS	34.5	6.24
I1LI96	Phosphoserine aminotransferase OS	45.0	7.94
A0A0R0HT24	Uncharacterized protein OS	22.5	5.99
C6TIB6	40S ribosomal protein S3a OS	29.6	9.85
C6SWS6	Uncharacterized protein OS	23.2	5.74
A0A0B2PR64	Ras-related protein RABD2a OS	22.4	5.27
C6TAB7	Uncharacterized protein OS	40.5	5.78
I1K565	Uncharacterized protein OS	108.5	6.79
I1MJT8	Uncharacterized protein OS	16.2	5.15
K7MQU7	Uncharacterized protein OS	25.9	9.39
I1MB51	Uncharacterized protein OS	19.9	6.29
I1KYU6	Alpha-1,4 glucan phosphorylase OS	95.8	6.43
A0A0R4J495	Uncharacterized protein OS	24.9	6.54
C6SWF2	Putative uncharacterized protein OS	19.8	6.92
C6T2Y2	Uncharacterized protein OS	18.6	5.01
A0A0B2SVU5	Alpha-amylase OS	43.5	4.48
I1N7E6	Uncharacterized protein OS	47.7	5.97
A0A0B2SRM4	Transmembrane emp24 domain-containing protein 2 OS	24.5	6.34
I1KZT2	Lactoylglutathione lyase OS	32.4	6.01
I1MNU1	Uncharacterized protein OS	58.4	5.47
K7LPV5	Uncharacterized protein OS	107.7	8.66
K7LWI4	ATP synthase subunit alpha OS	55.7	5.21
I1LS33	Uncharacterized protein OS	34.6	5.21

C6TLM5	Putative uncharacterized protein OS	27.7	8.46
I1KSR7	Uncharacterized protein OS	33.4	5.27
C6T5A5	Ubiquitin-fold modifier-conjugating enzyme 1 OS	19.7	7.81
A0A0B2RZX4	40S ribosomal protein S4 OS	30.0	10.32
C6T9Y3	Putative uncharacterized protein OS	45.0	4.94
I1J8H1	Aconitate hydratase OS	98.5	5.86
C6T670	Uncharacterized protein OS	16.6	10.71
A0A0R0HCG7	Uncharacterized protein OS	484.4	6.16
A0A0B2SHS2	3-oxoacyl-[acyl-carrier-protein] reductase, chloroplastic OS	28.3	5.83
K7L817	40S ribosomal protein S12 OS	12.8	6.29
I1M0K3	Cysteine proteinase inhibitor OS	27.7	7.08
A0A0B2RME3	Polyadenylate-binding protein RBP45 OS	36.7	6.79
A0A0R0KWB3	Uncharacterized protein OS	29.6	10.18
A0A0B2S5M2	Vicilin-like antimicrobial peptides 2-2 OS	70.9	5.26
A0A0B2RV05	60S ribosomal protein L6 OS	25.8	10.18
I1LE33	Uncharacterized protein OS	68.2	6.32
P93164	Gamma-glutamyl hydrolase OS	37.7	6.54
A0A0B2R9G7	Neutral alpha-glucosidase AB OS	101.0	6.32
A7VJA5	T-complex protein 1 subunit delta OS	57.6	7.31
A0A0R4J2M7	40S ribosomal protein S4 OS	29.9	10.21
A0A0R0F0E0	Uncharacterized protein OS	17.4	8.31
I1KPY5	Lactoylglutathione lyase OS	39.6	7.03
A0A0R4J653	Uncharacterized protein OS	80.0	6.47
C6TKK2	Putative uncharacterized protein OS	27.0	6.52
F2XF89	TPR1 OS	30.5	5.03
C6T0W7	Uncharacterized protein OS	18.4	4.82
I1M138	Uncharacterized protein OS	51.2	8.00
C6SW56	Uncharacterized protein OS	14.3	10.81
A0A0B2STW7	60S ribosomal protein L32-1 OS	15.6	10.71
A0A0B2R397	40S ribosomal protein S27 OS	9.6	8.73
C6TA47	Uncharacterized protein OS	12.5	5.25
A0A0B2PYX7	Uncharacterized protein OS	21.6	4.89
A0A0B2RM47	Pectinesterase OS	54.4	7.56
A0A0B2R054	Putative lipid-transfer protein DIR1 OS	9.9	7.90
I1LG74	Uncharacterized protein OS	42.7	7.28
A0A0B2RSG4	Putative carboxylesterase 2 OS	31.8	6.16
A0A0B2QNB8	Uncharacterized protein OS	14.7	7.90
K7K9D0	Uncharacterized protein OS	107.4	6.00
C6T176	Putative uncharacterized protein OS	18.1	9.03
I1K6B1	Uncharacterized protein OS	139.4	4.94
A0A0B2RLQ7	Protein disulfide-isomerase OS	57.2	5.06
I1LQ43	Adenylyl cyclase-associated protein OS	50.6	7.03
A0A0B2QHL7	40S ribosomal protein S7 OS	22.0	9.77

K7KWZ7	Uncharacterized protein OS	30.8	6.30
I1KDR3	Uncharacterized protein OS	48.8	5.62
I1JQD9	Eukaryotic translation initiation factor 3 subunit C OS	104.6	5.63
C6T529	Ribulose bisphosphate carboxylase small chain OS	19.7	8.53
A0A0B2QPH2	Caffeic acid 3-O-methyltransferase OS	38.1	5.96
I1JPP3	Uncharacterized protein OS	89.6	5.26
C6SZI6	Putative uncharacterized protein OS	21.2	6.06
C6SVE3	Putative uncharacterized protein OS	10.0	9.11
C6TFI8	Uncharacterized protein OS	16.7	11.02
A0A0B2Q0G3	Outer plastidial membrane protein porin OS	29.8	9.06
C6TL67	Putative uncharacterized protein OS	34.9	8.41
K7KQ85	Uncharacterized protein OS	7.9	9.94
A0A0B2QST3	Eukaryotic translation initiation factor 3 subunit J OS	25.7	4.98
I1LFV5	Uncharacterized protein OS	41.5	6.58
A0A0B2PM17	31 kDa ribonucleoprotein, chloroplastic OS	32.8	4.70
I1NGJ0	40S ribosomal protein S3a OS	26.8	10.15
K7KBY9	Uncharacterized protein OS	32.3	4.89
I1KJJ6	Uncharacterized protein OS	19.2	6.76
I1J9Q7	Glutamate dehydrogenase OS	44.8	6.40
I1M290	Uncharacterized protein OS	16.7	10.92
A0A0B2QHD9	Glucose-6-phosphate/phosphate translocator 2, chloroplastic OS	31.0	9.51
A0A0B2SS77	60S ribosomal protein L27 OS	15.1	10.42
A0A0R0IGQ4	Uncharacterized protein (Fragment) OS	17.6	8.82
I1JMZ7	Uncharacterized protein OS	111.5	6.10
I1M395	Uncharacterized protein OS	8.3	7.84
A0A0B2SJP8	Eukaryotic translation initiation factor 3 subunit G OS	31.7	8.21
A0A0B2RKC3	Ribosomal protein OS	34.7	8.97
I1L4U3	Uncharacterized protein OS	88.4	6.30
A0A0B2P491	Histone H4 OS	11.4	11.33
A0A0R0JWC4	Uncharacterized protein OS	42.7	5.25
I1N065	Uncharacterized protein OS	114.8	5.58
A0A0R0GX77	Uncharacterized protein OS	25.9	5.00
I1JBG1	Uncharacterized protein OS	35.4	8.31
C6SX05	Uncharacterized protein OS	21.2	6.37
I1JL98	Uncharacterized protein OS	18.5	5.38
A0A0R0HE95	Uncharacterized protein OS	118.5	5.80
K7KEI1	Uncharacterized protein OS	125.0	5.82
A0A0R0F259	Uncharacterized protein OS	65.2	6.21
I1MB25	Serine hydroxymethyltransferase OS	47.0	8.28
K7N0I2	Aminoacylase-1 OS	49.7	6.90
A0A0R0JFI6	Endoglucanase OS	65.7	8.72
A0A0B2R1G8	Phosphoribosylaminoimidazole-succinocarboxamide synthase,	45.4	6.28

	chloroplastic OS		
I1K7E6	Uncharacterized protein OS	9.4	5.20
A0A0R0JRK3	Uncharacterized protein OS	23.0	5.05
A0A0R4J467	Uncharacterized protein OS	27.1	7.65
I1LS94	Acyl-coenzyme A oxidase OS	75.9	8.53
A0A0B2PXR6	Chitotriosidase-1 OS	28.5	7.97
A0A0R0FZI9	Uncharacterized protein OS	96.0	6.46
C6T9M6	Reticulon-like protein OS	24.2	8.81
A0A0R0EH01	Uncharacterized protein OS	48.1	7.42
I1LCV0	Uncharacterized protein OS	14.1	9.79
A0A0B2RD88	rRNA 2'-O-methyltransferase fibrillarin 2 OS	22.3	10.30
K7KYR9	Uncharacterized protein OS	26.8	7.93
K7MVE6	Uncharacterized protein OS	39.8	9.39
I1K6H2	Uncharacterized protein OS	104.2	6.38
A0A0B2SEB7	Ras-related protein RABH1b OS	22.9	7.90
A0A0B2PRG4	26S proteasome non-ATPase regulatory subunit 11 OS	46.8	5.81
A0A0B2SS66	DEAD-box ATP-dependent RNA helicase 2 OS	38.4	6.55
A0A0B2NUM3	Uncharacterized protein OS	59.7	7.62
I1N0Y1	Uncharacterized protein OS	62.9	6.04
A0A0B2Q4Q7	Pyrophosphate-energized vacuolar membrane proton pump OS	76.6	5.33
I1LD09	Uncharacterized protein OS	58.1	6.30
Q38JD2	Temperature-induced lipocalin OS	21.4	7.24
I1M1N6	Uncharacterized protein OS	34.6	6.71
I1LJ14	Uncharacterized protein OS	81.5	9.04
A0A0R0K7M8	Uncharacterized protein OS	10.2	5.49
A0A0R4J2Q7	Uncharacterized protein OS	25.0	10.35
Q9FQ95	In2-1 protein OS	27.0	5.35
A0A0B2QYA8	Dynein light chain 2, cytoplasmic OS	12.9	6.57
A0A0B2RTU6	Villin-4 OS	106.2	6.02
C6SVE0	Eukaryotic translation initiation factor 5A OS	17.4	5.76
I1K7D2	Catalase OS	54.9	7.03
A0A0B2SGW3	ABC transporter C family member 4 OS	136.3	7.96
A7XZJ7	Profilin OS	14.1	4.84
I1N5R7	Uncharacterized protein OS	34.7	6.25
P50346	60S acidic ribosomal protein P0 OS	34.1	5.25
A0A0B2NWA6	Dihydroflavonol-4-reductase OS	32.3	6.11
C6TF48	Putative uncharacterized protein OS	14.3	7.28
A0A0B2NZI1	T-complex protein 1 subunit theta OS	58.6	5.40
A0A0R0LCF1	Uncharacterized protein OS	63.7	5.71
I1KG03	Methylenetetrahydrofolate reductase OS	51.9	6.93
A0A097PSS0	AT4G29520-like protein (Fragment) OS	19.1	5.03
A0A0B2QVA0	22.7 kDa class IV heat shock protein OS	22.3	6.11
K7LN99	Uncharacterized protein OS	52.6	8.56

A0A0R0F220	Uncharacterized protein OS	11.6	8.31
I1KQV6	Uncharacterized protein OS	52.0	5.52
C6T8J1	Uncharacterized protein OS	36.5	6.20
K7MZ77	Uncharacterized protein OS	11.2	4.41
I1LF60	Uncharacterized protein OS	54.2	5.02
A6XKY2	Chalcone-flavonone isomerase family protein OS	23.2	6.80
C6TIS4	Putative uncharacterized protein OS	19.1	9.41
I1JY81	Uncharacterized protein OS	41.2	8.41
C6SXG4	Uncharacterized protein OS	15.2	7.20
A0A0B2Q5P0	Uncharacterized protein OS	32.9	5.17
I1LAA7	Uncharacterized protein OS	107.0	5.82
C6T8I8	Putative uncharacterized protein OS	20.8	4.73
K7LRJ2	Uncharacterized protein OS	8.0	8.95
I1L005	Uncharacterized protein OS	32.2	9.28
A0A0R0KP24	Uncharacterized protein OS	82.4	6.49
I1K5D3	Eukaryotic translation initiation factor 3 subunit C OS	104.2	5.78
C6T0D7	Putative uncharacterized protein OS	17.3	6.07
A0A0B2PQW5	Diphosphomevalonate decarboxylase OS	46.4	6.05
A0A0B2S539	IAA-amino acid hydrolase ILR1-like 1 OS	14.3	8.48
A0A0B2RX37	50S ribosomal protein L14 OS	13.4	10.32
A0A0B2PE63	Histone-lysine N-methyltransferase SUVR2 OS	91.5	6.09
A0A0B2Q6V4	Subtilisin-like protease OS	80.0	8.78
A0A0R0H8N9	Uncharacterized protein OS	16.8	10.01
C6TJH0	Putative uncharacterized protein OS	25.3	6.11
I1LPL2	Uncharacterized protein OS	29.4	10.20
A0A0B2R220	60S ribosomal protein L11 OS	19.4	9.91
P51061	Phosphoenolpyruvate carboxylase OS	110.7	6.47
A0A0B2SKW7	Uncharacterized protein OS	29.6	4.75
I1MX61	Glutathione peroxidase OS	25.6	9.19
A0A0B2NU93	E3 SUMO-protein ligase RanBP2 OS	48.0	4.91
I1NHK2	Uncharacterized protein OS	19.4	9.92
I1N8B5	Uncharacterized protein OS	76.0	5.47
I1N6A5	Alpha-1,4 glucan phosphorylase OS	110.3	5.47
I1JUQ4	Uncharacterized protein OS	49.5	9.16
A0A0R0ELE0	Pyrroline-5-carboxylate reductase OS	28.5	8.32
A5JVZ7	Superoxide dismutase OS	26.7	8.65
A0A0B2RK83	Cysteine synthase OS	37.1	5.82
K7M3Y6	Uncharacterized protein OS	45.9	6.58
A0A0R0IBE3	Uncharacterized protein OS	10.1	5.19
A0A0R0HKE3	Uncharacterized protein OS	57.8	5.86
C6T7C4	Putative uncharacterized protein (Fragment) OS	26.0	5.83
C6TFC7	Putative uncharacterized protein OS	22.3	9.60
C6TCU1	Putative uncharacterized protein OS	29.6	4.88

C6T097	Putative uncharacterized protein OS	8.1	9.19
C6T048	Uncharacterized protein OS	17.5	10.56
I1LDX0	Uncharacterized protein OS	68.4	6.42
K7M8W8	Uncharacterized protein OS	23.4	5.90
C6T611	Putative uncharacterized protein (Fragment) OS	19.7	4.83
A0A0B2NSC6	40S ribosomal protein S5 OS	22.5	9.60
C6T7K0	Uncharacterized protein OS	27.8	5.00
C6T5T0	40S ribosomal protein S3a OS	26.0	10.18
I1L0Q8	Uncharacterized protein OS	49.0	6.84
A0A0B2Q254	Malate synthase OS	62.7	6.28
C6TJB7	Uncharacterized protein OS	23.8	5.82
I1L530	Uncharacterized protein OS	40.7	5.57
I1M6B5	Glucose-1-phosphate adenylyltransferase OS	55.2	6.48
C6T0L2	Protein SLE3 OS	11.1	6.80
A0A0B2S1T0	Non-specific lipid-transfer protein-like protein OS	24.9	7.66
I1JPD6	Eukaryotic translation initiation factor 3 subunit A OS	87.9	8.66
I1LSW3	Uncharacterized protein OS	50.1	5.73
I1LAL4	Uncharacterized protein OS	61.1	6.24
A0A0B2PRA9	Putative hydrolase YtaP OS	40.7	5.67
K7KI86	Uncharacterized protein OS	98.5	5.64
A0A0B2PTZ4	Fasciclin-like arabinogalactan protein 1 OS	38.1	9.36
A0A0B2PJX3	Ubiquinone biosynthesis protein COQ9, mitochondrial OS	33.8	6.38
A0A0B2PHR4	Anthocyanin 5-aromatic acyltransferase OS	31.9	5.78
A0A0R0J475	Uncharacterized protein OS	79.2	7.69
A0A0B2QFG2	Delta-1-pyrroline-5-carboxylate dehydrogenase 12A1, mitochondrial OS	61.3	7.02
I1JS92	Uncharacterized protein OS	18.0	8.09
I1MEH7	Uncharacterized protein OS	27.9	5.92
I1LJE6	Uncharacterized protein OS	20.2	5.44
Q9XES8	Seed maturation protein PM28 OS	9.5	4.81
A0A0R0IZU0	Uncharacterized protein OS	37.1	5.47
D3Y5N8	Glycosyltransferase OS	49.2	6.76
A0A0R0FZN4	Uncharacterized protein OS	34.9	6.19
A0A0B2RE62	Protein TOC75-3, chloroplastic OS	72.8	7.21
A0A0B2RG07	Calnexin like OS	61.9	4.87
C6T0E8	60S ribosomal protein L18a OS	21.3	10.43
A0A0B2SX74	Succinate-semialdehyde dehydrogenase, mitochondrial OS	58.9	8.00
A0A0R0LEX1	Uncharacterized protein OS	30.7	6.47
A0A0R0F8L2	Uncharacterized protein (Fragment) OS	26.4	5.40
I1LZ03	Uncharacterized protein OS	44.6	10.48
A0A0B2RJ86	Serine/threonine-protein phosphatase 2A 65 kDa regulatory subunit A beta isoform OS	57.8	5.24
A0A0B2Q7E0	Carboxylesterase 1 OS	23.5	7.12

I1LFG6	Uncharacterized protein OS	67.6	7.50
C6T588	Uncharacterized protein OS	16.8	4.88
A0A0R0L499	Uncharacterized protein (Fragment) OS	100.7	5.05
A0A0B2PEV2	Putative aldo-keto reductase 2 OS	37.8	6.55
A0A0B2PZX9	Aldose 1-epimerase OS	40.7	9.36
C6T968	40S ribosomal protein S8 OS	24.7	10.42
A0A0B2Q3D7	Putative carboxylesterase 7 OS	35.7	5.68
I1LEI7	Uncharacterized protein OS	39.3	6.34
C6TMB1	Cytochrome c oxidase subunit 2 OS	42.3	6.84
C6TDP9	Putative uncharacterized protein OS	32.7	5.52
K7K4G2	Uncharacterized protein OS	71.7	5.49
A0A0B5E2H5	DHAR class glutathione S-transferase OS	28.9	8.35
C6T571	Histone H2B OS	13.1	10.21
C6TMV8	Histone H2A OS	15.6	10.67
F8SMB9	Adenosylhomocysteinase OS	53.2	6.01
I1KSB7	Uncharacterized protein OS	53.0	5.16
I1JVL6	Uncharacterized protein OS	19.6	9.95
A0A0B2PR21	Alpha-glucosidase 2 OS	90.6	5.60
A0A0B2R887	Peroxygenase OS	26.6	6.61
A0A0B2RNW9	Putative 26S proteasome non-ATPase regulatory subunit 7 OS	34.8	6.28
A0A0B2S6P2	D-3-phosphoglycerate dehydrogenase, chloroplastic OS	45.9	5.69
I1MUH0	Uncharacterized protein OS	16.7	9.17
A0A0B2S970	Aminomethyltransferase OS	44.1	8.75
I1KH72	Acetyltransferase component of pyruvate dehydrogenase complex OS	59.3	7.91
A0A0B2Q383	2,3-bisphosphoglycerate-independent phosphoglycerate mutase 1 OS	52.9	6.15
A0A0B2RYN3	Uncharacterized protein OS	15.2	5.60
A0A0B2RWZ9	Histone H2B.2 OS	6.8	6.52
I1KVC1	Uncharacterized protein OS	26.0	7.83
A0A0B2NQ45	Alpha-N-acetylglucosaminidase OS	92.3	7.25
A0A0B2SX31	Alpha-1,4 glucan phosphorylase OS	110.4	5.47
K7LVG9	Uncharacterized protein OS	20.8	9.52
I1JPZ4	Uncharacterized protein OS	11.3	4.37
C6SYP2	Putative uncharacterized protein OS	11.1	4.41
I1NF81	Uncharacterized protein OS	99.6	5.10
I1NAI0	Uncharacterized protein OS	16.2	5.00
A0A0R0GV71	Uncharacterized protein OS	14.1	10.68
I1N783	Proteasome subunit alpha type OS	27.3	5.87
I1NJ48	Uncharacterized protein OS	45.9	7.50
I1N2Z5	Protein SLE1 OS	12.2	5.38
I1MG55	Uncharacterized protein OS	41.6	6.79
I1LVC1	Uncharacterized protein OS	14.9	5.88

C6SVK7	Putative uncharacterized protein OS	15.9	6.38
I1LNS9	Uncharacterized protein OS	39.2	9.82
C6TLZ2	Putative uncharacterized protein OS	6.1	6.55
C6T751	Eukaryotic translation initiation factor 3 subunit E OS	37.9	6.54
A0A0R0FPQ1	Uncharacterized protein OS	12.1	7.24
A0A0B2RRR1	Fructose-bisphosphate aldolase OS	40.9	8.21
A0A0B2RSM4	Chitinase domain-containing protein 1 OS	40.1	7.99
A0A0B2S3X0	Oligopeptidase A OS	87.9	6.30
A0A0R0EBC1	Uncharacterized protein OS	23.7	8.46
A0A0B2SN99	5-oxoprolinase OS	137.7	6.15
A0A0B2QYJ4	Eukaryotic translation initiation factor 3 subunit B OS	82.6	5.31
A0A0B2SG07	Cysteine synthase OS	31.7	6.39
Q2LAK4	Cytochrome P450 monooxygenase CYP74A2 (Fragment) OS	22.8	5.86
C6SXM8	Ribosomal protein L19 OS	24.6	11.44
A0A0B2P0S6	Seed maturation protein PM36 OS	21.5	5.66
A0A0R4J4G9	Uncharacterized protein OS	23.7	6.98
Q9M508	Beta-ketoacyl-ACP synthetase I OS	49.7	7.49
I1KYF2	Uncharacterized protein OS	83.7	7.20
I1NA97	Uncharacterized protein OS	30.8	5.68
A0A0B2R8V3	Translation initiation factor eIF-2B subunit delta OS	56.4	9.35
C6T3R4	60S ribosomal protein L27 OS	15.6	10.42
A0A0B2SIM2	RING-box protein 1a OS	12.6	6.65
I1KCA8	Elongation factor Tu OS	52.1	6.68
K7K9M8	Uncharacterized protein OS	10.4	8.81
K7MSK8	Uncharacterized protein OS	5.4	9.47
A0A0B2RRQ5	Golgin candidate 6 OS	101.1	4.82
I1KRI7	60S ribosomal protein L27 OS	15.7	10.42
I1KB79	Chalcone-flavonone isomerase family protein OS	20.0	5.00
I1MZ13	Clathrin heavy chain OS	192.8	5.53
O81413	Ferric leghemoglobin reductase-2 OS	53.0	7.34
A0A0B2SQB3	LysM domain-containing GPI-anchored protein 1 OS	22.1	6.87
A0A0R0GP29	Uncharacterized protein OS	64.7	7.06
C6TBA9	40S ribosomal protein S8 OS	24.8	10.36
A0A0B2P3L2	Methylcrotonoyl-CoA carboxylase subunit alpha, mitochondrial OS	77.3	7.11
K7L7I8	Uncharacterized protein OS	117.9	6.14
K7MMU8	Uncharacterized protein OS	43.6	9.41
A0A0B2NUS2	Putative N-acetyl-gamma-glutamyl-phosphate reductase, chloroplastic OS	42.9	7.17
A0A0B2Q286	Arginase OS	31.4	6.25
I1LDE9	Uncharacterized protein OS	44.4	10.17
K7KWH3	Uncharacterized protein OS	8.8	4.72
I1LWI3	Uncharacterized protein OS	31.5	5.24

I1K3J0	Signal recognition particle subunit SRP72 OS	73.1	8.91
I1N0P0	Uncharacterized protein OS	12.8	9.03
A0A0R0F5I3	Uncharacterized protein (Fragment) OS	23.2	7.40
I1M540	Uncharacterized protein OS	39.4	7.52
A0A0B2RH95	Heat shock cognate protein 80 OS	80.1	5.03
A0A0B2S8C8	Uncharacterized protein OS	12.3	4.97
A0A0B2S5J5	Putative receptor protein kinase TMK1 OS	99.0	6.68
I1JRP8	Uncharacterized protein OS	38.1	7.15
A0A0B2QVI3	UDP-glucose 4-epimerase GEPI48 OS	37.9	7.71
C6SZP9	Putative uncharacterized protein (Fragment) OS	17.4	9.31
A0A0B2RHM2	Putative aldo-keto reductase 1 OS	24.6	8.43
A0A0B2QV10	65-kDa microtubule-associated protein 1 OS	65.3	5.29
Q1JU56	Kunitz trypsin inhibitor OS	24.1	5.21
A0A0B2QEK8	Eukaryotic translation initiation factor 3 subunit L OS	42.9	5.72
A0A0B2SL81	Proteasome subunit alpha type OS	25.6	5.68
A0A0B2SSB2	26.5 kDa heat shock protein, mitochondrial OS	25.6	7.49
K7K8E5	Uncharacterized protein OS	57.7	4.78
C6TJM8	Alpha-galactosidase OS	43.6	6.37
I1L7J2	Uncharacterized protein OS	13.8	9.60
C6TLM9	Putative uncharacterized protein OS	30.9	5.15
Q42447	Maturation protein OS	25.6	6.52
I1M736	Uncharacterized protein OS	13.0	8.85
A0A0B2RZM9	ADP,ATP carrier protein 1, mitochondrial OS	42.1	9.70
A0A0R0EJU2	Uncharacterized protein OS	158.5	6.77
I1JC08	Uncharacterized protein OS	81.8	7.21
I1KS98	Uncharacterized protein OS	46.7	6.33
A0A0B2PZX4	Cystathionine beta-lyase, chloroplastic OS	50.5	6.16
I1J7C8	Uncharacterized protein OS	42.9	6.76
A0A0R0K7S9	Uncharacterized protein OS	16.5	4.45
A0A0B2PI12	60S ribosomal protein L13a-1 OS	23.7	10.27
I1MNR1	Uncharacterized protein OS	49.0	5.52
I1N334	Uncharacterized protein OS	36.3	6.74
A0A0R0I461	Uncharacterized protein OS	38.8	6.10
C6TCC4	Putative uncharacterized protein OS	17.5	10.40
B2YDR0	Gly m Bd 28K allergen (Fragment) OS	50.7	6.19
A7LNF7	Sucrose-phosphate synthase (Fragment) OS	29.8	6.09
Q9SWB4	Poly [ADP-ribose] polymerase 3 OS	91.6	5.59
C6T6C3	Putative uncharacterized protein OS	7.5	5.81
A0A0R0H8Q8	Uncharacterized protein OS	8.0	9.88
A0A0B2RB28	1-Cys peroxiredoxin OS	24.4	7.39
I1KJI7	Nucleoside diphosphate kinase OS	16.5	6.80
I1JRF2	Uncharacterized protein OS	62.7	7.09
I1KS58	Uncharacterized protein OS	53.8	6.09

C6SZQ3	Uncharacterized protein OS	17.2	10.21
Q6VEV1	40S ribosomal protein S25 (Fragment) OS	10.5	10.76
C6T828	Putative uncharacterized protein OS	32.5	5.55
C6THH9	Putative uncharacterized protein (Fragment) OS	29.8	11.37
O49154	Ferredoxin-dependent glutamate synthase (Fragment) OS	110.1	6.40
A0A0B2SHV7	Aldo-keto reductase family 4 member C9 OS	34.7	6.89
I1MK74	Uncharacterized protein OS	58.3	6.58
C6TER7	Putative uncharacterized protein OS	32.6	7.12
I1JGG0	Uncharacterized protein OS	13.4	5.40
I1LFZ1	Uncharacterized protein OS	16.5	4.82
A0A0B2PSB8	Tryptophan--tRNA ligase, cytoplasmic OS	46.2	6.30
K7LP83	Uncharacterized protein OS	55.5	5.49
K7MSH4	Uncharacterized protein OS	43.5	8.69
A0A0B2QTF3	Putative prefoldin subunit 5 OS	16.9	6.79
A0A097PK35	Pollen Ole e 1 allergen and extensin family protein (Fragment) OS	13.2	5.38
I1KUF8	Uncharacterized protein OS	10.7	10.43
K7LHL1	Uncharacterized protein OS	11.0	9.19
I1MP01	Glycosyltransferase OS	53.8	5.90
A0A0B2QX92	Aspartic proteinase OS	56.0	6.71
I1LIP7	Uncharacterized protein OS	33.7	5.14
A0A0B2P106	N-alpha-acetyltransferase 16, NatA auxiliary subunit OS	103.1	7.02
I1N520	Uncharacterized protein OS	35.1	6.55
C6SVB8	40S ribosomal protein S24 OS	15.7	10.64
P25973	Cystatin (Fragment) OS	5.3	7.25
I1LT24	Uncharacterized protein OS	28.0	6.68
I1M361	Uncharacterized protein OS	12.2	5.45
A0A0B2S647	Proteasome subunit alpha type OS	27.4	7.09
A0A0R0E916	Uncharacterized protein OS	32.8	5.53
A0A0B2Q2D9	Putative pre-mRNA-splicing factor ATP-dependent RNA helicase OS	82.0	7.37
A0A0B2PNU6	Heat shock protein 83 OS	80.2	5.08
A0A0B2NZT4	Blue copper protein OS	19.9	6.29
C6TI51	Uncharacterized protein OS	34.1	5.43
C6TES6	Putative uncharacterized protein OS	40.6	7.06
A0A0R0IMB6	Uncharacterized protein (Fragment) OS	31.1	6.43
I1MCF0	Uncharacterized protein OS	45.2	6.43
A0A0B2Q5D1	Extended synaptotagmin-3 OS	62.3	5.74
K7LRH3	Uncharacterized protein OS	23.3	10.48
C6T9R8	Glyceraldehyde-3-phosphate dehydrogenase OS	48.1	7.43
A0A0B2S7G7	Cullin-associated NEDD8-dissociated protein 1 OS	134.0	6.01
C6T0C6	Putative uncharacterized protein OS	18.6	6.80
K7MVA6	Uncharacterized protein OS	24.3	6.79

A0A0R0I6Z6	Uncharacterized protein OS	170.0	5.11
A0A0R0FCP4	Uncharacterized protein OS	26.1	6.39
C6SXY4	Uncharacterized protein OS	18.7	5.19
A0A0B2SLI8	Nucleoside diphosphate kinase OS	16.5	6.80
A0A0R0HM17	Uncharacterized protein OS	17.5	4.65
A0A0R4J387	Chalcone-flavonone isomerase family protein OS	23.5	5.00
A0A0R0EM21	Uncharacterized protein OS	50.6	7.49
A0A0B2R3J3	Ribosomal protein L19 OS	23.7	11.21
C6T2C1	Putative uncharacterized protein OS	17.1	7.80
P29756	Catalase-1/2 OS	56.8	7.27
K7L8T2	Uncharacterized protein OS	257.6	7.27
G8D4B3	Ribulose bisphosphate carboxylase large chain (Fragment) OS	22.4	6.05
I1LJB4	Uncharacterized protein OS	11.7	10.68
I1ND90	Carboxypeptidase OS	43.9	7.18
I1N5S0	Uncharacterized protein OS	42.8	6.77
A0A0R0KIG7	Uncharacterized protein (Fragment) OS	53.1	8.24
A0A0B2RGY3	Uncharacterized protein OS	30.5	9.42
A0A0B2PET6	Phospholipase A-2-activating protein (Fragment) OS	61.2	5.34
I1L8Q0	Lambda class glutathione S-transferase OS	27.1	5.52
A0A0B2QW44	60S ribosomal protein L10 OS	25.0	10.24
I1JZP0	Uncharacterized protein OS	43.6	6.92
A0A0R0IG99	Profilin OS	8.5	5.26
D2DKF0	Dihydroxyacid dehydratase OS	64.0	6.23
Q2LAJ8	Cytochrome P450 monooxygenase CYP76E3 (Fragment) OS	39.7	5.03
A0A0R0HU58	Uncharacterized protein (Fragment) OS	35.8	6.89
C6T3U6	Ribosomal protein L19 OS	24.8	11.44
K7N3S3	60S ribosomal protein L36 OS	10.2	10.84
A0A0B2RKL6	N-carbamoylputrescine amidase OS	33.5	6.09
C6TKM8	Putative uncharacterized protein OS	25.0	9.85
A0A0B2P0E5	Non-lysosomal glucosylceramidase OS	94.5	5.99
I1KN68	Glycosyltransferase OS	53.2	6.90
C6T530	Uncharacterized protein OS	22.0	9.80
C6TA16	Alpha-SNAP protein OS	32.8	5.50
I1LM67	Uncharacterized protein OS	21.1	5.59
A0A0B2Q155	Disease resistance response protein 206 OS	20.6	5.39
A0A0B2Q268	2-oxoglutarate dehydrogenase, mitochondrial OS	116.0	6.86
A0A0B2PP86	Trypsin inhibitor A OS	23.3	6.09
I1MQT7	Uncharacterized protein OS	134.4	5.96
I1JC44	Uncharacterized protein OS	46.5	5.88
C6TFW4	Uncharacterized protein OS	16.7	5.05
I1NAH4	Uncharacterized protein OS	17.6	10.14
I1KM70	Uncharacterized protein OS	19.3	5.06
A0A0B2R5J6	Callose synthase 9 OS	69.8	9.69

C6T801	Putative uncharacterized protein (Fragment) OS	24.6	8.53
A0A0B2QKF8	Glucose and ribitol dehydrogenase OS	31.7	7.08
I1JTW0	Uncharacterized protein OS	23.5	9.44
C6TMV9	Uncharacterized protein OS	31.4	8.22
A0A0B2SCM2	Quinone oxidoreductase-like protein 2 like (Fragment) OS	27.3	9.11
P62163	Calmodulin-2 OS	16.8	4.27
A0A0R0KK84	Uncharacterized protein OS	54.6	5.38
Q71LY8	3-phosphoshikimate 1-carboxyvinyltransferase OS	47.6	5.29
A0A0B2R341	WD repeat-containing protein 26 OS	60.6	6.80
I1JHT7	Uncharacterized protein OS	20.8	5.03
A0A0B2PMR9	Em-like protein GEA6 OS	11.5	5.60
K7MZ42	Uncharacterized protein OS	176.0	6.05
Q70EM0	Dehydrin OS	23.8	6.47
A0A0R4J581	40S ribosomal protein S4 OS	30.0	10.26
A0A0R0FZ23	Uncharacterized protein OS	16.3	11.02
P04776	Glycinin G1 OS	55.7	6.23
I1KC70	Glyceraldehyde-3-phosphate dehydrogenase OS	36.7	7.24
C6T0A7	Uncharacterized protein OS	17.5	10.56
C6SZ55	Uncharacterized protein OS	10.6	8.40
I1JB54	Uncharacterized protein OS	11.9	9.38
I1MFH9	Lactoylglutathione lyase OS	32.4	6.01
A0A0R4J4X6	Uncharacterized protein OS	18.5	8.34
O22120	Alpha subunit of beta conglycinin (Fragment) OS	63.1	4.96
A0A0B2QUB7	Protein P21 OS	10.4	7.96
C6SXG1	Uncharacterized protein OS	17.5	6.37
C6SYA5	60S ribosomal protein L18a OS	21.3	10.42
Q7M210	Glycinin A3B4 (Plasmid pSPGL1) (Fragment) OS	27.1	5.38
C6SVR8	Uncharacterized protein OS	16.0	10.24
I1K6J6	Glutathione peroxidase OS	18.4	7.08
Q4U3W3	Beta-amylase OS	56.1	5.55
I1M988	Uncharacterized protein OS	8.7	9.32
C6T531	Putative uncharacterized protein OS	20.9	11.06
I1NBV9	Uncharacterized protein OS	21.0	11.06
A0A0B2RTV1	Copper transport protein ATOX1 OS	8.5	6.60
C6SVR5	Putative uncharacterized protein OS	23.9	5.97
C6T5K7	Putative uncharacterized protein (Fragment) OS	28.2	10.99
A0A0B2NVH5	40S ribosomal protein S8 OS	24.8	10.40
C6T5E0	Uncharacterized protein OS	15.6	10.71
C6SXP1	Putative uncharacterized protein OS	25.1	4.60
I1MT23	Uncharacterized protein OS	59.8	7.14
C6T0U3	Uncharacterized protein OS	13.8	9.58
I1KVR2	Uncharacterized protein OS	28.3	9.95

I1KLN6	Uncharacterized protein OS	59.7	7.62
A0A0B2RDV2	60S ribosomal protein L28-2 OS	16.9	11.03
C6TGZ7	Uncharacterized protein OS	29.8	7.68
C6SXS9	Putative uncharacterized protein OS	23.5	6.21
K7KCC7	Uncharacterized protein OS	9.2	5.05
C6T4I7	Ribosomal protein L19 OS	24.6	11.44
C6SX86	Uncharacterized protein OS	16.4	10.68
C6T3W3	Uncharacterized protein OS	16.4	10.59
I1LN92	Uncharacterized protein OS	20.4	10.23
C6TFH5	Superoxide dismutase [Cu-Zn] OS	13.7	6.37
A0A0B2S7M5	Putative calcium-binding protein CML7 OS	11.9	7.18
C6SVX5	Uncharacterized protein OS	25.1	10.35
A0A0B2QFY9	Triosephosphate isomerase, chloroplastic OS	33.1	6.79
I1JHP9	Uncharacterized protein OS	22.7	8.66
C6TLT3	40S ribosomal protein S3a OS	29.7	9.80
A0A0B2P3R8	Cytochrome b-c1 complex subunit 9 OS	8.1	9.60
A0A0B2NYC2	Malate dehydrogenase (Fragment) OS	27.2	6.58
C6SW88	Putative uncharacterized protein OS	22.5	9.39
C6SWA5	Uncharacterized protein OS	14.9	10.90
I1LG76	Uncharacterized protein OS	39.1	8.02
D6C500	HSP90-2 OS	80.1	5.06
I1MJE1	Uncharacterized protein OS	52.8	6.32
A0A0R4J4A9	Uncharacterized protein OS	23.0	7.90
A0A0B2S237	RuBisCO large subunit-binding protein subunit alpha, chloroplastic OS	61.7	5.34
I1LP45	Uncharacterized protein OS	39.3	5.73
I1K135	Glyceraldehyde-3-phosphate dehydrogenase OS	36.4	8.09
I1KZK2	Uncharacterized protein OS	58.9	5.85
I1MYX8	Alpha-SNAP protein OS	32.7	5.17
A0A0B2RX23	Peroxiredoxin-2F, mitochondrial OS	21.2	8.63
C6T9G0	Uncharacterized protein OS	22.6	5.71
I1K5M9	Uncharacterized protein OS	63.5	5.53
I1KAK6	Uncharacterized protein OS	27.4	6.42
I1JSJ0	Uncharacterized protein OS	10.4	10.83
I1LK56	Uncharacterized protein OS	21.2	8.25
A0A0B2QPS9	Quinone oxidoreductase-like protein, chloroplastic OS	34.7	6.25
A0A0B2QDF3	Cell division cycle protein 48 like OS	90.9	5.24
I1N0B4	Uncharacterized protein OS	5.6	10.24
A0A0B2RGT1	Transmembrane protein 205 OS	38.2	9.38
A0A0B2SC72	26S proteasome non-ATPase regulatory subunit 4 OS	33.0	4.92
A0A0R4J5N3	Uncharacterized protein OS	46.8	5.68
A0A0B2QZH4	Formate--tetrahydrofolate ligase OS	67.4	7.12
I1JS67	Uncharacterized protein OS	42.3	8.00

I1K3X9	Uncharacterized protein OS	49.6	5.14
A0A0R4J5B2	Uncharacterized protein OS	14.8	5.38
A0A0B2PLI8	Auxin-repressed 12.5 kDa protein OS	12.9	9.76
C6SVH8	Putative uncharacterized protein OS	12.1	7.12
I1K7H1	Uncharacterized protein OS	45.1	8.73
C6TF75	Putative uncharacterized protein OS	9.6	5.25
I1L432	Glutamine synthetase OS	36.7	5.92
A0A0B2QWF5	T-complex protein 1 subunit epsilon OS	47.6	6.19
I1M3R5	Uncharacterized protein OS	39.6	9.85
C6TIW0	Fructose-bisphosphate aldolase OS	42.5	8.56
A0A0B2S4Z7	30S ribosomal protein S4, chloroplastic OS	13.2	9.45
A0A0R0GW88	Uncharacterized protein OS	42.7	4.79
I1MGE7	Uncharacterized protein OS	65.9	7.87
Q2PMR3	50S ribosomal protein L33, chloroplastic OS	7.6	9.88
A0A0B2P9C1	Uncharacterized protein OS	6.2	5.34
K7MA66	40S ribosomal protein S6 OS	28.2	10.70
I1N628	Uncharacterized protein OS	29.7	9.07
A0A0B2PCY4	Alpha-1,4 glucan phosphorylase OS	104.1	5.29
I1MUN1	Uncharacterized protein OS	25.6	7.42
C6TGW1	Reticulon-like protein OS	28.5	8.94
A0A0B2SW33	40S ribosomal protein SA OS	31.8	5.34
C6THM7	Serine hydroxymethyltransferase OS	48.3	7.91
A0A0R0GE46	Uncharacterized protein OS	24.1	6.84
K7L2Q6	Uncharacterized protein OS	13.3	9.51
A0A0R0IIS7	Uncharacterized protein OS	45.4	7.99
A0A0B2QQ54	UDP-glucose 4-epimerase OS	39.1	7.77
I1K7J4	Uncharacterized protein OS	50.4	4.87
I1KJK0	Uncharacterized protein OS	39.3	6.19
A0A0B2RH39	Nuclease domain-containing protein 1 OS	108.9	6.49
K7L353	Uncharacterized protein OS	15.0	10.17
I1KSE0	Uncharacterized protein OS	27.9	5.08
A0A0B2R5H4	Universal stress protein A-like protein OS	19.9	6.29
A0A0R0I9M4	Annexin OS	35.5	8.76
I1LTL9	Uncharacterized protein OS	89.7	5.31
K7MJ55	Uncharacterized protein OS	7.3	8.65
I1M9F7	Aspartate aminotransferase OS	50.6	7.90
A0A0R0FCD2	Uncharacterized protein OS	48.7	9.04
I1LHP6	Uncharacterized protein OS	81.7	5.34
C6SVD6	Uncharacterized protein OS	8.8	8.76
A0A0R4J309	Uncharacterized protein OS	18.6	10.51
K7LIC8	Uncharacterized protein OS	6.1	9.09
C6TBN2	Probable aldo-keto reductase 1 OS	38.2	6.57
I1JT00	Uncharacterized protein OS	55.6	8.81

A0A0B2RBH2	Uncharacterized protein OS	7.8	9.94
C6TG59	Putative uncharacterized protein OS	49.7	5.68
A0A0B2RGI1	Cysteine synthase OS	34.4	5.63
K7MUJ9	Uncharacterized protein OS	48.6	5.10
A0A0B2SSF8	Secologanin synthase OS	59.9	8.85
C6TIE9	Putative uncharacterized protein (Fragment) OS	21.8	6.13
I1K5K8	Uncharacterized protein OS	35.2	5.80
I1NEN7	Uncharacterized protein OS	9.9	6.49
C6TIM4	Putative uncharacterized protein OS	22.0	6.58
I1KAY1	Uncharacterized protein OS	11.1	5.02
Q9ZS21	Lactoylglutathione lyase OS	20.9	5.90
A0A0B2PUR6	Eukaryotic translation initiation factor 3 subunit F OS	30.0	5.86
C6T2B0	Histone H2A OS	13.7	10.36
I1JQX3	Uncharacterized protein OS	58.6	6.49
I1JK20	Uncharacterized protein OS	24.6	8.88
A0A0B2SCQ8	Tubulin alpha-2 chain OS	49.6	5.10
A0A0B2RHJ1	40S ribosomal protein S24 OS	15.7	10.56
K7KXV4	Uncharacterized protein OS	7.7	11.22
A0A0B2PD59	Vacuolar-sorting receptor 1 OS	69.7	5.67
I1NEH6	Uncharacterized protein OS	36.6	7.74
A0A0B2RVZ2	Eukaryotic translation initiation factor 3 subunit F OS	31.7	5.34
A0A0R0JAG1	Catalase OS	52.8	7.03
K7KKI9	ATP synthase subunit gamma OS	30.4	8.70
A0A0R0JEI9	Uncharacterized protein OS	28.3	4.89
I1MCN3	Uncharacterized protein OS	95.7	5.22
A0A0B2NY39	S-formylglutathione hydrolase OS	31.9	7.08
I1K4U3	Uncharacterized protein OS	50.3	7.34
C6SX10	Putative uncharacterized protein OS	18.7	7.42
K7MVK2	Uncharacterized protein OS	43.7	8.25
C6T5Y4	Putative uncharacterized protein OS	22.6	5.91
I1JFN4	Uncharacterized protein OS	11.5	4.59
A0A0B2QXM2	Uncharacterized protein OS	20.9	4.88
C6TM10	Putative uncharacterized protein (Fragment) OS	11.6	10.71
I1M0X9	Uncharacterized protein OS	15.3	8.88
I1N627	Uncharacterized protein OS	31.5	5.24
K7LBB0	Uncharacterized protein OS	21.7	7.42
P24337	Hydrophobic seed protein OS	8.4	6.28
A0A0B2Q9M0	60S ribosomal protein L31 OS	13.9	9.82
A0A0B2P7Y6	Heat shock factor-binding protein 1 OS	9.7	4.41
A0A0B2PYN3	Uncharacterized protein OS	25.0	7.42
I1K089	Malate synthase OS	62.7	6.29
C6TNR9	Uncharacterized protein OS	32.2	8.32
I1M1J1	Uncharacterized protein OS	8.5	8.12

A0A0R0ERR0	Uncharacterized protein OS	47.4	5.11
A0A0B2P3S3	50S ribosomal protein L4, chloroplastic OS	12.1	4.23
C6THR4	Putative uncharacterized protein OS	10.2	8.76
K7N3H7	Uncharacterized protein OS	44.0	8.91
A0A0B2Q9R5	Histidine--tRNA ligase OS	94.9	6.64
Q944T1	Inosine-5'-monophosphate dehydrogenase-like protein (Fragment) OS	41.8	7.12
I1NFW0	Uncharacterized protein OS	30.6	5.77
A0A0B2S296	Protein disulfide isomerase-like 1-4 OS	44.7	5.41
A0A0R4J375	Uncharacterized protein OS	21.1	4.92
I1MQS8	Uncharacterized protein OS	43.8	6.32
C6SZI8	Uncharacterized protein OS	22.1	7.05
I1L6V1	Uncharacterized protein OS	47.1	8.38
A0A0R0I1A5	Uncharacterized protein OS	17.6	7.42
A0A0B2Q322	Reticulon-like protein (Fragment) OS	27.1	8.70
A0A0B2SIK3	Disease resistance response protein 206 OS	12.0	7.43
A0A0R4J3I5	Uncharacterized protein OS	39.9	5.91
A0A0B2SSS3	50S ribosomal protein L11, chloroplastic OS	13.3	10.01
K7KWA5	Uncharacterized protein OS	12.4	8.92
I1LIA0	Uncharacterized protein OS	52.0	4.92
C6TMU4	Putative uncharacterized protein OS	10.0	6.51
I1LDC2	Uncharacterized protein OS	23.2	5.74
A0A0B2Q6M1	NADH dehydrogenase [ubiquinone] iron-sulfur protein 7, mitochondrial OS	15.0	8.88
A0A0B2S6V4	Hydroxyacylglutathione hydrolase cytoplasmic OS	28.4	6.18
K7KRK6	Uncharacterized protein OS	95.1	7.24
A0A0B2RKA3	Glutamate-1-semialdehyde 2,1-aminomutase, chloroplastic OS	46.9	5.92
A0A0R0ICD7	Eukaryotic translation initiation factor 3 subunit I OS	32.4	6.70
I1MGI7	Importin subunit alpha OS	58.6	5.26
I1M7S4	Clathrin heavy chain OS	179.7	5.53
A0A0B2NWA1	Protein SEC13 like OS	32.6	6.01
K7KD43	Uncharacterized protein OS	9.1	4.78
I1LD66	Uncharacterized protein OS	44.2	9.29
A0A0R0L8P5	Proteasome subunit beta type OS	20.1	8.18
C6SZ75	Putative uncharacterized protein OS	14.5	6.51
C0J3I3	ATP synthase subunit beta (Fragment) OS	50.6	5.36
A0A0B2S959	UBA and UBX domain-containing protein OS	32.8	5.72
A0A0B2S0A3	Basic 7S globulin OS	51.2	8.00
A0A0B2RFG2	Uncharacterized protein OS	38.6	8.92
B2CM88	Chalcone-flavonone isomerase family protein (Fragment) OS	23.5	5.49
A0A0B2Q4B0	Serine--tRNA ligase OS	51.4	6.43
Q5NUF3	2-hydroxyisoflavanone dehydratase OS	35.1	6.18
A0A0B2SE95	Glycine cleavage system H protein, mitochondrial OS	17.9	5.20

K7MQT5	Uncharacterized protein OS	63.0	6.25
I1KUN7	Uncharacterized protein OS	40.2	6.54
A0A0R0EXW6	Uncharacterized protein OS	49.5	6.21
A0A0B2SBU4	Malate dehydrogenase, glyoxysomal (Fragment) OS	28.6	6.58
A0A0B2QF63	Acyl carrier protein OS	11.1	4.32
A0A0B2R6I0	NADH-cytochrome b5 reductase OS	35.6	9.03
C6SZ24	Pathogenesis-related protein OS	17.1	5.07
A0A0B2SPL4	Alpha-mannosidase OS	114.4	5.87
A0A0R0FIB8	Uncharacterized protein (Fragment) OS	16.8	4.94
I1KTW3	Uncharacterized protein OS	57.6	7.59
I1MJD3	Cysteine synthase OS	33.4	5.58
K7LDH0	Uncharacterized protein OS	17.8	9.16
C6SX20	Putative uncharacterized protein (Fragment) OS	18.8	7.39
C6T9F8	Putative uncharacterized protein OS	30.5	5.25
A0A0B2PQ07	Hsc70-interacting protein OS	42.0	4.98
A0A0R0KP00	Uncharacterized protein OS	59.3	6.62
A0A0B2R0S5	Exocyst complex component 5 OS	19.7	5.25
A0A0B2R6F6	Nuclear polyadenylated RNA-binding protein 4 OS	35.5	8.53
A0A0B2Q308	Dihydrodipicolinate reductase 2, chloroplastic OS	37.4	6.90
A0A0B2SQ91	L-ascorbate peroxidase T, chloroplastic OS	20.4	5.22
A0A0B2S5X0	Dihydroflavonol-4-reductase OS	14.6	7.44
A0A0R0F3J7	Uncharacterized protein OS	10.0	4.74
A0A0R0E5J0	Uncharacterized protein OS	17.0	4.87
A0A0B2PSC9	4-hydroxyphenylpyruvate dioxygenase OS	13.8	5.31
I1MEW6	Uncharacterized protein OS	33.2	6.11
A0A0B2R014	2-isopropylmalate synthase 2, chloroplastic OS	64.4	6.80
C6T5I8	Uncharacterized protein OS	12.8	4.94
I1L9L9	Uncharacterized protein OS	13.3	5.40
A0A0B2PZW9	Delta-1-pyrroline-5-carboxylate synthase OS	76.1	6.76
A0A0B2R6K0	Charged multivesicular body protein 1 OS	17.1	9.39
C6SWI0	Uncharacterized protein OS	26.1	5.29
A0A0R0I1X8	Uncharacterized protein OS	24.8	4.94
A0A0B2RKE1	Uncharacterized protein (Fragment) OS	13.8	4.88
I1KRT0	Uncharacterized protein OS	60.2	8.98
I1L2M6	Uncharacterized protein OS	23.2	6.64
A0A0R0FZ92	Pyrophosphate--fructose 6-phosphate 1-phosphotransferase subunit beta OS	57.9	6.71
I1MTI7	Uncharacterized protein OS	49.7	8.13
C6T918	Uncharacterized protein OS	37.1	5.05
C6T9S8	Putative uncharacterized protein OS	39.3	7.69
I1MNX4	Uncharacterized protein OS	54.0	5.85
I1L1J6	Uncharacterized protein OS	39.4	4.41
I1JY67	Uncharacterized protein OS	50.8	7.37

C6TGY4	Putative uncharacterized protein OS	22.8	9.99
A0A0B2RG73	Thioredoxin-like protein CXXS1 OS	13.7	4.70
C6TE61	Autophagy-related protein (Fragment) OS	10.2	9.74
A0A0R0J8B8	Uncharacterized protein OS	21.1	8.78
A0A0B2PUH7	Peroxidase OS	36.2	8.68
K7K9E0	Uncharacterized protein OS	15.4	7.68
A0A0B2P8V6	Glutathione synthetase OS	54.4	5.76
C6TAT2	Tau class glutathione S-transferase OS	25.6	5.74
B0FFP7	AccD (Fragment) OS	12.5	8.69
K7KZ44	Uncharacterized protein OS	26.8	9.50
C6TCJ4	Putative uncharacterized protein OS	50.1	7.43
C6TGC7	Putative uncharacterized protein (Fragment) OS	28.6	7.33
A0A0B2Q329	Dihydroflavonol-4-reductase OS	13.1	5.80
I1L540	Phosphoribulokinase OS	45.3	6.09
C6TGV4	Aldose 1-epimerase OS	40.5	9.22
C6T2N0	Uncharacterized protein OS	20.4	9.52
K7MDN2	Uncharacterized protein OS	52.6	8.03
I1K5H2	Uncharacterized protein OS	109.4	5.29
O48560	Catalase-3 OS	56.9	7.25
A0A0R0F0P9	Uncharacterized protein OS	11.8	9.20
I1N620	Plasma membrane ATPase OS	96.9	6.67
I1K1X3	Uncharacterized protein OS	15.6	4.81
C6TIE4	Putative uncharacterized protein OS	18.4	6.90
K7LNQ6	Thioredoxin reductase OS	40.0	8.21
A0A0R4J460	Uncharacterized protein OS	46.3	9.41
C6SYA9	Putative uncharacterized protein OS	14.7	5.33
A0A0B2RZZ6	Polyadenylate-binding protein-interacting protein 2 OS	14.7	5.05
A0A0R0J2M7	Uncharacterized protein OS	20.7	8.07
I1LT59	Uncharacterized protein OS	16.9	7.75
A0A0B2Q0U3	Uncharacterized protein OS	15.2	4.64
A0A0B2Q2D0	Eukaryotic initiation factor iso-4F subunit p82-34 OS	86.2	8.15
C6SZX0	Uncharacterized protein OS	17.2	7.81
A0A0B2RF58	DEAD-box ATP-dependent RNA helicase 37 OS	45.5	8.70
C6SX81	Uncharacterized protein OS	15.5	4.77
I1JZT5	Plasma membrane ATPase OS	105.0	6.73
A0A0B2NW37	Cytochrome c oxidase subunit 5b-2, mitochondrial OS	11.3	4.67
I1KHR5	Uncharacterized protein OS	20.0	5.91
K7MQS5	Uncharacterized protein OS	32.6	8.79
I1KD00	Uncharacterized protein OS	83.3	6.61
A0A0B2Q963	26S proteasome non-ATPase regulatory subunit 1 OS	109.5	5.36
A0A0B2Q6R1	Endoplasmic reticulum vesicle protein 25 OS	18.7	7.11
I1JMB4	Glyceraldehyde-3-phosphate dehydrogenase OS	44.6	8.50
A0A0R0KKJ8	Uncharacterized protein OS	20.4	9.11

A0A0B2R243	DEAD-box ATP-dependent RNA helicase 8 OS	37.1	7.81
I1MSU2	Uncharacterized protein OS	18.5	5.57
I1MMY1	Uncharacterized protein OS	41.6	5.59
C6TNT7	Putative uncharacterized protein (Fragment) OS	23.3	9.10
C6SVM8	Uncharacterized protein OS	15.1	7.31
A0A0R0H092	Uncharacterized protein OS	90.3	6.76
A0A0B2QQE7	Stellacyanin OS	34.0	9.03
K7LBP6	Uncharacterized protein OS	30.2	6.00
A0A0B2SVQ0	Succinyl-CoA ligase subunit beta OS	45.2	5.99
A4ZFE0	CETS1 OS	19.0	9.04
C6SVI7	Uncharacterized protein OS	21.0	6.02
A0A0B2QX18	Glucose-6-phosphate isomerase (Fragment) OS	47.9	5.19
I1JZH5	Uncharacterized protein OS	14.4	9.38
K7M828	Uncharacterized protein OS	120.9	5.43
A0A0R0HCT4	Uncharacterized protein OS	15.2	10.48
C6T2P6	Putative uncharacterized protein OS	19.0	8.32
A0A0B2RPU9	Aconitate hydratase OS	107.1	7.56
A0A0B2SL96	Hydroxyacylglutathione hydrolase 3, mitochondrial OS	28.0	6.64
C6TGU6	Uncharacterized protein OS	45.1	4.81
C6SVP2	Uncharacterized protein OS	16.2	6.23
I1LHV0	Uncharacterized protein OS	22.1	6.37
I1KG06	Methylenetetrahydrofolate reductase OS	53.4	7.34
A0A0R0KPW1	Uncharacterized protein (Fragment) OS	64.9	8.53
I1ND83	Carboxypeptidase OS	45.7	6.57
A0A0B2SLQ8	NADPH:quinone oxidoreductase OS	21.5	8.82
A0A0B2PA86	Acyl-protein thioesterase 2 OS	22.8	8.72
I1KV72	Uncharacterized protein OS	17.4	4.25
A0A0B2S1Q3	Uncharacterized protein OS	22.6	5.52
C6T624	Putative uncharacterized protein OS	24.7	10.11
A0A0B2R152	Uncharacterized protein OS	62.4	6.38
A0A0B2SFR1	Uncharacterized protein OS	18.5	5.74
I1M654	Uncharacterized protein OS	42.5	8.37
I1N697	Uncharacterized protein OS	45.4	5.40
C6SWJ2	Prefoldin subunit 3 OS	21.0	4.86
I1JCU3	Uncharacterized protein OS	19.0	7.36
A0A0B2SGE1	Uncharacterized protein OS	39.8	6.32
K7MRP1	Uncharacterized protein OS	24.9	8.40
C6T5V2	Cysteine proteinase inhibitor (Fragment) OS	13.5	9.38
C6TI49	Putative uncharacterized protein OS	22.4	6.23
A0A0B2Q052	Alpha-xylosidase OS	102.8	6.64
A0A0B2RHA2	UMP/CMP kinase OS	16.2	5.48
I1K0K8	Uncharacterized protein OS	55.2	6.43
A0A0R0G3K3	Uncharacterized protein OS	13.4	6.77

C6SX98	Uncharacterized protein OS	17.2	9.33
A0A0B2QQC5	Polyadenylate-binding protein OS	64.3	7.59
A0A0R0GYC5	Uncharacterized protein OS	16.1	6.52
A0A0B2PR99	Tocopherol cyclase, chloroplastic OS	54.9	6.21
I1JYD9	Uncharacterized protein OS	37.1	9.28
C6TFB9	Putative uncharacterized protein OS	18.2	10.56
I1MMK6	Uncharacterized protein OS	24.1	9.92
K7L5A8	Uncharacterized protein OS	47.4	7.20
I1KSA3	Uncharacterized protein OS	18.5	7.68
C6SZI9	Uncharacterized protein OS	16.8	4.77
A0A0B2QYJ6	DEAD-box ATP-dependent RNA helicase 2 OS	46.0	6.47
I1LPT9	Uncharacterized protein OS	23.1	8.37
C6TML0	Putative uncharacterized protein OS	33.1	6.65
A0A0R0K5Q3	Uncharacterized protein OS	34.1	7.84
K7MBR2	Uncharacterized protein OS	29.3	5.72
I1LJ15	Uncharacterized protein OS	46.3	5.64
A0A0B2P7H5	Uncharacterized protein OS	15.2	11.65
I1JFI6	Uncharacterized protein OS	14.3	7.88
K7MSG9	Uncharacterized protein OS	41.4	6.77
I1NGH8	Uncharacterized protein OS	27.1	5.55
C6SYK1	Putative uncharacterized protein OS	19.4	4.96
A0A0R0ILA7	Uncharacterized protein OS	18.4	6.54
A0A0B2PW43	Glycine-rich RNA-binding protein 2, mitochondrial OS	17.0	4.96
A0A0B2QFU7	L-ascorbate oxidase like OS	34.3	9.00
C6SZ51	Putative uncharacterized protein OS	25.9	5.54
A0A0B2SNT8	Heat shock 22 kDa protein, mitochondrial OS	19.9	5.40
I1LWU6	Uncharacterized protein OS	51.3	6.74
A0A0B2RSD5	Glutamate dehydrogenase OS	44.5	6.40
C6TEF1	Uncharacterized protein OS	40.8	7.36
I1JEV3	Uncharacterized protein OS	68.4	9.09
K7MQ64	Uncharacterized protein OS	15.3	6.54
C6T9I0	Putative uncharacterized protein (Fragment) OS	30.9	4.84
A0A0B2NPD9	Heme-binding-like protein, chloroplastic OS	22.3	5.57
I1KYY5	Uncharacterized protein OS	20.5	5.72
A0A0R0JFH3	Uncharacterized protein OS	20.4	5.24
A0A0B2PBZ1	D-3-phosphoglycerate dehydrogenase OS	62.6	6.42
A0A0R0HVH0	Phosphoinositide phospholipase C OS	51.0	6.48
I1NFX7	D-3-phosphoglycerate dehydrogenase OS	62.8	6.58
I1NIP9	Uncharacterized protein OS	37.2	6.10
A0A0R0KW50	Uncharacterized protein OS	25.4	5.15
I1JQU8	Uncharacterized protein OS	18.2	9.58
A0A0B2SDT6	Peptidyl-prolyl cis-trans isomerase FKBP62 OS	64.7	5.21
A0A097PNA4	AT5G11810-like protein (Fragment) OS	25.6	6.37

A0A0R0EM38	Adenylosuccinate synthetase, chloroplastic OS	50.9	9.29
A0A0B2RUF4	D-2-hydroxyglutarate dehydrogenase, mitochondrial OS	62.1	7.27
I1LJG9	Uncharacterized protein OS	83.8	5.77
I1MPF4	Glycosyltransferase OS	53.7	6.84
A0A0B2RKR0	Coatomer subunit gamma OS	98.5	5.21
A0A0B2P7C1	Cullin-associated NEDD8-dissociated protein 1 OS	131.2	5.67
A0A0R0EV02	Uncharacterized protein OS	32.7	8.69
K7MJZ7	Uncharacterized protein OS	21.4	4.42
A0A0B2RXT2	Glycine dehydrogenase [decarboxylating], mitochondrial OS	21.6	5.43
K7LEH7	Uncharacterized protein OS	26.6	6.37
A0A0B2QMQ2	Putative glucose-6-phosphate 1-epimerase OS	31.6	8.46
I1JSC6	Uncharacterized protein OS	28.8	10.45
A0A0B2RKR9	Patellin-3 OS	49.0	5.44
K7MRB1	Uncharacterized protein OS	21.0	6.70
A0A0R0FUR5	Uncharacterized protein OS	29.6	5.62
A0A0B2R5T6	Malic enzyme (Fragment) OS	64.8	6.32
C6THQ1	Uncharacterized protein OS	25.3	9.51
A0A0B2NV17	Uncharacterized protein OS	19.4	9.92
A0A0B2PXW0	Pumilio like 5 (Fragment) OS	20.9	6.67
I1M903	Uncharacterized protein OS	69.1	5.86
I1N790	Uncharacterized protein OS	30.9	5.92
C6T7H6	Putative uncharacterized protein (Fragment) OS	23.4	6.80
A0A0R0H152	Uncharacterized protein OS	37.1	6.19
C6TDY6	Putative uncharacterized protein OS	26.2	6.39
I1L8F0	Uncharacterized protein OS	56.6	6.64
A0A0B2SQU7	Acetyl-CoA acetyltransferase, cytosolic 1 OS	41.5	6.57
I1LN41	Uncharacterized protein OS	84.5	6.30
A0A0B2SIS3	Peroxidase OS	34.0	7.61
A0A0R0ESU5	Uncharacterized protein OS	48.9	5.29
I1NFK8	Uncharacterized protein OS	84.8	6.52
A0A0B2QYH4	MOSC domain-containing protein 1, mitochondrial OS	35.4	5.59
A0A0R0KFI8	Uncharacterized protein OS	38.4	6.20
A0A0B2S5A6	Isocitrate dehydrogenase [NADP] OS	35.4	6.73
A0A0R0JR59	Uncharacterized protein OS	31.5	6.24
A0A0B2PBU3	Putative glutathione S-transferase OS	26.0	5.53
I1L2V4	Uncharacterized protein OS	37.5	5.43
C6T891	Uncharacterized protein OS	32.7	9.41
A0A0B2PR31	ATP-dependent Clp protease proteolytic subunit OS	33.3	6.57
A0A0B2NXF9	66 kDa stress protein OS	61.9	7.15
K7KUJ2	Uncharacterized protein OS	34.2	7.12
C6TDX0	ATP-dependent Clp protease proteolytic subunit (Fragment) OS	40.4	8.57
A0A0B2SFS3	Peroxygenase 2 OS	35.9	8.68
A0A0B2R667	Heme-binding protein 2 OS	26.7	8.56

B1ACD3	Putative Kunitz trypsin protease inhibitor OS	23.6	5.38
A0A0R0EM30	Uncharacterized protein (Fragment) OS	27.0	8.15
A0A0B2Q542	Mitochondrial-processing peptidase subunit alpha OS	54.7	5.77
O49014	Beta-1,3-glucanase 7 (Fragment) OS	27.1	7.53
A0A0B2SSD9	Coatomer subunit beta'-2 OS	102.1	5.11
A0A0R0L7G1	Uncharacterized protein OS	26.1	8.09
M1FP70	ATPase subunit 4 OS	21.8	10.17
C4T848	Protein disulfide isomerase like protein OS	58.8	4.98
I1J702	Uncharacterized protein OS	42.8	5.17
A0A0B2PGV9	Serine/threonine-protein kinase-like protein ACR4 OS	21.7	6.89
I1KGT4	Uncharacterized protein OS	28.9	9.61
A0A0R0HHC1	Uncharacterized protein (Fragment) OS	68.3	9.23
C6TCA2	Putative uncharacterized protein OS	25.7	9.83
I1NAK9	Uncharacterized protein OS	98.8	5.64
C6SY32	Putative uncharacterized protein OS	26.1	8.92
C6T692	Putative uncharacterized protein (Fragment) OS	26.3	6.30
A0A0B2RY30	Coatomer subunit beta OS	105.9	5.81
I1KGF2	Uncharacterized protein OS	54.2	6.14
C6SW58	Putative uncharacterized protein (Fragment) OS	30.1	8.72
C6T4T2	Putative uncharacterized protein OS	25.5	8.69
A0A0R0EK54	Uncharacterized protein OS	56.5	6.57
K7MHB8	Uncharacterized protein OS	38.6	8.05
C6TGW5	Peroxidase (Fragment) OS	30.4	5.00
C6TGF5	Uncharacterized protein OS	31.8	7.69
A0A0B2QEC0	Alpha-glucan water dikinase, chloroplastic OS	24.6	5.11
A0A0B2SBD7	Uncharacterized protein OS	41.8	6.79
A0A0R0LK68	Uncharacterized protein OS	35.8	8.63
I1MQQ0	Uncharacterized protein OS	34.0	9.77
I1LEY1	Uncharacterized protein OS	65.4	5.03
A0A0R0KGJ7	Uncharacterized protein OS	48.1	6.96
A0A0B2R4V3	Uncharacterized protein OS	26.5	9.45
A0A0R4J2Q1	Uncharacterized protein OS	46.4	8.34
I1M370	Uncharacterized protein OS	48.3	7.05
K7LCD1	Uncharacterized protein OS	56.8	5.01
A0A0R0HW38	Uncharacterized protein OS	61.4	5.53
A0A0B2SPS6	Heat shock protein 90 OS	89.9	5.20
A0A0B2RXE8	Epidermis-specific secreted glycoprotein EP1 OS	38.9	8.70
A0A0B2PWJ5	Xanthoxin dehydrogenase OS	29.3	6.79
A0A0B2SGA1	Protein transport protein Sec24-like OS	108.6	8.12
I1N6K4	Uncharacterized protein OS	32.0	9.26
A0A0B2Q4V1	Putative sucrose-phosphate synthase OS	118.1	6.52
I1MNH0	Uncharacterized protein OS	51.6	3.97
I1LG42	Protein phosphatase methylesterase 1 OS	35.4	6.52

A0A0B2PMS7	Aspartic proteinase nepenthesin-1 OS	31.7	9.31
C6T7Q4	Putative uncharacterized protein OS	43.9	9.13
C6TI56	Putative uncharacterized protein OS	37.4	5.44
I1MBF5	Uncharacterized protein OS	51.2	6.14
A0A0R4J5U6	Uncharacterized protein OS	38.6	6.70
I1LHR8	Succinate dehydrogenase [ubiquinone] flavoprotein subunit, mitochondrial OS	60.1	6.14
A0A0B2SMH5	RuvB-like helicase OS	47.1	5.41
I1KAW8	Uncharacterized protein OS	47.4	7.23
A0A0B2R1G9	Phosphotransferase OS	46.8	5.86
K7L456	Uncharacterized protein OS	43.2	9.20
I1KYS8	Uncharacterized protein OS	41.1	7.85
I1JAQ7	Uncharacterized protein OS	45.2	6.84
I1J418	Serine/threonine-protein phosphatase OS	32.7	5.10
A0A0B2PLE2	ABC transporter F family member 3 OS	68.8	6.90
A0A0B2RKV6	L-ascorbate oxidase like OS	60.0	9.44
I1KNN0	ATP-dependent 6-phosphofructokinase OS	51.1	6.71
A0A0B2PMB9	Uncharacterized protein OS	41.6	7.87
K7K578	Uncharacterized protein OS	57.4	8.25
A0A0B2NYA8	Carboxypeptidase OS	53.1	6.04
A0A0R0FDK6	Uncharacterized protein OS	48.9	6.11
I1NEX1	Uncharacterized protein OS	60.7	5.67
K7MKQ6	Uncharacterized protein OS	52.5	5.48
A0A0B2REM4	Nudix hydrolase 3 OS	48.9	5.14
I1LIG6	Uncharacterized protein OS	64.8	7.55
G7YZH4	Purple acid phosphatase OS	55.4	8.16
K7KMA4	Coatomer subunit alpha OS	135.2	7.18
A0A0B2SJ49	L-ascorbate oxidase like OS	54.5	7.61
K7MTE5	Uncharacterized protein OS	50.5	6.10
A0A0R0J6H9	Mannose-6-phosphate isomerase OS	35.4	6.30
A0A0B2Q1T9	Tryptophan synthase beta chain 2 OS	54.8	7.49
I1J8M6	Uncharacterized protein OS	41.0	7.01
A0A0R0K2N5	Uncharacterized protein OS	49.3	7.71
A0A0B2SHB0	Sorting nexin 2B OS	46.5	4.91
I1M0Z8	Uncharacterized protein OS	129.9	5.48
A0A0B2R5N5	Protein TIC 55, chloroplastic OS	60.0	8.76
A0A0R0HFS7	Adenylyl cyclase-associated protein OS	43.0	8.78
I1NJC3	Uncharacterized protein OS	50.3	6.21
K7LPJ2	Uncharacterized protein OS	45.6	6.29
I1K2B5	Uncharacterized protein OS	79.6	6.60
A0A0B2SPQ5	Fimbrin-like protein 2 OS	74.4	5.55
I1LBC0	Lipoxygenase OS	69.5	6.06
A0A0B4PJM3	Beta-glucosidase OS	68.7	8.95

A0A0R0GGZ9	Uncharacterized protein OS	65.3	5.27
A0A0B2QUM6	Dipeptidyl peptidase 8 OS	86.8	6.83
K7MV60	Uncharacterized protein OS	53.0	6.40
A0A0B2PJF5	Putative endo-1,3(4)-beta-glucanase 2 OS	76.9	6.10
A0A0B2QUV3	V-type proton ATPase subunit a OS	91.7	5.96
A0A0R0HE22	Uncharacterized protein OS	87.5	6.86
A0A0B2RJB4	Importin-5 OS	93.3	4.79
A0A0R0KIR5	Uncharacterized protein (Fragment) OS	166.5	6.25
I1MU68	Uncharacterized protein OS	108.3	7.59
I1MG11	Uncharacterized protein OS	76.7	6.52
A0A0B2PR14	Mechanosensitive ion channel protein 8 OS	80.3	8.85
I1LIM0	Uncharacterized protein OS	92.0	5.15
I1J495	Uncharacterized protein OS	87.5	6.90
A0A0B2P1M7	U-box domain-containing protein 35 (Fragment) OS	79.2	7.90
I1LTQ5	Uncharacterized protein OS	113.7	6.25
A0A0B2SBV9	AP-4 complex subunit epsilon OS	79.3	5.50
A0A0B2R2W9	Multiple C2 and transmembrane domain-containing protein 2 OS	84.9	9.14
A0A0R0EW54	Uncharacterized protein OS	61.6	5.29
A0A0B2RZM2	Splicing factor 3B subunit 3 OS	134.8	5.55
A0A0B2SSS8	Beta-galactosidase OS	102.4	6.57
I1JQT6	Uncharacterized protein OS	111.0	5.40
K7KNC8	Uncharacterized protein OS	82.0	8.46
I1L922	Uncharacterized protein OS	92.8	6.48
I1MUR0	Uncharacterized protein OS	65.5	4.73
I1JAD5	Uncharacterized protein OS	70.7	6.55
I1M0N4	Uncharacterized protein OS	124.1	6.00
A0A0B2SQP4	Clustered mitochondria protein homolog OS	154.1	6.33
I1MWC4	Uncharacterized protein OS	110.8	6.05
A0A0B2PR75	Acylamino-acid-releasing enzyme OS	89.6	7.06
A0A0R4J2L6	Uncharacterized protein OS	121.3	6.13
I1JSY1	Uncharacterized protein OS	82.0	5.62
A0A0B2S0J5	Beta-galactosidase OS	126.5	6.18
K7MAY3	Uncharacterized protein OS	140.6	6.04
A0A0B2RLA1	Translational activator GCN1 OS	143.6	7.15
A0A0B2SIT5	Glutamate synthase [NADH], amyloplastic (Fragment) OS	225.9	6.47
A0A0B2QJF4	Zinc finger CCCH domain-containing protein 44 OS	155.0	5.21
Q39850	Acetyl-CoA carboxylase (Fragment) OS	148.9	6.68
I1M5U8	Uncharacterized protein OS	275.7	8.70
C6T1E4	Uncharacterized protein OS	11.4	4.37
C6SW79	40S ribosomal protein S12 OS	14.8	5.49
Q39762	Histone H3 (Fragment) OS	6.5	11.33
A0A0B2RYF1	UTP--glucose-1-phosphate uridylyltransferase OS	56.0	6.25

I1L8V8	Uncharacterized protein OS	17.7	8.70
I1L571	60S ribosomal protein L13 OS	23.8	11.02
C6T4Z6	Uncharacterized protein OS	15.9	10.18
I1JQN0	Uncharacterized protein OS	19.4	11.43
I1JAE3	Uncharacterized protein OS	42.8	7.50
C6T073	Uncharacterized protein OS	21.2	8.63
I1JXA0	Uncharacterized protein OS	90.8	5.24
C6T1S1	Putative uncharacterized protein OS	19.9	9.67
I1KVT5	Uncharacterized protein OS	15.5	6.01
C6SY65	Ribosomal protein L19 OS	24.8	11.55
C6SWV2	Uncharacterized protein OS	12.2	7.42
C6TCM8	Putative uncharacterized protein OS	49.4	5.10
C6TNU2	Putative uncharacterized protein OS	47.5	6.02
I1K0S0	Ribosomal protein L15 OS	24.2	11.59
A0A0B2PLW6	60S ribosomal protein L26-1 OS	16.7	10.92
I1N898	Uncharacterized protein OS	49.9	5.10
A0A0R0FE22	Uncharacterized protein OS	14.9	10.90
I1LWV7	Uncharacterized protein OS	40.5	5.77
A0A0B2QL11	C-1-tetrahydrofolate synthase, cytoplasmic OS	26.0	7.49
A0A0R0K0R3	Uncharacterized protein OS	9.6	10.81
C6T9I2	Putative uncharacterized protein OS	16.4	8.85
I1N1C8	Uncharacterized protein OS	9.3	9.36
A0A0R0EV63	Uncharacterized protein (Fragment) OS	16.9	9.23
Q96447	Actin (Fragment) OS	37.0	5.69
I1JR56	Uncharacterized protein OS	19.5	6.55
A0A0R0IQ92	Phospholipase D OS	89.2	5.80
C6T8K3	Putative uncharacterized protein OS	27.4	5.30
C6TN64	Proteasome subunit alpha type OS	25.0	4.82
C6TBW7	Uncharacterized protein OS	23.9	7.83
A0A0R0GKV9	Uncharacterized protein OS	9.6	9.01
I1LDR2	Uncharacterized protein OS	50.4	4.83
C6T9H8	Putative uncharacterized protein OS	5.2	9.99
K7MT91	Uncharacterized protein OS	32.3	6.73
A0A0B2RSC0	Formate--tetrahydrofolate ligase OS	67.6	7.50
A0A0B2RQ63	Adenylate kinase B OS	26.6	8.12
A0A0B2QG54	14-3-3-like protein GF14 iota OS	28.4	4.88
A0A0R0FLS7	Uncharacterized protein OS	16.7	5.88
A0A0B2REI8	6-phosphogluconate dehydrogenase, decarboxylating OS	54.0	6.95
C6TJP4	Putative uncharacterized protein OS	42.9	6.70
C6T1U5	Putative uncharacterized protein OS	12.0	8.94
A0A0B2QZB3	Sulfurtransferase OS	39.1	5.69
A0A0B2REL4	UBA and UBX domain-containing protein OS	27.3	6.57
A0A0B2SU49	Eukaryotic translation initiation factor 4E-2 OS	9.6	5.45

D2DKE6	Ketol-acid reductoisomerase OS	63.5	7.08
I1K6B9	6-phosphogluconate dehydrogenase, decarboxylating OS	53.8	6.70
C6SWC7	Putative uncharacterized protein OS	25.8	10.13
I1LMP5	Eukaryotic translation initiation factor 3 subunit F OS	30.7	5.33
I1L314	Heat shock protein 90-1 OS	80.3	5.06
A0A0B2NRZ7	Uncharacterized protein OS	14.0	9.06
I1KWI0	Uncharacterized protein OS	40.1	5.92
K7MWG5	Uncharacterized protein OS	10.5	6.14
A0A0B2RCZ5	Enoyl-[acyl-carrier-protein] reductase [NADH], chloroplastic OS	39.3	8.56
A0A0B2PU85	Outer envelope pore protein 24, chloroplastic (Fragment) OS	6.5	9.60
I1LLS2	Uncharacterized protein OS	28.2	5.78
C6TAE6	Putative uncharacterized protein OS	22.2	4.68
A0A0B2R027	Dihydrodipicolinate reductase 2, chloroplastic OS	37.4	6.84
A0A0B2SN49	NADP-dependent glyceraldehyde-3-phosphate dehydrogenase OS	53.1	7.61
A0A0B2P437	26S protease regulatory subunit 4 like OS	37.0	5.29
C6SY14	Uncharacterized protein OS	11.0	9.35
C6T9M0	Putative uncharacterized protein OS	52.3	7.01
C6SW35	Uncharacterized protein OS	13.8	9.86
I1N383	Uncharacterized protein OS	68.6	5.80
C6SWG5	Uncharacterized protein OS	11.2	4.88
A0A0R0GMQ7	Proteasome subunit beta type OS	21.0	5.24
A0A0B2PSK8	Desiccation-related protein PCC13-62 OS	15.5	6.80
A0A0B2RGS6	Putative quinone-oxidoreductase like, chloroplastic OS	34.6	8.19
A0A0B2P1V9	GTP-binding protein SAR1A OS	22.1	6.35
K7LZ61	Uncharacterized protein OS	8.7	8.06
A0A0B2NW31	Uncharacterized protein OS	14.7	5.35
I1KQH2	Uncharacterized protein OS	53.1	5.20
C6SZB9	Putative uncharacterized protein OS	22.6	9.67
C6T491	Uncharacterized protein OS	11.1	5.54
A0A0R0G1M8	Sucrose synthase OS	89.1	6.11
A0A0B2PKM5	Glucose-6-phosphate isomerase OS	67.3	5.94
A0A0R0JQZ9	Uncharacterized protein OS	44.5	5.66
I1N675	Uncharacterized protein OS	40.5	4.74
K7K8K7	Uncharacterized protein OS	10.7	8.66
A0A0B2S8L6	Elongation factor P OS	25.5	8.51
C6SWY4	Non-specific lipid-transfer protein OS	12.5	8.41
A0A0B2Q9M6	Glutathione peroxidase OS	18.8	5.72
A0A0B2Q0Q7	LYR motif-containing protein 4 OS	10.8	10.10
A0A0B2Q943	40S ribosomal protein S15 OS	13.5	10.86
A0A0R0KB25	Uncharacterized protein OS	40.5	8.37
K7MV98	Eukaryotic translation initiation factor 3 subunit H OS	29.2	4.60

C6T1W3	Uncharacterized protein OS	12.6	6.87
A0A0B2S8S5	Epoxide hydrolase 2 OS	35.3	5.52
I1KPV6	Derlin OS	27.9	9.88
I1JAG0	Uncharacterized protein OS	21.9	4.96
A0A0B2P8I5	Sulfite reductase [ferredoxin] OS	77.2	9.16
I1JAZ4	Uncharacterized protein OS	15.1	5.17
K7MYQ2	Uncharacterized protein OS	74.7	5.92
I1M8M2	Uncharacterized protein OS	10.1	8.63
I1LF88	Uncharacterized protein OS	16.5	8.13
I1JS44	Uncharacterized protein OS	21.5	9.82
I1K4T5	Uncharacterized protein OS	13.4	8.88
I1JT75	Uncharacterized protein OS	13.1	5.33
K7LUP3	Uncharacterized protein OS	14.9	9.38
A0A0B2RDT8	Putative fructokinase-5 OS	33.9	6.43
I1LG43	Protein phosphatase methylesterase 1 OS	38.6	6.60
I1LJW6	Uncharacterized protein OS	16.4	5.24
A0A0B2SVB7	Inosine-5'-monophosphate dehydrogenase OS	33.3	7.43
Q42804	Phosphoribosylamine--glycine ligase (Fragment) OS	42.2	6.54
A0A0B2PQQ2	Ribosomal protein OS	14.0	9.00
I1JFA3	Uncharacterized protein OS	27.5	9.63
A0A0B2Q2N3	Nucleoside diphosphate kinase OS	22.9	9.42
A0A0R0IT34	Uncharacterized protein OS	23.6	6.09
A0A0B2PM87	26S proteasome non-ATPase regulatory subunit 2 1A OS	97.2	5.30
A0A0B2SXN3	30S ribosomal protein 2, chloroplastic OS	26.4	9.38
C6T9L0	Putative uncharacterized protein OS	24.5	6.96
C6T2F1	Uncharacterized protein OS	16.5	6.92
C6T9V7	Putative uncharacterized protein OS	21.2	8.07
K7MNE0	Uncharacterized protein OS	15.3	5.39
I1NGR9	Uncharacterized protein OS	21.4	6.06
A0A0R0EZE7	Uncharacterized protein OS	15.3	6.55
A0A0B2SBZ3	50S ribosomal protein L19, chloroplastic OS	12.9	10.81
A0A0B2RF77	Dolichol-phosphate mannosyltransferase OS	17.2	9.38
K7N2X5	Uncharacterized protein OS	42.3	7.01
A0A0R0KNL8	Histone H2A OS	13.3	10.36
C6TG38	Putative uncharacterized protein OS	19.3	8.43
K7LSI9	Uncharacterized protein OS	65.1	7.18
K7L7D2	Uncharacterized protein OS	35.4	5.72
C6T085	Putative uncharacterized protein OS	19.2	8.15
K7MKL7	Uncharacterized protein OS	15.1	8.97
C6TKZ2	Uncharacterized protein OS	38.5	7.17
I1MH05	Uncharacterized protein OS	103.4	5.97
A0A0B2QYH8	Uncharacterized protein OS	16.9	8.76
K7MD76	Uncharacterized protein OS	29.1	9.19

A0A0B2RCR5	Eukaryotic translation initiation factor 3 subunit B OS	32.8	7.52
A0A0B2QAU8	60S ribosomal export protein NMD3 OS	27.4	5.94
A0A0B2SH89	Aldose 1-epimerase OS	33.2	6.71
A0A0R0GGB5	Uncharacterized protein OS	36.2	8.75
A0A0B2QLW2	Serine/threonine-protein phosphatase 2A 65 kDa regulatory subunit A beta isoform OS	57.8	5.24
I1KNG1	Uncharacterized protein OS	55.4	6.90
A0A0B2NYH8	Quinone oxidoreductase-like protein 2 like OS	12.5	5.45
C6TB96	Putative uncharacterized protein OS	35.2	5.49
K7MH03	Uncharacterized protein OS	24.7	7.49
A0A0B2PGL6	Stress-related protein OS	22.3	8.70
A0A0B2QJV1	Protein MOTHER of FT and TF 1 OS	22.1	9.29
I1JIJ5	Vacuolar protein sorting-associated protein 29 OS	18.7	6.25
I1KN77	Uncharacterized protein OS	25.5	5.60
A0A0B2NXZ0	Cytochrome c1-1, heme protein, mitochondrial OS	15.7	8.85
K7K0U1	Fe-S cluster assembly protein DRE2 OS	22.4	5.71
A0A0B2S8R7	Putative GDP-L-fucose synthase 2 OS	35.9	6.80
C6TA66	Putative uncharacterized protein OS	19.5	9.48
C6SXY5	Ubiquitin-fold modifier-conjugating enzyme 1 OS	19.7	7.78
I1LA26	Uncharacterized protein OS	25.4	6.54
C6TN31	Putative uncharacterized protein OS	35.6	8.72
C6T4S0	Putative uncharacterized protein OS	17.5	6.35
C6SYV3	Putative uncharacterized protein OS	17.8	9.61
I1JYS5	Uncharacterized protein OS	24.4	6.40
A0A0R0GPF6	Uncharacterized protein (Fragment) OS	21.5	8.12
A0A0B2SUY4	Chaperone protein ClpB1 OS	101.3	6.28
A0A0B2RR79	3-phosphoshikimate 1-carboxyvinyltransferase OS	55.8	7.11
A0A0R0HZX1	Uncharacterized protein OS	19.4	5.11
C6SZI5	Uncharacterized protein OS	19.0	8.62
A0A0B2RYL4	Phosphoglucan, water dikinase, chloroplastic OS	130.3	5.88
I1M9U5	Uncharacterized protein OS	19.8	11.08
I1MUD7	Uncharacterized protein OS	23.6	6.34
A0A0R4J5A2	Peroxidase OS	38.5	7.09
A0A0B2Q5U8	Putative oxidoreductase ykwC OS	14.5	8.00
I1J575	Uncharacterized protein OS	28.8	8.51
I1L3K2	Uncharacterized protein OS	19.7	8.78
A0A0B2P363	Ubiquitin-activating enzyme E1 1 OS	110.6	5.38
A0A0R0KJ23	Uncharacterized protein OS	32.2	6.80
I1K843	Uncharacterized protein OS	43.6	5.08
A0A0B2RK57	Far upstream element-binding protein 1 OS	54.6	5.73
A0A0B2SWD3	Anthocyanin 5-aromatic acyltransferase OS	26.2	6.27
A0A0R0K7P8	Uncharacterized protein OS	110.4	5.76
A0A0B2Q296	Glucan endo-1,3-beta-glucosidase OS	36.2	9.04

C6TL46	Putative uncharacterized protein OS	29.6	6.23
A0A0R0H948	Uncharacterized protein OS	94.4	5.25
I1KNL7	Uncharacterized protein OS	42.3	7.93
A0A0B2RGL2	Polyadenylate-binding protein RBP47 OS	28.5	7.50
A0A0B2NUA2	Putative oxidoreductase OS	25.0	5.81
A0A0B2P161	Pyrophosphate-energized vacuolar membrane proton pump OS	69.7	6.34
I1LI17	Uncharacterized protein OS	38.3	4.58
A0A0R0G0K3	Uncharacterized protein OS	51.0	6.93
K7LJP7	Uncharacterized protein OS	28.6	7.55
A0A0B2SB49	Putative glucose-6-phosphate 1-epimerase OS	24.6	5.58
I1JBE3	Uncharacterized protein OS	75.0	7.21
C6T4W9	Putative uncharacterized protein OS	26.1	4.44
A0A0B2RK17	Leucine--tRNA ligase, cytoplasmic OS	127.2	6.79
A0A0B2P6J5	Galactokinase OS	54.1	6.38
I1KBE4	Uncharacterized protein OS	26.8	10.20
A0A0B2QS12	Anthocyanidin 3-O-glucosyltransferase OS	24.3	8.97
C6SYD9	Putative uncharacterized protein OS	24.7	5.94
K7KB83	Uncharacterized protein OS	23.7	6.79
I1MTU9	Uncharacterized protein OS	26.3	7.88
C6T5J7	Putative uncharacterized protein (Fragment) OS	27.2	6.79
I1KFF0	Uncharacterized protein OS	49.4	6.99
A0A0B2SGJ8	Heat shock protein 90 OS	89.3	5.36
A0A0B2PF75	Uncharacterized protein OS	39.2	5.74
K7KQ65	Uncharacterized protein OS	31.8	9.28
A0A0R4J5C5	Uncharacterized protein OS	27.6	7.30
C6TD54	Putative uncharacterized protein OS	47.0	7.53
A0A0B2PEA5	Sedoheptulose-1,7-bisphosphatase, chloroplastic OS	41.7	6.43
C6THU7	Putative uncharacterized protein OS	31.5	7.27
A0A0B2S2A3	Beta-galactosidase OS	91.2	6.92
I1N3E1	Uncharacterized protein OS	46.9	8.16
I1KYR5	Uncharacterized protein OS	146.1	5.91
A0A0R0KPC8	Uncharacterized protein OS	29.8	5.73
A0A0B2Q2U4	Uncharacterized protein OS	27.8	8.75
C6TL93	Putative uncharacterized protein OS	41.9	6.04
K7KTU9	Uncharacterized protein OS	36.0	5.33
A0A0B2PG31	Acetyl-coenzyme A carboxylase carboxyl transferase subunit alpha, chloroplastic OS	76.0	8.81
C6T041	Putative uncharacterized protein (Fragment) OS	24.6	5.55
K7LA98	Uncharacterized protein OS	71.5	6.89
K7MY54	Uncharacterized protein OS	119.4	6.90
K7LKA7	Uncharacterized protein OS	47.5	7.55
A0A0B2PIK7	NAD(P)H-hydrate epimerase OS	37.9	4.87
I1M6U9	Small nuclear ribonucleoprotein-associated protein OS	29.4	11.28

C6T7S9	Putative uncharacterized protein OS	31.0	6.07
A0A0B2QYN4	2-oxoglutarate/malate translocator, chloroplastic OS	38.2	7.36
A0A0B2RE17	Trans-cinnamate 4-monooxygenase OS	50.0	8.21
I1KNX5	Aldehyde dehydrogenase OS	59.7	7.34
I1MHY5	Uncharacterized protein OS	58.4	7.74
A0A0R0FYU5	Uncharacterized protein OS	42.4	5.87
A0A0B2QNT4	Dihydroorotate dehydrogenase (quinone), mitochondrial OS	48.3	9.16
A0A0B2RNJ3	Aminoacylase-1B OS	38.4	5.82
A0A0R0IKX1	Uncharacterized protein OS	65.8	8.50
A0A0R0EQ02	Uncharacterized protein OS	58.9	6.61
I1JND9	Uncharacterized protein OS	82.3	6.65
A0A0B2RAS1	Uncharacterized protein OS	47.1	6.60
K7KBW1	Reticulon-like protein OS	52.6	8.75
I1N5K6	Uncharacterized protein OS	101.6	4.96
K7KMW8	Uncharacterized protein OS	38.9	5.57
A0A0B2NTU6	ABC transporter F family member 1 OS	65.4	6.46
A0A0B2QLX0	Pleiotropic drug resistance protein 1 OS	160.6	7.34
A0A0B2SPA1	Acetate--CoA ligase ACS, chloroplastic/glyoxysomal OS	77.8	5.73
K7MBG2	Uncharacterized protein OS	65.7	7.49
K7L7F4	Uncharacterized protein OS	56.6	8.50
A0A0B2QBN6	Clathrin interactor EPSIN 1 OS	60.6	5.08
A0A0B2QD67	Histone deacetylase 8 OS	41.2	5.88
A0A0R0JPS9	Uncharacterized protein OS	106.1	6.76
Q14SM9	Asparagine synthetase, type III OS	64.2	6.49
A0A0R0EU58	Uncharacterized protein (Fragment) OS	86.1	5.55
A0A0R0EZX1	Uncharacterized protein OS	51.4	5.03
I1KUU6	Transmembrane 9 superfamily member OS	73.6	8.22
I1LST9	Uncharacterized protein OS	66.7	6.29
A0A0R0JKH0	Transmembrane 9 superfamily member OS	62.7	7.85
A0A0B2QZZ7	Leukotriene A-4 hydrolase OS	68.6	5.81
K7LXV4	Uncharacterized protein OS	45.2	8.92
I1KIH3	Uncharacterized protein OS	50.6	6.44
A0A0R0JWW8	Uncharacterized protein (Fragment) OS	55.4	5.15
I1JRU2	Uncharacterized protein OS	288.1	6.79
A0A0B2Q2P4	Exocyst complex component 5 OS	88.4	5.33
I1MTB5	Uncharacterized protein OS	67.8	5.72
K7KTT9	Beta-adaptin-like protein OS	93.3	5.39
A0A0B2QEJ4	Uncharacterized protein OS	110.3	7.06
I1JMI9	Malic enzyme OS	68.9	6.02
I1LSZ2	Uncharacterized protein OS	89.8	5.22
A0A0B2PML2	Isoamylase 3, chloroplastic (Fragment) OS	81.1	6.27
A0A0B2PP12	Protein TPLATE OS	103.1	5.74
K7M896	Uncharacterized protein OS	144.5	6.37

K7MBV8	Calcium-transporting ATPase OS	106.1	7.81

Table S2. The list of DEPs in the sample 115/117 (MON87701×MON89788/A3525).

ID	Description	Sig. (Diff.115/117) times	
		up	down
A0A0B2Q5D1	Extended synaptotagmin-3 OS=Glycine soja GN=glysoja_011660 PE=4 SV=1 - [A0A0B2Q5D1_GLYSO]	3	0
A0A0B2QAS8	Glycine-rich RNA-binding protein 2, mitochondrial OS=Glycine soja GN=glysoja_006768 PE=4 SV=1 - [A0A0B2QAS8_GLYSO]	3	0
A0A0R0IBE3	Uncharacterized protein OS=Glycine max GN=GLYMA_09G214500 PE=4 SV=1 - [A0A0R0IBE3_SOYBN]	3	0
A0A0R4J681	Uncharacterized protein OS=Glycine max GN=GLYMA_20G233000 PE=4 SV=1 - [A0A0R4J681_SOYBN]	3	0
C6SX26	Putative uncharacterized protein OS=Glycine max PE=2 SV=1 - [C6SX26_SOYBN]	3	0
C6TAQ9	Uncharacterized protein OS=Glycine max GN=GLYMA_15G156700 PE=2 SV=1 - [C6TAQ9_SOYBN]	3	0
F7J077	Beta-conglycinin beta subunit OS=Glycine max GN=CG-beta-2 PE=4 SV=1 - [F7J077_SOYBN]	3	0
I1KS58	Uncharacterized protein OS=Glycine max GN=GLYMA_08G108000 PE=4 SV=1 - [I1KS58_SOYBN]	3	0
I1L5Z8	Uncharacterized protein OS=Glycine max GN=GLYMA_09G240300 PE=4 SV=1 - [I1L5Z8_SOYBN]	3	0
I1L8Q0	Lambda class glutathione S-transferase OS=Glycine max GN=GSTL3 PE=2 SV=1 - [I1L8Q0_SOYBN]	3	0
K7K8E5	Uncharacterized protein OS=Glycine max GN=GLYMA_02G147200 PE=4 SV=1 - [K7K8E5_SOYBN]	3	0
K7MZ42	Uncharacterized protein OS=Glycine max GN=GLYMA_19G186900 PE=4 SV=1 - [K7MZ42_SOYBN]	3	0
K7N254	Uncharacterized protein OS=Glycine max GN=GLYMA_20G075100 PE=4 SV=1 - [K7N254_SOYBN]	3	0
P45458	Malate synthase, glyoxysomal (Fragment) OS=Glycine max PE=2 SV=1 - [MASY_SOYBN]	3	0
Q04672	Sucrose-binding protein OS=Glycine max GN=SBP PE=1 SV=1 - [SBP_SOYBN]	3	0
Q71LY8	3-phosphoshikimate 1-carboxyvinyltransferase OS=Glycine max PE=3 SV=1 - [Q71LY8_SOYBN]	3	0
A0A0B2R0Z0	1-Cys peroxiredoxin OS=Glycine soja GN=glysoja_018783 PE=4 SV=1 - [A0A0B2R0Z0_GLYSO]	0	3
A0A0B2R3J3	Ribosomal protein L19 OS=Glycine soja GN=glysoja_038842 PE=3 SV=1 - [A0A0B2R3J3_GLYSO]	0	3
A0A0R0KRW0	Uncharacterized protein OS=Glycine max	0	3

	GN=GLYMA_03G163500 PE=4 SV=1 - [A0A0R0KRW0_SOYBN]		
A0A0R4J4U4	Uncharacterized protein OS=Glycine max GN=GLYMA_13G035200 PE=3 SV=1 - [A0A0R4J4U4_SOYBN]	0	3
C6SYM8	Uncharacterized protein OS=Glycine max GN=GLYMA_03G238700 PE=2 SV=1 - [C6SYM8_SOYBN]	0	3
C6T3U6	Ribosomal protein L19 OS=Glycine max GN=GLYMA_19G208200 PE=2 SV=1 - [C6T3U6_SOYBN]	0	3
I1KQW4	Uncharacterized protein OS=Glycine max PE=3 SV=2 - [I1KQW4_SOYBN]	0	3
O22378	Metallothionein-II protein OS=Glycine max GN=PGMPM19 PE=2 SV=1 - [O22378_SOYBN]	0	3
I1M0N4	Uncharacterized protein OS=Glycine max GN=GLYMA_13G192900 PE=4 SV=1 - [I1M0N4_SOYBN]	2	0
I1MTN1	Uncharacterized protein OS=Glycine max GN=GLYMA_17G094800 PE=4 SV=1 - [I1MTN1_SOYBN]	2	0
A0A0B2NW50	Lipoxygenase OS=Glycine soja GN=glysoja_009577 PE=3 SV=1 - [A0A0B2NW50_GLYSO]	2	0
A0A0B2Q1U6	Uncharacterized protein OS=Glycine soja GN=glysoja_025011 PE=4 SV=1 - [A0A0B2Q1U6_GLYSO]	2	0
A0A0B2R4V3	Uncharacterized protein OS=Glycine soja GN=glysoja_035516 PE=4 SV=1 - [A0A0B2R4V3_GLYSO]	2	0
A0A0B2SQB3	LysM domain-containing GPI-anchored protein 1 OS=Glycine soja GN=glysoja_018395 PE=4 SV=1 - [A0A0B2SQB3_GLYSO]	2	0
A0A0R0IKB3	Uncharacterized protein (Fragment) OS=Glycine max GN=GLYMA_09G2786001 PE=4 SV=1 - [A0A0R0IKB3_SOYBN]	2	0
I1MJD3	Cysteine synthase OS=Glycine max GN=GLYMA_15G262500 PE=3 SV=1 - [I1MJD3_SOYBN]	2	0
Q852U4	Glycinin A1bB2-784 OS=Glycine max PE=2 SV=1 - [Q852U4_SOYBN]	2	0
A0A0R0GGB7	Uncharacterized protein OS=Glycine max GN=GLYMA_14G223100 PE=4 SV=1 - [A0A0R0GGB7_SOYBN]	2	0
A0A0B2SIS3	Peroxidase OS=Glycine soja GN=glysoja_032062 PE=3 SV=1 - [A0A0B2SIS3_GLYSO]	2	0
A0A0B2PKZ5	Vacuolar-processing enzyme OS=Glycine soja GN=glysoja_017086 PE=4 SV=1 - [A0A0B2PKZ5_GLYSO]	2	0
Q9FQE8	Glutathione S-transferase OS=Glycine max GN=GSTU41 PE=1 SV=1 - [Q9FQE8_SOYBN]	2	0
A0A0R0H8Q8	Uncharacterized protein OS=Glycine max GN=GLYMA_13G345600 PE=3 SV=1 - [A0A0R0H8Q8_SOYBN]	2	0

I1JAQ7	Uncharacterized protein OS=Glycine max GN=GLYMA_01G235600 PE=4 SV=2 - [I1JAQ7_SOYBN]	2	0
I1KYS8	Uncharacterized protein OS=Glycine max GN=GLYMA_08G336000 PE=4 SV=1 - [I1KYS8_SOYBN]	2	0
K7L1Z5	Uncharacterized protein OS=Glycine max GN=GLYMA_07G157200 PE=4 SV=1 - [K7L1Z5_SOYBN]	2	0
I1M395	Uncharacterized protein OS=Glycine max GN=GLYMA_13G278000 PE=3 SV=1 - [I1M395_SOYBN]	2	0
Q39805	Dehydrin-like protein OS=Glycine max PE=2 SV=1 - [Q39805_SOYBN]	2	0
I1K441	Uncharacterized protein OS=Glycine max GN=GLYMA_05G163100 PE=4 SV=1 - [I1K441_SOYBN]	2	0
C6ZJY8	Serine hydroxymethyltransferase OS=Glycine max GN=GLYMA_08G274400 PE=2 SV=1 - [C6ZJY8_SOYBN]	2	0
I1KVR2	Uncharacterized protein OS=Glycine max GN=GLYMA_08G222100 PE=3 SV=1 - [I1KVR2_SOYBN]	2	0
I1L922	Uncharacterized protein OS=Glycine max GN=GLYMA_10G060200 PE=3 SV=2 - [I1L922_SOYBN]	2	0
Q70EM0	Dehydrin OS=Glycine max GN=lea-D-11 PE=3 SV=1 - [Q70EM0_SOYBN]	2	0
C6SWI0	Uncharacterized protein OS=Glycine max GN=GLYMA_02G053300 PE=2 SV=1 - [C6SWI0_SOYBN]	2	0
A0A0R0H1C4	Uncharacterized protein OS=Glycine max GN=GLYMA_12G031900 PE=4 SV=1 - [A0A0R0H1C4_SOYBN]	2	0
A0A0R0F0E0	Uncharacterized protein OS=Glycine max GN=GLYMA_18G153900 PE=4 SV=1 - [A0A0R0F0E0_SOYBN]	0	2
A0A0B2RQ67	Argininosuccinate synthase, chloroplastic OS=Glycine soja GN=glysoja_016736 PE=3 SV=1 - [A0A0B2RQ67_GLYSO]	0	2
A0A0B2RV43	Mitochondrial outer membrane protein porin 2 OS=Glycine soja GN=glysoja_003421 PE=4 SV=1 - [A0A0B2RV43_GLYSO]	0	2
A0A0B2PSC9	4-hydroxyphenylpyruvate dioxygenase OS=Glycine soja GN=glysoja_019953 PE=4 SV=1 - [A0A0B2PSC9_GLYSO]	0	2
I1J8Y8	Uncharacterized protein OS=Glycine max GN=GLYMA_01G178200 PE=3 SV=1 - [I1J8Y8_SOYBN]	0	2
I1M361	Uncharacterized protein OS=Glycine max GN=GLYMA_13G275000 PE=4 SV=1 - [I1M361_SOYBN]	0	2
I1NJ59	DHAR class glutathione S-transferase OS=Glycine max GN=DHAR4 PE=2 SV=1 - [I1NJ59_SOYBN]	0	2
A0A0B2S6T6	Glutamyl-tRNA reductase OS=Glycine soja GN=glysoja_009359 PE=3 SV=1 - [A0A0B2S6T6_GLYSO]	0	2
A0A0B2QKF8	Glucose and ribitol dehydrogenase OS=Glycine soja GN=glysoja_023775 PE=4 SV=1 - [A0A0B2QKF8_GLYSO]	0	2

P25273	Kunitz-type trypsin inhibitor KTI2 OS=Glycine max GN=KTI2 PE=2 SV=1 - [KTI2_SOYBN]	0	2
C6SXM8	Ribosomal protein L19 OS=Glycine max GN=GLYMA_03G216600 PE=2 SV=1 - [C6SXM8_SOYBN]	0	2

Table S3. The list of DEPs in the sample 114/117 (MON87705/A3525).

ID	Description	Sig. (Diff.114/117) times	
		up	down
A0A0B2Q5D1	Extended synaptotagmin-3 OS=Glycine soja GN=glysoja_011660 PE=4 SV=1 - [A0A0B2Q5D1_GLYSO]	3	0
I1KS58	Uncharacterized protein OS=Glycine max GN=GLYMA_08G108000 PE=4 SV=1 - [I1KS58_SOYBN]	3	0
I1M395	Uncharacterized protein OS=Glycine max GN=GLYMA_13G278000 PE=3 SV=1 - [I1M395_SOYBN]	3	0
C6SXY4	Uncharacterized protein OS=Glycine max GN=GLYMA_12G064400 PE=2 SV=1 - [C6SXY4_SOYBN]	3	0
A0A0B2NZI1	T-complex protein 1 subunit theta OS=Glycine soja GN=glysoja_000342 PE=3 SV=1 - [A0A0B2NZI1_GLYSO]	3	0
K7K8E5	Uncharacterized protein OS=Glycine max GN=GLYMA_02G147200 PE=4 SV=1 - [K7K8E5_SOYBN]	3	0
A0A0B2QAS8	Glycine-rich RNA-binding protein 2, mitochondrial OS=Glycine soja GN=glysoja_006768 PE=4 SV=1 - [A0A0B2QAS8_GLYSO]	3	0
P45458	Malate synthase, glyoxysomal (Fragment) OS=Glycine max PE=2 SV=1 - [MASY_SOYBN]	3	0
A0A0B2RG07	Calnexin like OS=Glycine soja GN=glysoja_012889 PE=3 SV=1 - [A0A0B2RG07_GLYSO]	3	0
I1NAI0	Uncharacterized protein OS=Glycine max GN=GLYMA_19G190400 PE=4 SV=2 - [I1NAI0_SOYBN]	3	0
I1K3Q1	Cysteine proteinase inhibitor OS=Glycine max GN=GLYMA_05G149800 PE=3 SV=1 - [I1K3Q1_SOYBN]	3	0
P62163	Calmodulin-2 OS=Glycine max GN=CAM-2 PE=1 SV=2 - [CALM2_SOYBN]	3	0
Q71LY8	3-phosphoshikimate 1-carboxyvinyltransferase OS=Glycine max PE=3 SV=1 - [Q71LY8_SOYBN]	3	0
K7MZ42	Uncharacterized protein OS=Glycine max GN=GLYMA_19G186900 PE=4 SV=1 - [K7MZ42_SOYBN]	3	0
I1KQW4	Uncharacterized protein OS=Glycine max PE=3 SV=2 - [I1KQW4_SOYBN]	0	3
C6SZX7	Glutathione peroxidase OS=Glycine max GN=GLYMA_01G219400 PE=2 SV=1 - [C6SZX7_SOYBN]	0	3
A0A0B2R4V3	Uncharacterized protein OS=Glycine soja GN=glysoja_035516 PE=4 SV=1 - [A0A0B2R4V3_GLYSO]	2	0
A0A0R0HH51	Uncharacterized protein OS=Glycine max	2	0

	GN=GLYMA_12G112900 PE=4 SV=1 - [A0A0R0HH51_SOYBN]		
I1L8Q0	Lambda class glutathione S-transferase OS=Glycine max GN=GSTL3 PE=2 SV=1 - [I1L8Q0_SOYBN]	2	0
I1M0N4	Uncharacterized protein OS=Glycine max GN=GLYMA_13G192900 PE=4 SV=1 - [I1M0N4_SOYBN]	2	0
I1LUY8	Uncharacterized protein OS=Glycine max GN=GLYMA_12G222400 PE=4 SV=1 - [I1LUY8_SOYBN]	2	0
A0A0R0IIZ3	Uncharacterized protein OS=Glycine max GN=GLYMA_09G178100 PE=3 SV=1 - [A0A0R0IIZ3_SOYBN]	2	0
I1M138	Uncharacterized protein OS=Glycine max GN=GLYMA_13G208000 PE=3 SV=1 - [I1M138_SOYBN]	2	0
I1JR89	Uncharacterized protein OS=Glycine max GN=GLYMA_03G234400 PE=4 SV=1 - [I1JR89_SOYBN]	2	0
A0A0B2SQP4	Clustered mitochondria protein homolog OS=Glycine soja GN=glysoja_031237 PE=3 SV=1 - [A0A0B2SQP4_GLYSO]	2	0
C6SYZ8	Uncharacterized protein OS=Glycine max GN=GLYMA_09G024000 PE=2 SV=1 - [C6SYZ8_SOYBN]	2	0
A0A0B2QYK0	U-box domain-containing protein 72 OS=Glycine soja GN=glysoja_028724 PE=4 SV=1 - [A0A0B2QYK0_GLYSO]	2	0
B1Q2X5	Protein disulfide-isomerase OS=Glycine max GN=PDIL-2 PE=2 SV=1 - [B1Q2X5_SOYBN]	2	0
Q852U4	Glycinin A1bB2-784 OS=Glycine max PE=2 SV=1 - [Q852U4_SOYBN]	2	0
A0A0R0J9Y0	Uncharacterized protein OS=Glycine max GN=GLYMA_07G195900 PE=4 SV=1 - [A0A0R0J9Y0_SOYBN]	2	0
K7N254	Uncharacterized protein OS=Glycine max GN=GLYMA_20G075100 PE=4 SV=1 - [K7N254_SOYBN]	2	0
C6SVR8	Uncharacterized protein OS=Glycine max GN=GLYMA_11G158500 PE=2 SV=1 - [C6SVR8_SOYBN]	2	0
A0A0R0KGJ7	Uncharacterized protein OS=Glycine max GN=GLYMA_03G090000 PE=4 SV=1 - [A0A0R0KGJ7_SOYBN]	2	0
C6TAQ9	Uncharacterized protein OS=Glycine max GN=GLYMA_15G156700 PE=2 SV=1 - [C6TAQ9_SOYBN]	2	0
I1MJD3	Cysteine synthase OS=Glycine max GN=GLYMA_15G262500 PE=3 SV=1 - [I1MJD3_SOYBN]	2	0
C6TAR9	Putative uncharacterized protein OS=Glycine max PE=2 SV=1 - [C6TAR9_SOYBN]	2	0
A0A0R0IBE3	Uncharacterized protein OS=Glycine max GN=GLYMA_09G214500 PE=4 SV=1 - [A0A0R0IBE3_SOYBN]	2	0
A0A0R0ICH7	Uncharacterized protein OS=Glycine max	2	0

	GN=GLYMA_09G235900 PE=4 SV=1 - [A0A0R0ICH7_SOYBN]		
A0A0B2PKL0	Defensin-like protein (Fragment) OS=Glycine soja GN=glysoja_011949 PE=3 SV=1 - [A0A0B2PKL0_GLYSO]	2	0
K7L7I8	Uncharacterized protein OS=Glycine max PE=4 SV=1 - [K7L7I8_SOYBN]	2	0
A0A0B2SQB3	LysM domain-containing GPI-anchored protein 1 OS=Glycine soja GN=glysoja_018395 PE=4 SV=1 - [A0A0B2SQB3_GLYSO]	2	0
A0A0R0E916	Uncharacterized protein OS=Glycine max GN=GLYMA_20G089400 PE=4 SV=1 - [A0A0R0E916_SOYBN]	2	0
I1JLC8	Protein SLE2 OS=Glycine max GN=SLE2 PE=2 SV=1 - [SLE2_SOYBN]	2	0
I1K8S6	Uncharacterized protein OS=Glycine max GN=GLYMA_06G066400 PE=4 SV=1 - [I1K8S6_SOYBN]	2	0
A0A0B2Q1T9	Tryptophan synthase beta chain 2 OS=Glycine soja GN=glysoja_046993 PE=3 SV=1 - [A0A0B2Q1T9_GLYSO]	2	0
K7LHL1	Uncharacterized protein OS=Glycine max PE=4 SV=1 - [K7LHL1_SOYBN]	2	0
A0A0R0IKB3	Uncharacterized protein (Fragment) OS=Glycine max GN=GLYMA_09G2786001 PE=4 SV=1 - [A0A0R0IKB3_SOYBN]	2	0
C6TFC7	Putative uncharacterized protein OS=Glycine max PE=2 SV=1 - [C6TFC7_SOYBN]	2	0
I1JC44	Uncharacterized protein OS=Glycine max GN=GLYMA_02G036300 PE=4 SV=1 - [I1JC44_SOYBN]	2	0
I1MMK6	Uncharacterized protein OS=Glycine max GN=GLYMA_16G103900 PE=4 SV=1 - [I1MMK6_SOYBN]	2	0
A0A097PK35	Pollen Ole e 1 allergen and extensin family protein (Fragment) OS=Glycine max PE=2 SV=1 - [A0A097PK35_SOYBN]	2	0
I1L7J2	Uncharacterized protein OS=Glycine max GN=GLYMA_10G007800 PE=4 SV=1 - [I1L7J2_SOYBN]	2	0
A0A0B2Q1U6	Uncharacterized protein OS=Glycine soja GN=glysoja_025011 PE=4 SV=1 - [A0A0B2Q1U6_GLYSO]	2	0
I1MTN1	Uncharacterized protein OS=Glycine max GN=GLYMA_17G094800 PE=4 SV=1 - [I1MTN1_SOYBN]	2	0
I1MEH3	Uncharacterized protein OS=Glycine max GN=GLYMA_15G072900 PE=4 SV=1 - [I1MEH3_SOYBN]	0	2
O22378	Metallothionein-II protein OS=Glycine max GN=PGMPM19 PE=2 SV=1 - [O22378_SOYBN]	0	2
Q9ZS21	Lactoylglutathione lyase OS=Glycine max GN=GLXI PE=1 SV=1 - [LGUL_SOYBN]	0	2
A0A0B2RM77	Cysteine proteinase inhibitor OS=Glycine soja GN=glysoja_009953 PE=3 SV=1 - [A0A0B2RM77_GLYSO]	0	2

A0A0B2R2C7	C-1-tetrahydrofolate synthase, cytoplasmic OS=Glycine soja GN=glysoja_038828 PE=3 SV=1 - [A0A0B2R2C7_GLYSO]	0	2
A0A0B2RV43	Mitochondrial outer membrane protein porin 2 OS=Glycine soja GN=glysoja_003421 PE=4 SV=1 - [A0A0B2RV43_GLYSO]	0	2
A0A0B2NW50	Lipoxygenase OS=Glycine soja GN=glysoja_009577 PE=3 SV=1 - [A0A0B2NW50_GLYSO]	0	2
C6SWE8	Superoxide dismutase [Cu-Zn] OS=Glycine max PE=2 SV=1 - [C6SWE8_SOYBN]	0	2
I1KSE0	Uncharacterized protein OS=Glycine max GN=GLYMA_08G115800 PE=3 SV=1 - [I1KSE0_SOYBN]	0	2
C6TKJ1	Putative uncharacterized protein OS=Glycine max PE=2 SV=1 - [C6TKJ1_SOYBN]	0	2
C6T9W4	Putative Fe(II)-and 2-oxoglutaratedependent dioxygenase OS=Glycine max GN=F6'H1 PE=2 SV=1 - [C6T9W4_SOYBN]	0	2
A0A0R0F0E0	Uncharacterized protein OS=Glycine max GN=GLYMA_18G153900 PE=4 SV=1 - [A0A0R0F0E0_SOYBN]	0	2
A0A0B2RQ67	Argininosuccinate synthase, chloroplastic OS=Glycine soja GN=glysoja_016736 PE=3 SV=1 - [A0A0B2RQ67_GLYSO]	0	2

Table S4. The list of DEPs in the sample 118/117 (MON87708/A3525).

ID	Description	Sig. (Diff.118/117) times	
		up	down
A0A0R0IIZ3	Uncharacterized protein OS=Glycine max GN=GLYMA_09G178100 PE=3 SV=1 - [A0A0R0IIZ3_SOYBN]	0	3
A0A0B2SBV9	AP-4 complex subunit epsilon OS=Glycine soja GN=glysoja_003509 PE=4 SV=1 - [A0A0B2SBV9_GLYSO]	2	0
I1M0N4	Uncharacterized protein OS=Glycine max GN=GLYMA_13G192900 PE=4 SV=1 - [I1M0N4_SOYBN]	2	0
I1M395	Uncharacterized protein OS=Glycine max GN=GLYMA_13G278000 PE=3 SV=1 - [I1M395_SOYBN]	2	0
A0A0B2PP86	Trypsin inhibitor A OS=Glycine soja GN=glysoja_026645 PE=4 SV=1 - [A0A0B2PP86_GLYSO]	2	0
A0A0B2Q5P0	Uncharacterized protein OS=Glycine soja GN=glysoja_011760 PE=4 SV=1 - [A0A0B2Q5P0_GLYSO]	2	0
C6SW58	Putative uncharacterized protein (Fragment) OS=Glycine max PE=2 SV=1 - [C6SW58_SOYBN]	2	0
I1MTN1	Uncharacterized protein OS=Glycine max GN=GLYMA_17G094800 PE=4 SV=1 - [I1MTN1_SOYBN]	2	0
A0A0R0H1C4	Uncharacterized protein OS=Glycine max GN=GLYMA_12G031900 PE=4 SV=1 - [A0A0R0H1C4_SOYBN]	2	0

C6TB95	Uncharacterized protein OS=Glycine max GN=GLYMA_01G218900 PE=2 SV=1 - [C6TB95_SOYBN]	0	2
I1LM67	Uncharacterized protein OS=Glycine max GN=GLYMA_13G153000 PE=4 SV=1 - [I1LM67_SOYBN]	0	2
I1J8Y8	Uncharacterized protein OS=Glycine max GN=GLYMA_01G178200 PE=3 SV=1 - [I1J8Y8_SOYBN]	0	2
A0A0R0F0E0	Uncharacterized protein OS=Glycine max GN=GLYMA_18G153900 PE=4 SV=1 - [A0A0R0F0E0_SOYBN]	0	2

Table S5. The list of DEPs in the sample 116/119 (FG72/FG72-JACK).

ID	Description	Sig. (Diff.116/119) times	
		up	down
A0A0R0IIZ3	Uncharacterized protein OS=Glycine max GN=GLYMA_09G178100 PE=3 SV=1 - [A0A0R0IIZ3_SOYBN]	3	0
A0A0R0HKE3	Uncharacterized protein OS=Glycine max GN=GLYMA_11G214800 PE=4 SV=1 - [A0A0R0HKE3_SOYBN]	0	3
K7K9D0	Uncharacterized protein OS=Glycine max GN=GLYMA_02G184600 PE=4 SV=1 - [K7K9D0_SOYBN]	0	3
A0A0B2PMR9	Em-like protein GEA6 OS=Glycine soja GN=glysoja_045231 PE=4 SV=1 - [A0A0B2PMR9_GLYSO]	2	0
K7MGG1	Uncharacterized protein OS=Glycine max GN=GLYMA_16G100400 PE=3 SV=1 - [K7MGG1_SOYBN]	2	0
I1LCV0	Uncharacterized protein OS=Glycine max GN=GLYMA_10G205900 PE=4 SV=1 - [I1LCV0_SOYBN]	2	0
A0A0B2P4D2	Glycinin G3 OS=Glycine soja GN=glysoja_002527 PE=4 SV=1 - [A0A0B2P4D2_GLYSO]	2	0
C6TD56	Glyceraldehyde-3-phosphate dehydrogenase OS=Glycine max GN=GLYMA_11G247600 PE=2 SV=1 - [C6TD56_SOYBN]	2	0
I1JDH6	Uncharacterized protein OS=Glycine max GN=GLYMA_02G085300 PE=3 SV=1 - [I1JDH6_SOYBN]	2	0
I1J7U9	3-phosphoshikimate 1-carboxyvinyltransferase OS=Glycine max GN=GLYMA_01G139600 PE=3 SV=1 - [I1J7U9_SOYBN]	2	0
A0A0R0JL19	Uncharacterized protein OS=Glycine max GN=GLYMA_06G122600 PE=3 SV=1 - [A0A0R0JL19_SOYBN]	2	0
K7L817	40S ribosomal protein S12 OS=Glycine max GN=GLYMA_08G217700 PE=3 SV=1 - [K7L817_SOYBN]	2	0
I1KVR2	Uncharacterized protein OS=Glycine max GN=GLYMA_08G222100 PE=3 SV=1 - [I1KVR2_SOYBN]	2	0
A0A0B2PNU6	Heat shock protein 83 OS=Glycine soja GN=glysoja_029069 PE=3 SV=1 - [A0A0B2PNU6_GLYSO]	0	2
I1NF81	Uncharacterized protein OS=Glycine max	0	2

	GN=GLYMA_20G106300 PE=4 SV=2 - [I1NF81_SOYBN]		
I1KGH6	Uncharacterized protein OS=Glycine max GN=GLYMA_07G014400 PE=3 SV=1 - [I1KGH6_SOYBN]	0	2
I1MU68	Uncharacterized protein OS=Glycine max GN=GLYMA_17G112500 PE=4 SV=2 - [I1MU68_SOYBN]	0	2
C6T8J1	Uncharacterized protein OS=Glycine max GN=GLYMA_13G098500 PE=2 SV=1 - [C6T8J1_SOYBN]	0	2
A0A0B2P1M7	U-box domain-containing protein 35 (Fragment) OS=Glycine soja GN=glysoja_009585 PE=4 SV=1 - [A0A0B2P1M7_GLYSO]	0	2
A0A0B2S8C8	Uncharacterized protein OS=Glycine soja GN=glysoja_007471 PE=4 SV=1 - [A0A0B2S8C8_GLYSO]	0	2
I1L922	Uncharacterized protein OS=Glycine max GN=GLYMA_10G060200 PE=3 SV=2 - [I1L922_SOYBN]	0	2
Q70EM0	Dehydrin OS=Glycine max GN=lea-D-11 PE=3 SV=1 - [Q70EM0_SOYBN]	0	2

Table S6. The list of DEPs in the sample 115/114 (MON87701×MON89788/MON87705).

ID	Description	Sig. (Diff.115/114) times	
		up	down
C6SX26	Putative uncharacterized protein OS=Glycine max PE=2 SV=1 - [C6SX26_SOYBN]	3	0
I1L8Q0	Lambda class glutathione S-transferase OS=Glycine max GN=GSTL3 PE=2 SV=1 - [I1L8Q0_SOYBN]	3	0
F7J077	Beta-conglycinin beta subunit OS=Glycine max GN=CG-beta-2 PE=4 SV=1 - [F7J077_SOYBN]	3	0
A0A0R4J681	Uncharacterized protein OS=Glycine max GN=GLYMA_20G233000 PE=4 SV=1 - [A0A0R4J681_SOYBN]	3	0
Q04672	Sucrose-binding protein OS=Glycine max GN=SBP PE=1 SV=1 - [SBP_SOYBN]	3	0
C6TFC1	Non-specific lipid-transfer protein OS=Glycine max GN=GLYMA_03G040400 PE=2 SV=1 - [C6TFC1_SOYBN]	3	0
I1L5Z8	Uncharacterized protein OS=Glycine max GN=GLYMA_09G240300 PE=4 SV=1 - [I1L5Z8_SOYBN]	3	0
K7N4A7	Uncharacterized protein OS=Glycine max PE=3 SV=1 - [K7N4A7_SOYBN]	3	0
A0A0R4J460	Uncharacterized protein OS=Glycine max GN=GLYMA_09G231200 PE=4 SV=1 - [A0A0R4J460_SOYBN]	2	0
A0A0B2PKZ5	Vacuolar-processing enzyme OS=Glycine soja GN=glysoja_017086 PE=4 SV=1 - [A0A0B2PKZ5_GLYSO]	2	0
C6TC98	Putative uncharacterized protein (Fragment) OS=Glycine max PE=2 SV=1 - [C6TC98_SOYBN]	2	0
Q9FQE8	Glutathione S-transferase OS=Glycine max GN=GSTU41 PE=1 SV=1 - [Q9FQE8_SOYBN]	2	0

I1KYS8	Uncharacterized protein OS=Glycine max GN=GLYMA_08G336000 PE=4 SV=1 - [I1KYS8_SOYBN]	2	0
K7L1Z5	Uncharacterized protein OS=Glycine max GN=GLYMA_07G157200 PE=4 SV=1 - [K7L1Z5_SOYBN]	2	0
C6TAQ9	Uncharacterized protein OS=Glycine max GN=GLYMA_15G156700 PE=2 SV=1 - [C6TAQ9_SOYBN]	2	0
A0A0B2NW50	Lipoxygenase OS=Glycine soja GN=glysoja_009577 PE=3 SV=1 - [A0A0B2NW50_GLYSO]	2	0
C6SWE8	Superoxide dismutase [Cu-Zn] OS=Glycine max PE=2 SV=1 - [C6SWE8_SOYBN]	2	0
K7K8E5	Uncharacterized protein OS=Glycine max GN=GLYMA_02G147200 PE=4 SV=1 - [K7K8E5_SOYBN]	2	0
C6T7Q4	Putative uncharacterized protein OS=Glycine max PE=2 SV=1 - [C6T7Q4_SOYBN]	2	0
K7MNF4	Uncharacterized protein OS=Glycine max GN=GLYMA_17G227500 PE=3 SV=1 - [K7MNF4_SOYBN]	2	0
A0A0B2PDU7	Peptide methionine sulfoxide reductase OS=Glycine soja GN=glysoja_038717 PE=3 SV=1 - [A0A0B2PDU7_GLYSO]	2	0
C6SVI7	Uncharacterized protein OS=Glycine max GN=GLYMA_10G242000 PE=2 SV=1 - [C6SVI7_SOYBN]	2	0
A0A0B2QUL3	Tropinone reductase like OS=Glycine soja GN=glysoja_010147 PE=4 SV=1 - [A0A0B2QUL3_GLYSO]	2	0
I1MBN4	Uncharacterized protein OS=Glycine max GN=GLYMA_14G207600 PE=4 SV=1 - [I1MBN4_SOYBN]	2	0
I1MEF8	Uncharacterized protein OS=Glycine max GN=GLYMA_15G072100 PE=4 SV=1 - [I1MEF8_SOYBN]	2	0
C6T5V2	Cysteine proteinase inhibitor (Fragment) OS=Glycine max PE=2 SV=1 - [C6T5V2_SOYBN]	2	0
C6T9W4	Putative Fe(II)-and 2-oxoglutaratedependent dioxygenase OS=Glycine max GN=F6'H1 PE=2 SV=1 - [C6T9W4_SOYBN]	2	0
I1KVR2	Uncharacterized protein OS=Glycine max GN=GLYMA_08G222100 PE=3 SV=1 - [I1KVR2_SOYBN]	2	0
Q70EM0	Dehydrin OS=Glycine max GN=lea-D-11 PE=3 SV=1 - [Q70EM0_SOYBN]	2	0
C6SVK3	Uncharacterized protein OS=Glycine max GN=GLYMA_13G222200 PE=2 SV=1 - [C6SVK3_SOYBN]	2	0
C6THU0	Uncharacterized protein OS=Glycine max GN=GLYMA_13G182000 PE=2 SV=1 - [C6THU0_SOYBN]	2	0
A0A0R0G3L8	Phospho-2-dehydro-3-deoxyheptonate aldolase OS=Glycine max GN=GLYMA_15G054700 PE=3 SV=1 - [A0A0R0G3L8_SOYBN]	2	0
A0A0B2P7Y1	Ran-binding protein 1 like b OS=Glycine soja GN=glysoja_046443 PE=4 SV=1 - [A0A0B2P7Y1_GLYSO]	0	2
A0A0B2SQP4	Clustered mitochondria protein homolog OS=Glycine soja	0	2

	GN=glysoja_031237 PE=3 SV=1 - [A0A0B2SQP4_GLYSO]		
C6SVR8	Uncharacterized protein OS=Glycine max GN=GLYMA_11G158500 PE=2 SV=1 - [C6SVR8_SOYBN]	0	2
I1M395	Uncharacterized protein OS=Glycine max GN=GLYMA_13G278000 PE=3 SV=1 - [I1M395_SOYBN]	0	2
A0A0B2PKL0	Defensin-like protein (Fragment) OS=Glycine soja GN=glysoja_011949 PE=3 SV=1 - [A0A0B2PKL0_GLYSO]	0	2
C6SYM8	Uncharacterized protein OS=Glycine max GN=GLYMA_03G238700 PE=2 SV=1 - [C6SYM8_SOYBN]	0	2
I1M361	Uncharacterized protein OS=Glycine max GN=GLYMA_13G275000 PE=4 SV=1 - [I1M361_SOYBN]	0	2
A0A0R0H152	Uncharacterized protein OS=Glycine max GN=GLYMA_12G046000 PE=4 SV=1 - [A0A0R0H152_SOYBN]	0	2
I1JLC8	Protein SLE2 OS=Glycine max GN=SLE2 PE=2 SV=1 - [SLE2_SOYBN]	0	2
A0A0B2Q1T9	Tryptophan synthase beta chain 2 OS=Glycine soja GN=glysoja_046993 PE=3 SV=1 - [A0A0B2Q1T9_GLYSO]	0	2
C6T3U6	Ribosomal protein L19 OS=Glycine max GN=GLYMA_19G208200 PE=2 SV=1 - [C6T3U6_SOYBN]	0	2
I1K3Q1	Cysteine proteinase inhibitor OS=Glycine max GN=GLYMA_05G149800 PE=3 SV=1 - [I1K3Q1_SOYBN]	0	2
A0A0R0JL19	Uncharacterized protein OS=Glycine max GN=GLYMA_06G122600 PE=3 SV=1 - [A0A0R0JL19_SOYBN]	0	2
A0A0B2R0Z0	1-Cys peroxiredoxin OS=Glycine soja GN=glysoja_018783 PE=4 SV=1 - [A0A0B2R0Z0_GLYSO]	0	2
K7LHL1	Uncharacterized protein OS=Glycine max PE=4 SV=1 - [K7LHL1_SOYBN]	0	2
A0A0B2QKF8	Glucose and ribitol dehydrogenase OS=Glycine soja GN=glysoja_023775 PE=4 SV=1 - [A0A0B2QKF8_GLYSO]	0	2
K7MZ42	Uncharacterized protein OS=Glycine max GN=GLYMA_19G186900 PE=4 SV=1 - [K7MZ42_SOYBN]	0	2
I1N583	Uncharacterized protein OS=Glycine max GN=GLYMA_18G292300 PE=4 SV=1 - [I1N583_SOYBN]	0	3
A0A0R4J4U4	Uncharacterized protein OS=Glycine max GN=GLYMA_13G035200 PE=3 SV=1 - [A0A0R4J4U4_SOYBN]	0	3
P62163	Calmodulin-2 OS=Glycine max GN=CAM-2 PE=1 SV=2 - [CALM2_SOYBN]	0	3

Table S7. The list of DEPs in the sample 115/118 (MON87701×MON89788/MON87708).

ID	Description	Sig. (Diff.115/118) times	
		up	down

C6SX26	Putative uncharacterized protein OS=Glycine max PE=2 SV=1 - [C6SX26_SOYBN]	3	0
I1L8Q0	Lambda class glutathione S-transferase OS=Glycine max GN=GSTL3 PE=2 SV=1 - [I1L8Q0_SOYBN]	3	0
A0A0R0IIZ3	Uncharacterized protein OS=Glycine max GN=GLYMA_09G178100 PE=3 SV=1 - [A0A0R0IIZ3_SOYBN]	3	0
F7J077	Beta-conglycinin beta subunit OS=Glycine max GN=CG-beta-2 PE=4 SV=1 - [F7J077_SOYBN]	3	0
Q9FQE8	Glutathione S-transferase OS=Glycine max GN=GSTU41 PE=1 SV=1 - [Q9FQE8_SOYBN]	3	0
C6TAQ9	Uncharacterized protein OS=Glycine max GN=GLYMA_15G156700 PE=2 SV=1 - [C6TAQ9_SOYBN]	3	0
I1LM67	Uncharacterized protein OS=Glycine max GN=GLYMA_13G153000 PE=4 SV=1 - [I1LM67_SOYBN]	3	0
A0A0B2QAS8	Glycine-rich RNA-binding protein 2, mitochondrial OS=Glycine soja GN=glysoja_006768 PE=4 SV=1 - [A0A0B2QAS8_GLYSO]	3	0
I1K441	Uncharacterized protein OS=Glycine max GN=GLYMA_05G163100 PE=4 SV=1 - [I1K441_SOYBN]	3	0
A0A0R4J681	Uncharacterized protein OS=Glycine max GN=GLYMA_20G233000 PE=4 SV=1 - [A0A0R4J681_SOYBN]	3	0
Q04672	Sucrose-binding protein OS=Glycine max GN=SBP PE=1 SV=1 - [SBP_SOYBN]	3	0
I1L5Z8	Uncharacterized protein OS=Glycine max GN=GLYMA_09G240300 PE=4 SV=1 - [I1L5Z8_SOYBN]	3	0
K7N4A7	Uncharacterized protein OS=Glycine max PE=3 SV=1 - [K7N4A7_SOYBN]	3	0
Q71LY8	3-phosphoshikimate 1-carboxyvinyltransferase OS=Glycine max PE=3 SV=1 - [Q71LY8_SOYBN]	3	0
K7MZ42	Uncharacterized protein OS=Glycine max GN=GLYMA_19G186900 PE=4 SV=1 - [K7MZ42_SOYBN]	3	0
A0A0R0GGB7	Uncharacterized protein OS=Glycine max GN=GLYMA_14G223100 PE=4 SV=1 - [A0A0R0GGB7_SOYBN]	2	0
A0A0B2R4V3	Uncharacterized protein OS=Glycine soja GN=glysoja_035516 PE=4 SV=1 - [A0A0B2R4V3_GLYSO]	2	0
A0A0B2PKZ5	Vacuolar-processing enzyme OS=Glycine soja GN=glysoja_017086 PE=4 SV=1 - [A0A0B2PKZ5_GLYSO]	2	0
Q852U4	Glycinin A1bB2-784 OS=Glycine max PE=2 SV=1 - [Q852U4_SOYBN]	2	0
I1JAQ7	Uncharacterized protein OS=Glycine max GN=GLYMA_01G235600 PE=4 SV=2 - [I1JAQ7_SOYBN]	2	0
I1KS58	Uncharacterized protein OS=Glycine max GN=GLYMA_08G108000 PE=4 SV=1 - [I1KS58_SOYBN]	2	0
I1KYS8	Uncharacterized protein OS=Glycine max	2	0

	GN=GLYMA_08G336000 PE=4 SV=1 - [I1KYS8_SOYBN]		
K7L1Z5	Uncharacterized protein OS=Glycine max GN=GLYMA_07G157200 PE=4 SV=1 - [K7L1Z5_SOYBN]	2	0
Q39805	Dehydrin-like protein OS=Glycine max PE=2 SV=1 - [Q39805_SOYBN]	2	0
A0A0B2NW50	Lipoxygenase OS=Glycine soja GN=glysoja_009577 PE=3 SV=1 - [A0A0B2NW50_GLYSO]	2	0
I1LCV0	Uncharacterized protein OS=Glycine max GN=GLYMA_10G205900 PE=4 SV=1 - [I1LCV0_SOYBN]	2	0
I1MJD3	Cysteine synthase OS=Glycine max GN=GLYMA_15G262500 PE=3 SV=1 - [I1MJD3_SOYBN]	2	0
A0A0R0IBE3	Uncharacterized protein OS=Glycine max GN=GLYMA_09G214500 PE=4 SV=1 - [A0A0R0IBE3_SOYBN]	2	0
I1N5I8	Oleosin OS=Glycine max GN=GLYMA_19G004800 PE=3 SV=1 - [I1N5I8_SOYBN]	2	0
I1J8A4	Thioredoxin reductase OS=Glycine max GN=GLYMA_01G156600 PE=3 SV=2 - [I1J8A4_SOYBN]	2	0
K7K8E5	Uncharacterized protein OS=Glycine max GN=GLYMA_02G147200 PE=4 SV=1 - [K7K8E5_SOYBN]	2	0
P45458	Malate synthase, glyoxysomal (Fragment) OS=Glycine max PE=2 SV=1 - [MASY_SOYBN]	2	0
I1MBF5	Uncharacterized protein OS=Glycine max GN=GLYMA_14G198000 PE=4 SV=1 - [I1MBF5_SOYBN]	2	0
C6T7Q4	Putative uncharacterized protein OS=Glycine max PE=2 SV=1 - [C6T7Q4_SOYBN]	2	0
A0A0B2QUL3	Tropinone reductase like OS=Glycine soja GN=glysoja_010147 PE=4 SV=1 - [A0A0B2QUL3_GLYSO]	2	0
I1MBN4	Uncharacterized protein OS=Glycine max GN=GLYMA_14G207600 PE=4 SV=1 - [I1MBN4_SOYBN]	2	0
A0A0R0ELE0	Pyrroline-5-carboxylate reductase OS=Glycine max GN=GLYMA_19G131500 PE=3 SV=1 - [A0A0R0ELE0_SOYBN]	2	0
A0A0R0IKB3	Uncharacterized protein (Fragment) OS=Glycine max GN=GLYMA_09G2786001 PE=4 SV=1 - [A0A0R0IKB3_SOYBN]	2	0
I1KVR2	Uncharacterized protein OS=Glycine max GN=GLYMA_08G222100 PE=3 SV=1 - [I1KVR2_SOYBN]	2	0
I1L922	Uncharacterized protein OS=Glycine max GN=GLYMA_10G060200 PE=3 SV=2 - [I1L922_SOYBN]	2	0
Q70EM0	Dehydrin OS=Glycine max GN=lea-D-11 PE=3 SV=1 - [Q70EM0_SOYBN]	2	0
A0A0B2S2I4	Nicalin-1 (Fragment) OS=Glycine soja GN=glysoja_008163 PE=4 SV=1 - [A0A0B2S2I4_GLYSO]	2	0

C6THU0	Uncharacterized protein OS=Glycine max GN=GLYMA_13G182000 PE=2 SV=1 - [C6THU0_SOYBN]	2	0
A0A097PK35	Pollen Ole e 1 allergen and extensin family protein (Fragment) OS=Glycine max PE=2 SV=1 - [A0A097PK35_SOYBN]	2	0
A0A0B2Q1U6	Uncharacterized protein OS=Glycine soja GN=glysoja_025011 PE=4 SV=1 - [A0A0B2Q1U6_GLYSO]	2	0
I1JEL1	Uncharacterized protein OS=Glycine max GN=GLYMA_02G124000 PE=4 SV=1 - [I1JEL1_SOYBN]	2	0
A0A0B2RRQ5	Golgin candidate 6 OS=Glycine soja GN=glysoja_049179 PE=4 SV=1 - [A0A0B2RRQ5_GLYSO]	2	0
C6SXP1	Putative uncharacterized protein OS=Glycine max PE=2 SV=1 - [C6SXP1_SOYBN]	0	2
I1JYG8	Uncharacterized protein OS=Glycine max PE=4 SV=1 - [I1JYG8_SOYBN]	0	2
A0A0B2SSB2	26.5 kDa heat shock protein, mitochondrial OS=Glycine soja GN=glysoja_029181 PE=3 SV=1 - [A0A0B2SSB2_GLYSO]	0	2
I1MKY2	Uncharacterized protein OS=Glycine max GN=GLYMA_16G037800 PE=4 SV=1 - [I1MKY2_SOYBN]	0	2
C6SYM8	Uncharacterized protein OS=Glycine max GN=GLYMA_03G238700 PE=2 SV=1 - [C6SYM8_SOYBN]	0	2
I1M361	Uncharacterized protein OS=Glycine max GN=GLYMA_13G275000 PE=4 SV=1 - [I1M361_SOYBN]	0	2
A0A0B2P1M7	U-box domain-containing protein 35 (Fragment) OS=Glycine soja GN=glysoja_009585 PE=4 SV=1 - [A0A0B2P1M7_GLYSO]	0	2
I1NJ59	DHAR class glutathione S-transferase OS=Glycine max GN=DHAR4 PE=2 SV=1 - [I1NJ59_SOYBN]	0	2
A0A0B2S8C8	Uncharacterized protein OS=Glycine soja GN=glysoja_007471 PE=4 SV=1 - [A0A0B2S8C8_GLYSO]	0	2
A0A0B2PP86	Trypsin inhibitor A OS=Glycine soja GN=glysoja_026645 PE=4 SV=1 - [A0A0B2PP86_GLYSO]	0	2
A0A0B2RHP8	Membrane steroid-binding protein 2 OS=Glycine soja GN=glysoja_029638 PE=3 SV=1 - [A0A0B2RHP8_GLYSO]	0	2
C6T3U6	Ribosomal protein L19 OS=Glycine max GN=GLYMA_19G208200 PE=2 SV=1 - [C6T3U6_SOYBN]	0	2
A0A0B2R0Z0	1-Cys peroxiredoxin OS=Glycine soja GN=glysoja_018783 PE=4 SV=1 - [A0A0B2R0Z0_GLYSO]	0	2
A0A0B2QKF8	Glucose and ribitol dehydrogenase OS=Glycine soja GN=glysoja_023775 PE=4 SV=1 - [A0A0B2QKF8_GLYSO]	0	2
A0A0B2R290	Putative glutathione S-transferase OS=Glycine soja GN=glysoja_025575 PE=4 SV=1 - [A0A0B2R290_GLYSO]	0	2
A0A0R4J455	Uricase OS=Glycine max GN=GLYMA_U032500 PE=3 SV=1 - [A0A0R4J455_SOYBN]	0	2
C6T0R5	Uncharacterized protein OS=Glycine max GN=GLYMA_10G150800 PE=2 SV=1 - [C6T0R5_SOYBN]	0	2

A0A0R0KRW0	Uncharacterized protein OS=Glycine max GN=GLYMA_03G163500 PE=4 SV=1 - [A0A0R0KRW0_SOYBN]	0	2
O22378	Metallothionein-II protein OS=Glycine max GN=PGMPM19 PE=2 SV=1 - [O22378_SOYBN]	0	3
I1KQW4	Uncharacterized protein OS=Glycine max PE=3 SV=2 - [I1KQW4_SOYBN]	0	3
I1JPZ4	Uncharacterized protein OS=Glycine max GN=GLYMA_03G193100 PE=3 SV=1 - [I1JPZ4_SOYBN]	0	3
A0A0R4J4U4	Uncharacterized protein OS=Glycine max GN=GLYMA_13G035200 PE=3 SV=1 - [A0A0R4J4U4_SOYBN]	0	3

Table S8. The list of DEPs in the sample 114/118 (MON87705/MON87708).

ID	Description	Sig. (Diff.114/118) times	
		up	down
A0A0R0IIZ3	Uncharacterized protein OS=Glycine max GN=GLYMA_09G178100 PE=3 SV=1 - [A0A0R0IIZ3_SOYBN]	3	0
I1N583	Uncharacterized protein OS=Glycine max GN=GLYMA_18G292300 PE=4 SV=1 - [I1N583_SOYBN]	3	0
C6TAQ9	Uncharacterized protein OS=Glycine max GN=GLYMA_15G156700 PE=2 SV=1 - [C6TAQ9_SOYBN]	3	0
I1LM67	Uncharacterized protein OS=Glycine max GN=GLYMA_13G153000 PE=4 SV=1 - [I1LM67_SOYBN]	3	0
A0A0B2QAS8	Glycine-rich RNA-binding protein 2, mitochondrial OS=Glycine soja GN=glysoja_006768 PE=4 SV=1 - [A0A0B2QAS8_GLYSO]	3	0
A0A0B2RG07	Calnexin like OS=Glycine soja GN=glysoja_012889 PE=3 SV=1 - [A0A0B2RG07_GLYSO]	3	0
P62163	Calmodulin-2 OS=Glycine max GN=CAM-2 PE=1 SV=2 - [CALM2_SOYBN]	3	0
Q71LY8	3-phosphoshikimate 1-carboxyvinyltransferase OS=Glycine max PE=3 SV=1 - [Q71LY8_SOYBN]	3	0
K7MZ42	Uncharacterized protein OS=Glycine max GN=GLYMA_19G186900 PE=4 SV=1 - [K7MZ42_SOYBN]	3	0
A0A0B2R4V3	Uncharacterized protein OS=Glycine soja GN=glysoja_035516 PE=4 SV=1 - [A0A0B2R4V3_GLYSO]	2	0
A0A0B2P7Y1	Ran-binding protein 1 like b OS=Glycine soja GN=glysoja_046443 PE=4 SV=1 - [A0A0B2P7Y1_GLYSO]	2	0
I1JR89	Uncharacterized protein OS=Glycine max GN=GLYMA_03G234400 PE=4 SV=1 - [I1JR89_SOYBN]	2	0
A0A0B2SQP4	Clustered mitochondria protein homolog OS=Glycine soja GN=glysoja_031237 PE=3 SV=1 - [A0A0B2SQP4_GLYSO]	2	0
A0A0B2QYK0	U-box domain-containing protein 72 OS=Glycine soja GN=glysoja_028724 PE=4 SV=1 - [A0A0B2QYK0_GLYSO]	2	0

Q852U4	Glycinin A1bB2-784 OS=Glycine max PE=2 SV=1 - [Q852U4_SOYBN]	2	0
A0A0R0J9Y0	Uncharacterized protein OS=Glycine max GN=GLYMA_07G195900 PE=4 SV=1 - [A0A0R0J9Y0_SOYBN]	2	0
A0A0R0EM21	Uncharacterized protein OS=Glycine max GN=GLYMA_20G126600 PE=4 SV=1 - [A0A0R0EM21_SOYBN]	2	0
I1KS58	Uncharacterized protein OS=Glycine max GN=GLYMA_08G108000 PE=4 SV=1 - [I1KS58_SOYBN]	2	0
I1M395	Uncharacterized protein OS=Glycine max GN=GLYMA_13G278000 PE=3 SV=1 - [I1M395_SOYBN]	2	0
A0A0R0KGJ7	Uncharacterized protein OS=Glycine max GN=GLYMA_03G090000 PE=4 SV=1 - [A0A0R0KGJ7_SOYBN]	2	0
I1MG55	Uncharacterized protein OS=Glycine max GN=GLYMA_15G131700 PE=3 SV=1 - [I1MG55_SOYBN]	2	0
I1MJD3	Cysteine synthase OS=Glycine max GN=GLYMA_15G262500 PE=3 SV=1 - [I1MJD3_SOYBN]	2	0
C6TI51	Uncharacterized protein OS=Glycine max GN=GLYMA_08G064100 PE=2 SV=1 - [C6TI51_SOYBN]	2	0
I1JBI5	Uncharacterized protein OS=Glycine max GN=GLYMA_02G014800 PE=3 SV=1 - [I1JBI5_SOYBN]	2	0
I1MBF5	Uncharacterized protein OS=Glycine max GN=GLYMA_14G198000 PE=4 SV=1 - [I1MBF5_SOYBN]	2	0
I1NAI0	Uncharacterized protein OS=Glycine max GN=GLYMA_19G190400 PE=4 SV=2 - [I1NAI0_SOYBN]	2	0
A0A0R0E916	Uncharacterized protein OS=Glycine max GN=GLYMA_20G089400 PE=4 SV=1 - [A0A0R0E916_SOYBN]	2	0
I1JLC8	Protein SLE2 OS=Glycine max GN=SLE2 PE=2 SV=1 - [SLE2_SOYBN]	2	0
A0A0B2Q1T9	Tryptophan synthase beta chain 2 OS=Glycine soja GN=glysoja_046993 PE=3 SV=1 - [A0A0B2Q1T9_GLYSO]	2	0
K7L817	40S ribosomal protein S12 OS=Glycine max GN=GLYMA_08G217700 PE=3 SV=1 - [K7L817_SOYBN]	2	0
K7LHL1	Uncharacterized protein OS=Glycine max PE=4 SV=1 - [K7LHL1_SOYBN]	2	0
A0A0R0IKB3	Uncharacterized protein (Fragment) OS=Glycine max GN=GLYMA_09G2786001 PE=4 SV=1 - [A0A0R0IKB3_SOYBN]	2	0
K7MVA6	Uncharacterized protein OS=Glycine max GN=GLYMA_18G285000 PE=4 SV=1 - [K7MVA6_SOYBN]	2	0
C6TI49	Putative uncharacterized protein OS=Glycine max PE=2 SV=1 -	2	0

	[C6TI49_SOYBN]		
I1JC44	Uncharacterized protein OS=Glycine max GN=GLYMA_02G036300 PE=4 SV=1 - [I1JC44_SOYBN]	2	0
A0A0R0EJU2	Uncharacterized protein OS=Glycine max GN=GLYMA_19G065600 PE=4 SV=1 - [A0A0R0EJU2_SOYBN]	2	0
A0A097PK35	Pollen Ole e 1 allergen and extensin family protein (Fragment) OS=Glycine max PE=2 SV=1 - [A0A097PK35_SOYBN]	2	0
I1L7J2	Uncharacterized protein OS=Glycine max GN=GLYMA_10G007800 PE=4 SV=1 - [I1L7J2_SOYBN]	2	0
A0A0B2Q1U6	Uncharacterized protein OS=Glycine soja GN=glysoja_025011 PE=4 SV=1 - [A0A0B2Q1U6_GLYSO]	2	0
Q9ZS21	Lactoylglutathione lyase OS=Glycine max GN=GLXI PE=1 SV=1 - [LGUL_SOYBN]	0	2
A0A0B2RM77	Cysteine proteinase inhibitor OS=Glycine soja GN=glysoja_009953 PE=3 SV=1 - [A0A0B2RM77_GLYSO]	0	2
Q9S7N8	Seed maturation protein PM21 OS=Glycine max GN=PM21 PE=2 SV=1 - [Q9S7N8_SOYBN]	0	2
C6SWE8	Superoxide dismutase [Cu-Zn] OS=Glycine max PE=2 SV=1 - [C6SWE8_SOYBN]	0	2
C6TK60	Putative uncharacterized protein OS=Glycine max PE=2 SV=1 - [C6TK60_SOYBN]	0	2
C6T9W4	Putative Fe(II)-and 2-oxoglutaratedependent dioxygenase OS=Glycine max GN=F6'H1 PE=2 SV=1 - [C6T9W4_SOYBN]	0	2
C6T0R5	Uncharacterized protein OS=Glycine max GN=GLYMA_10G150800 PE=2 SV=1 - [C6T0R5_SOYBN]	0	2
O22378	Metallothionein-II protein OS=Glycine max GN=PGMPM19 PE=2 SV=1 - [O22378_SOYBN]	0	3
I1KQW4	Uncharacterized protein OS=Glycine max PE=3 SV=2 - [I1KQW4_SOYBN]	0	3
I1KMS6	Uncharacterized protein OS=Glycine max GN=GLYMA_07G241400 PE=4 SV=1 - [I1KMS6_SOYBN]	0	3

Table S9. The list of DEPs in the sample 115/116 (MON87701×MON89788/FG72).

ID	Description	Sig. (Diff.115/116) times	
		up	down
A0A0B2PMR9	Em-like protein GEA6 OS=Glycine soja GN=glysoja_045231 PE=4 SV=1 - [A0A0B2PMR9_GLYSO]	3	0
I1L8Q0	Lambda class glutathione S-transferase OS=Glycine max GN=GSTL3 PE=2 SV=1 - [I1L8Q0_SOYBN]	3	0
F7J077	Beta-conglycinin beta subunit OS=Glycine max GN=CG-beta-2 PE=4 SV=1 - [F7J077_SOYBN]	3	0
A0A0B2S880	5-methyltetrahydropteroyltriglutamate--homocysteine	3	0

	methyltransferase OS=Glycine soja GN=glysoja_048610 PE=3 SV=1 - [A0A0B2S880_GLYSO]		
A0A0B2QVE6	Beta-glucosidase 44 OS=Glycine soja GN=glysoja_033611 PE=3 SV=1 - [A0A0B2QVE6_GLYSO]	3	0
A0A0R0K7M8	Uncharacterized protein OS=Glycine max GN=GLYMA_04G122900 PE=4 SV=1 - [A0A0R0K7M8_SOYBN]	3	0
A0A0B2SSB2	26.5 kDa heat shock protein, mitochondrial OS=Glycine soja GN=glysoja_029181 PE=3 SV=1 - [A0A0B2SSB2_GLYSO]	3	0
K7K4G2	Uncharacterized protein OS=Glycine max GN=GLYMA_01G177000 PE=4 SV=1 - [K7K4G2_SOYBN]	3	0
P45458	Malate synthase, glyoxysomal (Fragment) OS=Glycine max PE=2 SV=1 - [MASY_SOYBN]	3	0
A0A0B2SQB3	LysM domain-containing GPI-anchored protein 1 OS=Glycine soja GN=glysoja_018395 PE=4 SV=1 - [A0A0B2SQB3_GLYSO]	3	0
I1KDB2	Uncharacterized protein OS=Glycine max GN=GLYMA_06G216700 PE=4 SV=1 - [I1KDB2_SOYBN]	3	0
A0A0R4J681	Uncharacterized protein OS=Glycine max GN=GLYMA_20G233000 PE=4 SV=1 - [A0A0R4J681_SOYBN]	3	0
Q04672	Sucrose-binding protein OS=Glycine max GN=SBP PE=1 SV=1 - [SBP_SOYBN]	3	0
A0A0R0IKB3	Uncharacterized protein (Fragment) OS=Glycine max GN=GLYMA_09G2786001 PE=4 SV=1 - [A0A0R0IKB3_SOYBN]	3	0
A1KR24	Dehydrin OS=Glycine max GN=LEA-2-D11 PE=3 SV=1 - [A1KR24_SOYBN]	3	0
I1L5Z8	Uncharacterized protein OS=Glycine max GN=GLYMA_09G240300 PE=4 SV=1 - [I1L5Z8_SOYBN]	3	0
Q71LY8	3-phosphoshikimate 1-carboxyvinyltransferase OS=Glycine max PE=3 SV=1 - [Q71LY8_SOYBN]	3	0
K7MZ42	Uncharacterized protein OS=Glycine max GN=GLYMA_19G186900 PE=4 SV=1 - [K7MZ42_SOYBN]	3	0
C6T6C3	Putative uncharacterized protein OS=Glycine max PE=2 SV=1 - [C6T6C3_SOYBN]	2	0
A0A0B2R4V3	Uncharacterized protein OS=Glycine soja GN=glysoja_035516 PE=4 SV=1 - [A0A0B2R4V3_GLYSO]	2	0
I1MEH7	Uncharacterized protein OS=Glycine max GN=GLYMA_15G073100 PE=4 SV=1 - [I1MEH7_SOYBN]	2	0
A0A0B2SIS3	Peroxidase OS=Glycine soja GN=glysoja_032062 PE=3 SV=1 - [A0A0B2SIS3_GLYSO]	2	0
A0A0B2Q5D1	Extended synaptotagmin-3 OS=Glycine soja GN=glysoja_011660 PE=4 SV=1 - [A0A0B2Q5D1_GLYSO]	2	0
I1M138	Uncharacterized protein OS=Glycine max GN=GLYMA_13G208000 PE=3 SV=1 - [I1M138_SOYBN]	2	0

C6TMV9	Uncharacterized protein OS=Glycine max GN=GLYMA_03G212700 PE=2 SV=1 - [C6TMV9_SOYBN]	2	0
A0A0B2NX08	T-complex protein 1 subunit gamma OS=Glycine soja GN=glysoja_023929 PE=3 SV=1 - [A0A0B2NX08_GLYSO]	2	0
Q9FQE8	Glutathione S-transferase OS=Glycine max GN=GSTU41 PE=1 SV=1 - [Q9FQE8_SOYBN]	2	0
I1JXM1	HVA22-like protein OS=Glycine max GN=GLYMA_04G197600 PE=3 SV=1 - [I1JXM1_SOYBN]	2	0
A0A0R0HKE3	Uncharacterized protein OS=Glycine max GN=GLYMA_11G214800 PE=4 SV=1 - [A0A0R0HKE3_SOYBN]	2	0
A0A0R0EM21	Uncharacterized protein OS=Glycine max GN=GLYMA_20G126600 PE=4 SV=1 - [A0A0R0EM21_SOYBN]	2	0
I1JAQ7	Uncharacterized protein OS=Glycine max GN=GLYMA_01G235600 PE=4 SV=2 - [I1JAQ7_SOYBN]	2	0
K7N254	Uncharacterized protein OS=Glycine max GN=GLYMA_20G075100 PE=4 SV=1 - [K7N254_SOYBN]	2	0
K7L1Z5	Uncharacterized protein OS=Glycine max GN=GLYMA_07G157200 PE=4 SV=1 - [K7L1Z5_SOYBN]	2	0
I1L2M6	Uncharacterized protein OS=Glycine max GN=GLYMA_09G112100 PE=4 SV=1 - [I1L2M6_SOYBN]	2	0
A0A0B2NW50	Lipoxygenase OS=Glycine soja GN=glysoja_009577 PE=3 SV=1 - [A0A0B2NW50_GLYSO]	2	0
A0A0B2PSI9	Putative calcium-binding protein CML27 OS=Glycine soja GN=glysoja_040702 PE=4 SV=1 - [A0A0B2PSI9_GLYSO]	2	0
I1ND90	Carboxypeptidase OS=Glycine max GN=GLYMA_20G016800 PE=3 SV=2 - [I1ND90_SOYBN]	2	0
A0A0B2QAS8	Glycine-rich RNA-binding protein 2, mitochondrial OS=Glycine soja GN=glysoja_006768 PE=4 SV=1 - [A0A0B2QAS8_GLYSO]	2	0
C6T7Q4	Putative uncharacterized protein OS=Glycine max PE=2 SV=1 - [C6T7Q4_SOYBN]	2	0
A0A0R0JFI6	Endoglucanase OS=Glycine max GN=GLYMA_07G270600 PE=3 SV=1 - [A0A0R0JFI6_SOYBN]	2	0
I1JDI7	Uncharacterized protein OS=Glycine max GN=GLYMA_02G086200 PE=4 SV=1 - [I1JDI7_SOYBN]	2	0
A0A0B2S7G7	Cullin-associated NEDD8-dissociated protein 1 OS=Glycine soja GN=glysoja_038397 PE=4 SV=1 - [A0A0B2S7G7_GLYSO]	2	0
C6T9W4	Putative Fe(II)-and 2-oxoglutaratedependent dioxygenase OS=Glycine max GN=F6'H1 PE=2 SV=1 - [C6T9W4_SOYBN]	2	0
I1MFH9	Lactoylglutathione lyase OS=Glycine max GN=GLYMA_15G108400 PE=3 SV=1 - [I1MFH9_SOYBN]	2	0
C6ZJY8	Serine hydroxymethyltransferase OS=Glycine max GN=GLYMA_08G274400 PE=2 SV=1 - [C6ZJY8_SOYBN]	2	0

A0A0B2R5T6	Malic enzyme (Fragment) OS=Glycine soja GN=glysoja_034656 PE=3 SV=1 - [A0A0B2R5T6_GLYSO]	2	0
I1KQE3	Uncharacterized protein OS=Glycine max GN=GLYMA_08G048200 PE=3 SV=1 - [I1KQE3_SOYBN]	2	0
I1KVR2	Uncharacterized protein OS=Glycine max GN=GLYMA_08G222100 PE=3 SV=1 - [I1KVR2_SOYBN]	2	0
A0A0B2Q0G3	Outer plastidial membrane protein porin OS=Glycine soja GN=glysoja_016419 PE=4 SV=1 - [A0A0B2Q0G3_GLYSO]	2	0
I1L922	Uncharacterized protein OS=Glycine max GN=GLYMA_10G060200 PE=3 SV=2 - [I1L922_SOYBN]	2	0
Q70EM0	Dehydrin OS=Glycine max GN=lea-D-11 PE=3 SV=1 - [Q70EM0_SOYBN]	2	0
A0A0B2S2I4	Nicalin-1 (Fragment) OS=Glycine soja GN=glysoja_008163 PE=4 SV=1 - [A0A0B2S2I4_GLYSO]	2	0
C6SZC5	Uncharacterized protein OS=Glycine max GN=GLYMA_18G246300 PE=2 SV=1 - [C6SZC5_SOYBN]	2	0
I1KHR5	Uncharacterized protein OS=Glycine max GN=GLYMA_07G051300 PE=4 SV=1 - [I1KHR5_SOYBN]	2	0
C6TCA2	Putative uncharacterized protein OS=Glycine max PE=2 SV=1 - [C6TCA2_SOYBN]	2	0
Q6PV94	Thioredoxin OS=Glycine max PE=2 SV=1 - [Q6PV94_SOYBN]	2	0
A0A0B2RCV2	Puromycin-sensitive aminopeptidase OS=Glycine soja GN=glysoja_016324 PE=4 SV=1 - [A0A0B2RCV2_GLYSO]	0	2
A0A0B2RM77	Cysteine proteinase inhibitor OS=Glycine soja GN=glysoja_009953 PE=3 SV=1 - [A0A0B2RM77_GLYSO]	0	2
I1KZJ1	Uncharacterized protein OS=Glycine max GN=GLYMA_08G362900 PE=3 SV=1 - [I1KZJ1_SOYBN]	0	2
A0A0B2Q9B8	Oleosin OS=Glycine soja GN=glysoja_025116 PE=3 SV=1 - [A0A0B2Q9B8_GLYSO]	0	2
I1MQ52	Uncharacterized protein OS=Glycine max GN=GLYMA_16G200900 PE=4 SV=1 - [I1MQ52_SOYBN]	0	2
K7KU09	ATP-dependent 6-phosphofructokinase OS=Glycine max GN=PFK PE=3 SV=1 - [K7KU09_SOYBN]	0	2
I1MKY2	Uncharacterized protein OS=Glycine max GN=GLYMA_16G037800 PE=4 SV=1 - [I1MKY2_SOYBN]	0	2
I1NJ59	DHAR class glutathione S-transferase OS=Glycine max GN=DHAR4 PE=2 SV=1 - [I1NJ59_SOYBN]	0	2
I1J7U9	3-phosphoshikimate 1-carboxyvinyltransferase OS=Glycine max GN=GLYMA_01G139600 PE=3 SV=1 - [I1J7U9_SOYBN]	0	2
A0A0B2RHP8	Membrane steroid-binding protein 2 OS=Glycine soja GN=glysoja_029638 PE=3 SV=1 - [A0A0B2RHP8_GLYSO]	0	2
C6SVT0	Uncharacterized protein OS=Glycine max GN=GLYMA_10G147700 PE=2 SV=1 - [C6SVT0_SOYBN]	0	2

A0A0B2QLA9	Peroxiredoxin-2B OS=Glycine soja GN=glysoja_018980 PE=4 SV=1 - [A0A0B2QLA9_GLYSO]	0	2
K7L8B5	Uncharacterized protein OS=Glycine max GN=GLYMA_08G232400 PE=4 SV=1 - [K7L8B5_SOYBN]	0	2
A0A0B2QKF8	Glucose and ribitol dehydrogenase OS=Glycine soja GN=glysoja_023775 PE=4 SV=1 - [A0A0B2QKF8_GLYSO]	0	2
P25273	Kunitz-type trypsin inhibitor KTI2 OS=Glycine max GN=KTI2 PE=2 SV=1 - [KTI2_SOYBN]	0	2
A0A0B2Q4Z1	Elongation factor 1-alpha OS=Glycine soja GN=glysoja_032479 PE=3 SV=1 - [A0A0B2Q4Z1_GLYSO]	0	2
A0A0B2SQI5	Uncharacterized protein OS=Glycine soja GN=glysoja_015000 PE=4 SV=1 - [A0A0B2SQI5_GLYSO]	0	2
A0A0R0F0E0	Uncharacterized protein OS=Glycine max GN=GLYMA_18G153900 PE=4 SV=1 - [A0A0R0F0E0_SOYBN]	0	2
O22378	Metallothionein-II protein OS=Glycine max GN=PGMPM19 PE=2 SV=1 - [O22378_SOYBN]	0	3
I1MYI8	Glyceraldehyde-3-phosphate dehydrogenase OS=Glycine max GN=GLYMA_18G009700 PE=3 SV=1 - [I1MYI8_SOYBN]	0	3
I1KYW3	Uncharacterized protein OS=Glycine max GN=GLYMA_08G341000 PE=4 SV=1 - [I1KYW3_SOYBN]	0	3
A0A0R4J4U4	Uncharacterized protein OS=Glycine max GN=GLYMA_13G035200 PE=3 SV=1 - [A0A0R4J4U4_SOYBN]	0	3
C6SYM8	Uncharacterized protein OS=Glycine max GN=GLYMA_03G238700 PE=2 SV=1 - [C6SYM8_SOYBN]	0	3
I1MIA8	Uncharacterized protein OS=Glycine max GN=GLYMA_15G217700 PE=4 SV=1 - [I1MIA8_SOYBN]	0	3
C6TD56	Glyceraldehyde-3-phosphate dehydrogenase OS=Glycine max GN=GLYMA_11G247600 PE=2 SV=1 - [C6TD56_SOYBN]	0	3
O22121	Beta subunit of beta conglycinin (Fragment) OS=Glycine max PE=2 SV=2 - [O22121_SOYBN]	0	3
K7MLV5	Uncharacterized protein OS=Glycine max PE=3 SV=1 - [K7MLV5_SOYBN]	0	3
I1N6Q6	Ribulose bisphosphate carboxylase small chain OS=Glycine max GN=GLYMA_19G046600 PE=3 SV=1 - [I1N6Q6_SOYBN]	0	3
I1MAE6	Uncharacterized protein OS=Glycine max GN=GLYMA_14G121200 PE=3 SV=1 - [I1MAE6_SOYBN]	0	3
C6T3U6	Ribosomal protein L19 OS=Glycine max GN=GLYMA_19G208200 PE=2 SV=1 - [C6T3U6_SOYBN]	0	3
A0A0R0JL19	Uncharacterized protein OS=Glycine max GN=GLYMA_06G122600 PE=3 SV=1 - [A0A0R0JL19_SOYBN]	0	3
I1JGP8	Uncharacterized protein OS=Glycine max	0	3

	GN=GLYMA_02G204000 PE=3 SV=1 - [I1JGP8_SOYBN]		
A0A0R4J455	Uricase OS=Glycine max GN=GLYMA_U032500 PE=3 SV=1 - [A0A0R4J455_SOYBN]	0	3

Table S10. The list of DEPs in the sample 114/116 (MON87705/FG72).

ID	Description	Sig. (Diff.114/116) times	
		up	down
I1M395	Uncharacterized protein OS=Glycine max GN=GLYMA_13G278000 PE=3 SV=1 - [I1M395_SOYBN]	3	0
A0A0B2QVE6	Beta-glucosidase 44 OS=Glycine soja GN=glysoja_033611 PE=3 SV=1 - [A0A0B2QVE6_GLYSO]	3	0
I1LM67	Uncharacterized protein OS=Glycine max GN=GLYMA_13G153000 PE=4 SV=1 - [I1LM67_SOYBN]	3	0
A0A0R0K7M8	Uncharacterized protein OS=Glycine max GN=GLYMA_04G122900 PE=4 SV=1 - [A0A0R0K7M8_SOYBN]	3	0
A0A0B2SSB2	26.5 kDa heat shock protein, mitochondrial OS=Glycine soja GN=glysoja_029181 PE=3 SV=1 - [A0A0B2SSB2_GLYSO]	3	0
K7K4G2	Uncharacterized protein OS=Glycine max GN=GLYMA_01G177000 PE=4 SV=1 - [K7K4G2_SOYBN]	3	0
A0A0B2QAS8	Glycine-rich RNA-binding protein 2, mitochondrial OS=Glycine soja GN=glysoja_006768 PE=4 SV=1 - [A0A0B2QAS8_GLYSO]	3	0
A0A0R0E916	Uncharacterized protein OS=Glycine max GN=GLYMA_20G089400 PE=4 SV=1 - [A0A0R0E916_SOYBN]	3	0
I1KQE3	Uncharacterized protein OS=Glycine max GN=GLYMA_08G048200 PE=3 SV=1 - [I1KQE3_SOYBN]	3	0
A0A0R0IKB3	Uncharacterized protein (Fragment) OS=Glycine max GN=GLYMA_09G2786001 PE=4 SV=1 - [A0A0R0IKB3_SOYBN]	3	0
P62163	Calmodulin-2 OS=Glycine max GN=CAM-2 PE=1 SV=2 - [CALM2_SOYBN]	3	0
A0A0B2R887	Peroxygenase OS=Glycine soja GN=glysoja_034064 PE=4 SV=1 - [A0A0B2R887_GLYSO]	3	0
Q71LY8	3-phosphoshikimate 1-carboxyvinyltransferase OS=Glycine max PE=3 SV=1 - [Q71LY8_SOYBN]	3	0
K7MZ42	Uncharacterized protein OS=Glycine max GN=GLYMA_19G186900 PE=4 SV=1 - [K7MZ42_SOYBN]	3	0
A0A0R0HH51	Uncharacterized protein OS=Glycine max GN=GLYMA_12G112900 PE=4 SV=1 - [A0A0R0HH51_SOYBN]	2	0
C6TFT6	4-hydroxy-4-methyl-2-oxoglutarate aldolase OS=Glycine max PE=2 SV=1 - [C6TFT6_SOYBN]	2	0
I1J7M1	Non-specific lipid-transfer protein OS=Glycine max	2	0

	GN=GLYMA_01G130200 PE=3 SV=2 - [I1J7M1_SOYBN]		
O22120	Alpha subunit of beta conglycinin (Fragment) OS=Glycine max PE=2 SV=2 - [O22120_SOYBN]	2	0
I1N583	Uncharacterized protein OS=Glycine max GN=GLYMA_18G292300 PE=4 SV=1 - [I1N583_SOYBN]	2	0
I1M138	Uncharacterized protein OS=Glycine max GN=GLYMA_13G208000 PE=3 SV=1 - [I1M138_SOYBN]	2	0
A0A0B2QYK0	U-box domain-containing protein 72 OS=Glycine soja GN=glysoja_028724 PE=4 SV=1 - [A0A0B2QYK0_GLYSO]	2	0
Q852U4	Glycinin A1bB2-784 OS=Glycine max PE=2 SV=1 - [Q852U4_SOYBN]	2	0
A0A0R0J9Y0	Uncharacterized protein OS=Glycine max GN=GLYMA_07G195900 PE=4 SV=1 - [A0A0R0J9Y0_SOYBN]	2	0
A0A0R0KGJ7	Uncharacterized protein OS=Glycine max GN=GLYMA_03G090000 PE=4 SV=1 - [A0A0R0KGJ7_SOYBN]	2	0
A0A0B2R220	60S ribosomal protein L11 OS=Glycine soja GN=glysoja_039616 PE=3 SV=1 - [A0A0B2R220_GLYSO]	2	0
I1LNM2	NADH dehydrogenase subunit 9 OS=Glycine max GN=nad9 PE=3 SV=1 - [I1LNM2_SOYBN]	2	0
I1JBI5	Uncharacterized protein OS=Glycine max GN=GLYMA_02G014800 PE=3 SV=1 - [I1JBI5_SOYBN]	2	0
P45458	Malate synthase, glyoxysomal (Fragment) OS=Glycine max PE=2 SV=1 - [MASY_SOYBN]	2	0
A0A0B2SQB3	LysM domain-containing GPI-anchored protein 1 OS=Glycine soja GN=glysoja_018395 PE=4 SV=1 - [A0A0B2SQB3_GLYSO]	2	0
A0A0B2NSC6	40S ribosomal protein S5 OS=Glycine soja GN=glysoja_034227 PE=3 SV=1 - [A0A0B2NSC6_GLYSO]	2	0
A0A0B2Q1T9	Tryptophan synthase beta chain 2 OS=Glycine soja GN=glysoja_046993 PE=3 SV=1 - [A0A0B2Q1T9_GLYSO]	2	0
A0A0B2S7G7	Cullin-associated NEDD8-dissociated protein 1 OS=Glycine soja GN=glysoja_038397 PE=4 SV=1 - [A0A0B2S7G7_GLYSO]	2	0
A0A0B2R5T6	Malic enzyme (Fragment) OS=Glycine soja GN=glysoja_034656 PE=3 SV=1 - [A0A0B2R5T6_GLYSO]	2	0
A0A0B2R0Z0	1-Cys peroxiredoxin OS=Glycine soja GN=glysoja_018783 PE=4 SV=1 - [A0A0B2R0Z0_GLYSO]	2	0
K7LHL1	Uncharacterized protein OS=Glycine max PE=4 SV=1 - [K7LHL1_SOYBN]	2	0
C6TI49	Putative uncharacterized protein OS=Glycine max PE=2 SV=1 - [C6TI49_SOYBN]	2	0
I1JC44	Uncharacterized protein OS=Glycine max GN=GLYMA_02G036300 PE=4 SV=1 - [I1JC44_SOYBN]	2	0
A1KR24	Dehydrin OS=Glycine max GN=LEA-2-D11 PE=3 SV=1 -	2	0

	[A1KR24_SOYBN]		
A0A0B2QY19	Uncharacterized protein OS=Glycine soja GN=glysoja_019805 PE=4 SV=1 - [A0A0B2QY19_GLYSO]	2	0
I1MK74	Uncharacterized protein OS=Glycine max GN=GLYMA_16G013900 PE=4 SV=1 - [I1MK74_SOYBN]	2	0
C6T529	Ribulose bisphosphate carboxylase small chain OS=Glycine max PE=2 SV=1 - [C6T529_SOYBN]	2	0
I1L7J2	Uncharacterized protein OS=Glycine max GN=GLYMA_10G007800 PE=4 SV=1 - [I1L7J2_SOYBN]	2	0
A0A0R0EBC1	Uncharacterized protein OS=Glycine max GN=GLYMA_20G148100 PE=4 SV=1 - [A0A0R0EBC1_SOYBN]	2	0
A0A0R0ENW6	Uncharacterized protein OS=Glycine max GN=GLYMA_20G210100 PE=3 SV=1 - [A0A0R0ENW6_SOYBN]	0	2
C6T078	Uncharacterized protein OS=Glycine max GN=GLYMA_13G133900 PE=2 SV=1 - [C6T078_SOYBN]	0	2
A0A0B2PKZ5	Vacuolar-processing enzyme OS=Glycine soja GN=glysoja_017086 PE=4 SV=1 - [A0A0B2PKZ5_GLYSO]	0	2
C6SXH8	Putative uncharacterized protein OS=Glycine max PE=2 SV=1 - [C6SXH8_SOYBN]	0	2
A0A0B2Q9B8	Oleosin OS=Glycine soja GN=glysoja_025116 PE=3 SV=1 - [A0A0B2Q9B8_GLYSO]	0	2
A0A0B2RVW2	Thioredoxin M-type, chloroplastic OS=Glycine soja GN=glysoja_037309 PE=4 SV=1 - [A0A0B2RVW2_GLYSO]	0	2
I1LPX6	Fructose-bisphosphate aldolase OS=Glycine max GN=GLYMA_12G037400 PE=3 SV=1 - [I1LPX6_SOYBN]	0	2
A0A0B2NW50	Lipoxygenase OS=Glycine soja GN=glysoja_009577 PE=3 SV=1 - [A0A0B2NW50_GLYSO]	0	2
A0A0R4J4X6	Uncharacterized protein OS=Glycine max GN=GLYMA_13G176300 PE=3 SV=1 - [A0A0R4J4X6_SOYBN]	0	2
K7MNF4	Uncharacterized protein OS=Glycine max GN=GLYMA_17G227500 PE=3 SV=1 - [K7MNF4_SOYBN]	0	2
I1NJ59	DHAR class glutathione S-transferase OS=Glycine max GN=DHAR4 PE=2 SV=1 - [I1NJ59_SOYBN]	0	2
I1J7U9	3-phosphoshikimate 1-carboxyvinyltransferase OS=Glycine max GN=GLYMA_01G139600 PE=3 SV=1 - [I1J7U9_SOYBN]	0	2
I1KV72	Uncharacterized protein OS=Glycine max GN=GLYMA_08G205200 PE=4 SV=1 - [I1KV72_SOYBN]	0	2
I1JGP8	Uncharacterized protein OS=Glycine max GN=GLYMA_02G204000 PE=3 SV=1 - [I1JGP8_SOYBN]	0	2
A0A0R0F0E0	Uncharacterized protein OS=Glycine max GN=GLYMA_18G153900 PE=4 SV=1 -	0	2

	[A0A0R0F0E0_SOYBN]		
A0A0R4J455	Uricase OS=Glycine max GN=GLYMA_U032500 PE=3 SV=1 - [A0A0R4J455_SOYBN]	0	2
C6SX26	Putative uncharacterized protein OS=Glycine max PE=2 SV=1 - [C6SX26_SOYBN]	0	3
I1MEH3	Uncharacterized protein OS=Glycine max GN=GLYMA_15G072900 PE=4 SV=1 - [I1MEH3_SOYBN]	0	3
O22378	Metallothionein-II protein OS=Glycine max GN=PGMPM19 PE=2 SV=1 - [O22378_SOYBN]	0	3
C6SZX7	Glutathione peroxidase OS=Glycine max GN=GLYMA_01G219400 PE=2 SV=1 - [C6SZX7_SOYBN]	0	3
I1MYI8	Glyceraldehyde-3-phosphate dehydrogenase OS=Glycine max GN=GLYMA_18G009700 PE=3 SV=1 - [I1MYI8_SOYBN]	0	3
A0A0B2RM77	Cysteine proteinase inhibitor OS=Glycine soja GN=glysoja_009953 PE=3 SV=1 - [A0A0B2RM77_GLYSO]	0	3
I1KYW3	Uncharacterized protein OS=Glycine max GN=GLYMA_08G341000 PE=4 SV=1 - [I1KYW3_SOYBN]	0	3
C6SWE8	Superoxide dismutase [Cu-Zn] OS=Glycine max PE=2 SV=1 - [C6SWE8_SOYBN]	0	3
K7KU09	ATP-dependent 6-phosphofructokinase OS=Glycine max GN=PFK PE=3 SV=1 - [K7KU09_SOYBN]	0	3
I1MIA8	Uncharacterized protein OS=Glycine max GN=GLYMA_15G217700 PE=4 SV=1 - [I1MIA8_SOYBN]	0	3
C6TD56	Glyceraldehyde-3-phosphate dehydrogenase OS=Glycine max GN=GLYMA_11G247600 PE=2 SV=1 - [C6TD56_SOYBN]	0	3
I1MXZ6	Uncharacterized protein OS=Glycine max GN=GLYMA_17G254200 PE=4 SV=1 - [I1MXZ6_SOYBN]	0	3
O22121	Beta subunit of beta conglycinin (Fragment) OS=Glycine max PE=2 SV=2 - [O22121_SOYBN]	0	3
K7MLV5	Uncharacterized protein OS=Glycine max PE=3 SV=1 - [K7MLV5_SOYBN]	0	3
I1MAE6	Uncharacterized protein OS=Glycine max GN=GLYMA_14G121200 PE=3 SV=1 - [I1MAE6_SOYBN]	0	3
A0A0B2QUL3	Tropinone reductase like OS=Glycine soja GN=glysoja_010147 PE=4 SV=1 - [A0A0B2QUL3_GLYSO]	0	3
C6SVT0	Uncharacterized protein OS=Glycine max GN=GLYMA_10G147700 PE=2 SV=1 - [C6SVT0_SOYBN]	0	3
A0A0B2QLA9	Peroxiredoxin-2B OS=Glycine soja GN=glysoja_018980 PE=4 SV=1 - [A0A0B2QLA9_GLYSO]	0	3
A0A0B2SQI5	Uncharacterized protein OS=Glycine soja GN=glysoja_015000 PE=4 SV=1 - [A0A0B2SQI5_GLYSO]	0	3
K7N4A7	Uncharacterized protein OS=Glycine max PE=3 SV=1 - [K7N4A7_SOYBN]	0	3

I1KIE4	Uncharacterized protein OS=Glycine max GN=GLYMA_07G073800 PE=3 SV=1 - [I1KIE4_SOYBN]	0	3

Table S11. The list of DEPs in the sample 118/116 (MON87708/FG72).

ID	Description	Sig. (Diff.118/116) times	
		up	down
I1KQW4	Uncharacterized protein OS=Glycine max PE=3 SV=2 - [I1KQW4_SOYBN]	3	0
Q9S7N8	Seed maturation protein PM21 OS=Glycine max GN=PM21 PE=2 SV=1 - [Q9S7N8_SOYBN]	3	0
I1JVL6	Uncharacterized protein OS=Glycine max GN=GLYMA_04G111500 PE=3 SV=1 - [I1JVL6_SOYBN]	3	0
A0A0B2QVE6	Beta-glucosidase 44 OS=Glycine soja GN=glysoja_033611 PE=3 SV=1 - [A0A0B2QVE6_GLYSO]	3	0
A0A0B2PSI9	Putative calcium-binding protein CML27 OS=Glycine soja GN=glysoja_040702 PE=4 SV=1 - [A0A0B2PSI9_GLYSO]	3	0
A0A0B2SSB2	26.5 kDa heat shock protein, mitochondrial OS=Glycine soja GN=glysoja_029181 PE=3 SV=1 - [A0A0B2SSB2_GLYSO]	3	0
K7K4G2	Uncharacterized protein OS=Glycine max GN=GLYMA_01G177000 PE=4 SV=1 - [K7K4G2_SOYBN]	3	0
A0A0B2SQB3	LysM domain-containing GPI-anchored protein 1 OS=Glycine soja GN=glysoja_018395 PE=4 SV=1 - [A0A0B2SQB3_GLYSO]	3	0
A0A0B2Q5P0	Uncharacterized protein OS=Glycine soja GN=glysoja_011760 PE=4 SV=1 - [A0A0B2Q5P0_GLYSO]	3	0
I1KQE3	Uncharacterized protein OS=Glycine max GN=GLYMA_08G048200 PE=3 SV=1 - [I1KQE3_SOYBN]	3	0
Q02243	Wound-induced protein (Fragment) OS=Glycine max GN=WIN PE=2 SV=1 - [WIN_SOYBN]	3	0
A1KR24	Dehydrin OS=Glycine max GN=LEA-2-D11 PE=3 SV=1 - [A1KR24_SOYBN]	3	0
C6TMA8	Uncharacterized protein OS=Glycine max GN=GLYMA_07G226600 PE=2 SV=1 - [C6TMA8_SOYBN]	3	0
I1JXM1	HVA22-like protein OS=Glycine max GN=GLYMA_04G197600 PE=3 SV=1 - [I1JXM1_SOYBN]	2	0
A0A0B2S880	5-methyltetrahydropteroyltriglutamate--homocysteine methyltransferase OS=Glycine soja GN=glysoja_048610 PE=3 SV=1 - [A0A0B2S880_GLYSO]	2	0
C6SXP1	Putative uncharacterized protein OS=Glycine max PE=2 SV=1 - [C6SXP1_SOYBN]	2	0
A0A0B2R220	60S ribosomal protein L11 OS=Glycine soja GN=glysoja_039616 PE=3 SV=1 - [A0A0B2R220_GLYSO]	2	0
A0A0R0K7M8	Uncharacterized protein OS=Glycine max GN=GLYMA_04G122900 PE=4 SV=1 - [A0A0R0K7M8_SOYBN]	2	0

A0A0R0EDR0	Uncharacterized protein OS=Glycine max GN=GLYMA_20G196600 PE=4 SV=1 - [A0A0R0EDR0_SOYBN]	2	0
I1MU68	Uncharacterized protein OS=Glycine max GN=GLYMA_17G112500 PE=4 SV=2 - [I1MU68_SOYBN]	2	0
I1JE09	Uncharacterized protein OS=Glycine max GN=GLYMA_02G103600 PE=3 SV=1 - [I1JE09_SOYBN]	2	0
I1ND90	Carboxypeptidase OS=Glycine max GN=GLYMA_20G016800 PE=3 SV=2 - [I1ND90_SOYBN]	2	0
P45458	Malate synthase, glyoxysomal (Fragment) OS=Glycine max PE=2 SV=1 - [MASY_SOYBN]	2	0
A0A0B2P1M7	U-box domain-containing protein 35 (Fragment) OS=Glycine soja GN=glysoja_009585 PE=4 SV=1 - [A0A0B2P1M7_GLYSO]	2	0
A0A0R0GP29	Uncharacterized protein OS=Glycine max GN=GLYMA_14G170900 PE=4 SV=1 - [A0A0R0GP29_SOYBN]	2	0
A0A0B2S8C8	Uncharacterized protein OS=Glycine soja GN=glysoja_007471 PE=4 SV=1 - [A0A0B2S8C8_GLYSO]	2	0
P25973	Cystatin (Fragment) OS=Glycine max PE=1 SV=1 - [CYT_SOYBN]	2	0
A0A0B2NSC6	40S ribosomal protein S5 OS=Glycine soja GN=glysoja_034227 PE=3 SV=1 - [A0A0B2NSC6_GLYSO]	2	0
A0A0B2S7G7	Cullin-associated NEDD8-dissociated protein 1 OS=Glycine soja GN=glysoja_038397 PE=4 SV=1 - [A0A0B2S7G7_GLYSO]	2	0
A0A0B2R054	Putative lipid-transfer protein DIR1 OS=Glycine soja GN=glysoja_032913 PE=4 SV=1 - [A0A0B2R054_GLYSO]	2	0
I1MFH9	Lactoylglutathione lyase OS=Glycine max GN=GLYMA_15G108400 PE=3 SV=1 - [I1MFH9_SOYBN]	2	0
A0A0B2R0Z0	1-Cys peroxiredoxin OS=Glycine soja GN=glysoja_018783 PE=4 SV=1 - [A0A0B2R0Z0_GLYSO]	2	0
A0A0B2PHR4	Anthocyanin 5-aromatic acyltransferase OS=Glycine soja GN=glysoja_031994 PE=4 SV=1 - [A0A0B2PHR4_GLYSO]	2	0
A0A0B2Q0G3	Outer plastidial membrane protein porin OS=Glycine soja GN=glysoja_016419 PE=4 SV=1 - [A0A0B2Q0G3_GLYSO]	2	0
C6SZC5	Uncharacterized protein OS=Glycine max GN=GLYMA_18G246300 PE=2 SV=1 - [C6SZC5_SOYBN]	2	0
A0A0B2R887	Peroxygenase OS=Glycine soja GN=glysoja_034064 PE=4 SV=1 - [A0A0B2R887_GLYSO]	2	0
A0A0B2R290	Putative glutathione S-transferase OS=Glycine soja GN=glysoja_025575 PE=4 SV=1 - [A0A0B2R290_GLYSO]	2	0
C6T0R5	Uncharacterized protein OS=Glycine max GN=GLYMA_10G150800 PE=2 SV=1 - [C6T0R5_SOYBN]	2	0
A0A0R0IKB3	Uncharacterized protein (Fragment) OS=Glycine max GN=GLYMA_09G2786001 PE=4 SV=1 -	1	2

	[A0A0R0IKB3_SOYBN]		
I1JC44	Uncharacterized protein OS=Glycine max GN=GLYMA_02G036300 PE=4 SV=1 - [I1JC44_SOYBN]	1	2
A0A0B2R4V3	Uncharacterized protein OS=Glycine soja GN=glysoja_035516 PE=4 SV=1 - [A0A0B2R4V3_GLYSO]	0	2
A0A0R0IIZ3	Uncharacterized protein OS=Glycine max GN=GLYMA_09G178100 PE=3 SV=1 - [A0A0R0IIZ3_SOYBN]	0	2
A0A0B2PKZ5	Vacuolar-processing enzyme OS=Glycine soja GN=glysoja_017086 PE=4 SV=1 - [A0A0B2PKZ5_GLYSO]	0	2
A0A0B2RCV2	Puromycin-sensitive aminopeptidase OS=Glycine soja GN=glysoja_016324 PE=4 SV=1 - [A0A0B2RCV2_GLYSO]	0	2
Q852U4	Glycinin A1bB2-784 OS=Glycine max PE=2 SV=1 - [Q852U4_SOYBN]	0	2
I1LCV0	Uncharacterized protein OS=Glycine max GN=GLYMA_10G205900 PE=4 SV=1 - [I1LCV0_SOYBN]	0	2
K7MLV5	Uncharacterized protein OS=Glycine max PE=3 SV=1 - [K7MLV5_SOYBN]	0	2
I1J7U9	3-phosphoshikimate 1-carboxyvinyltransferase OS=Glycine max GN=GLYMA_01G139600 PE=3 SV=1 - [I1J7U9_SOYBN]	0	2
K7L817	40S ribosomal protein S12 OS=Glycine max GN=GLYMA_08G217700 PE=3 SV=1 - [K7L817_SOYBN]	0	2
K7N4A7	Uncharacterized protein OS=Glycine max PE=3 SV=1 - [K7N4A7_SOYBN]	0	2
I1KIE4	Uncharacterized protein OS=Glycine max GN=GLYMA_07G073800 PE=3 SV=1 - [I1KIE4_SOYBN]	0	2
A0A0R0F0E0	Uncharacterized protein OS=Glycine max GN=GLYMA_18G153900 PE=4 SV=1 - [A0A0R0F0E0_SOYBN]	0	2
C6SX26	Putative uncharacterized protein OS=Glycine max PE=2 SV=1 - [C6SX26_SOYBN]	0	3
I1MYI8	Glyceraldehyde-3-phosphate dehydrogenase OS=Glycine max GN=GLYMA_18G009700 PE=3 SV=1 - [I1MYI8_SOYBN]	0	3
I1JFX0	Uncharacterized protein OS=Glycine max GN=GLYMA_02G167700 PE=4 SV=1 - [I1JFX0_SOYBN]	0	3
I1KYW3	Uncharacterized protein OS=Glycine max GN=GLYMA_08G341000 PE=4 SV=1 - [I1KYW3_SOYBN]	0	3
K7KU09	ATP-dependent 6-phosphofructokinase OS=Glycine max GN=PFK PE=3 SV=1 - [K7KU09_SOYBN]	0	3
C6TD56	Glyceraldehyde-3-phosphate dehydrogenase OS=Glycine max GN=GLYMA_11G247600 PE=2 SV=1 - [C6TD56_SOYBN]	0	3
O22121	Beta subunit of beta conglycinin (Fragment) OS=Glycine max PE=2 SV=2 - [O22121_SOYBN]	0	3
I1MAE6	Uncharacterized protein OS=Glycine max GN=GLYMA_14G121200 PE=3 SV=1 - [I1MAE6_SOYBN]	0	3

A0A0B2QUL3	Tropinone reductase like OS=Glycine soja GN=glysoja_010147 PE=4 SV=1 - [A0A0B2QUL3_GLYSO]	0	3
A0A0B2SQI5	Uncharacterized protein OS=Glycine soja GN=glysoja_015000 PE=4 SV=1 - [A0A0B2SQI5_GLYSO]	0	3

Table S12. The list of DEPs in the sample 117/119 (A3525/FG72-JACK).

ID	Description	Sig. (Diff.117/119) times	
		up	down
I1N520	Uncharacterized protein OS=Glycine max GN=GLYMA_18G285800 PE=4 SV=1 - [I1N520_SOYBN]	3	0
A0A0B2PMR9	Em-like protein GEA6 OS=Glycine soja GN=glysoja_045231 PE=4 SV=1 - [A0A0B2PMR9_GLYSO]	3	0
I1KQW4	Uncharacterized protein OS=Glycine max PE=3 SV=2 - [I1KQW4_SOYBN]	3	0
I1LVC1	Uncharacterized protein OS=Glycine max GN=GLYMA_12G235800 PE=4 SV=1 - [I1LVC1_SOYBN]	3	0
A0A0R0IIZ3	Uncharacterized protein OS=Glycine max GN=GLYMA_09G178100 PE=3 SV=1 - [A0A0R0IIZ3_SOYBN]	3	0
I1J7M1	Non-specific lipid-transfer protein OS=Glycine max GN=GLYMA_01G130200 PE=3 SV=2 - [I1J7M1_SOYBN]	3	0
A0A0B2S880	5-methyltetrahydropteroyltriglutamate--homocysteine methyltransferase OS=Glycine soja GN=glysoja_048610 PE=3 SV=1 - [A0A0B2S880_GLYSO]	3	0
A0A0B2QVE6	Beta-glucosidase 44 OS=Glycine soja GN=glysoja_033611 PE=3 SV=1 - [A0A0B2QVE6_GLYSO]	3	0
A0A0R0EDR0	Uncharacterized protein OS=Glycine max GN=GLYMA_20G196600 PE=4 SV=1 - [A0A0R0EDR0_SOYBN]	3	0
P01064	Bowman-Birk type proteinase inhibitor D-II OS=Glycine max PE=1 SV=2 - [IBBD2_SOYBN]	3	0
Q9XER5	Seed maturation protein PM22 OS=Glycine max GN=PM22 PE=2 SV=1 - [Q9XER5_SOYBN]	3	0
A0A0B2SSB2	26.5 kDa heat shock protein, mitochondrial OS=Glycine soja GN=glysoja_029181 PE=3 SV=1 - [A0A0B2SSB2_GLYSO]	3	0
I1KTM3	Uncharacterized protein OS=Glycine max GN=GLYMA_08G154900 PE=3 SV=1 - [I1KTM3_SOYBN]	3	0
K7K4G2	Uncharacterized protein OS=Glycine max GN=GLYMA_01G177000 PE=4 SV=1 - [K7K4G2_SOYBN]	3	0
A0A0B2R0Z0	1-Cys peroxiredoxin OS=Glycine soja GN=glysoja_018783 PE=4 SV=1 - [A0A0B2R0Z0_GLYSO]	3	0
A0A0B2PJR1	31 kDa ribonucleoprotein, chloroplastic OS=Glycine soja GN=glysoja_039843 PE=4 SV=1 - [A0A0B2PJR1_GLYSO]	3	0
A0A0B2Q0G3	Outer plastidial membrane protein porin OS=Glycine soja GN=glysoja_016419 PE=4 SV=1 - [A0A0B2Q0G3_GLYSO]	3	0

A1KR24	Dehydrin OS=Glycine max GN=LEA-2-D11 PE=3 SV=1 - [A1KR24_SOYBN]	3	0
Q01417	18 kDa seed maturation protein OS=Glycine max GN=GMPM1 PE=2 SV=1 - [PM1_SOYBN]	3	0
A0A0B2S9A1	Basic 7S globulin 2 OS=Glycine soja GN=glysoja_001854 PE=3 SV=1 - [A0A0B2S9A1_GLYSO]	3	0
K7MGG1	Uncharacterized protein OS=Glycine max GN=GLYMA_16G100400 PE=3 SV=1 - [K7MGG1_SOYBN]	2	0
I1JL98	Uncharacterized protein OS=Glycine max GN=GLYMA_03G052200 PE=4 SV=1 - [I1JL98_SOYBN]	2	0
K7MSK8	Uncharacterized protein OS=Glycine max GN=GLYMA_18G159800 PE=4 SV=1 - [K7MSK8_SOYBN]	2	0
O22120	Alpha subunit of beta conglycinin (Fragment) OS=Glycine max PE=2 SV=2 - [O22120_SOYBN]	2	0
A0A0B2RV43	Mitochondrial outer membrane protein porin 2 OS=Glycine soja GN=glysoja_003421 PE=4 SV=1 - [A0A0B2RV43_GLYSO]	2	0
C6TB95	Uncharacterized protein OS=Glycine max GN=GLYMA_01G218900 PE=2 SV=1 - [C6TB95_SOYBN]	2	0
I1LM67	Uncharacterized protein OS=Glycine max GN=GLYMA_13G153000 PE=4 SV=1 - [I1LM67_SOYBN]	2	0
A0A0B2P4D2	Glycinin G3 OS=Glycine soja GN=glysoja_002527 PE=4 SV=1 - [A0A0B2P4D2_GLYSO]	2	0
A0A0B2QLB8	Peroxisomal fatty acid beta-oxidation multifunctional protein AIM1 OS=Glycine soja GN=glysoja_018990 PE=3 SV=1 - [A0A0B2QLB8_GLYSO]	2	0
I1JYI8	Uncharacterized protein OS=Glycine max GN=GLYMA_04G229000 PE=3 SV=1 - [I1JYI8_SOYBN]	2	0
I1ND90	Carboxypeptidase OS=Glycine max GN=GLYMA_20G016800 PE=3 SV=2 - [I1ND90_SOYBN]	2	0
C6T828	Putative uncharacterized protein OS=Glycine max PE=2 SV=1 - [C6T828_SOYBN]	2	0
K7KFX8	Uncharacterized protein OS=Glycine max GN=GLYMA_03G190100 PE=4 SV=1 - [K7KFX8_SOYBN]	2	0
I1MFH9	Lactoylglutathione lyase OS=Glycine max GN=GLYMA_15G108400 PE=3 SV=1 - [I1MFH9_SOYBN]	2	0
K7L817	40S ribosomal protein S12 OS=Glycine max GN=GLYMA_08G217700 PE=3 SV=1 - [K7L817_SOYBN]	2	0
I1K6M2	Uncharacterized protein OS=Glycine max GN=GLYMA_05G204800 PE=4 SV=1 - [I1K6M2_SOYBN]	2	0
A0A0B2QKF8	Glucose and ribitol dehydrogenase OS=Glycine soja GN=glysoja_023775 PE=4 SV=1 - [A0A0B2QKF8_GLYSO]	2	0
Q9SWB6	Probable bifunctional TENA-E protein OS=Glycine max GN=TENA_E PE=2 SV=1 - [TENAE_SOYBN]	2	0
A0A0R0F0P9	Uncharacterized protein OS=Glycine max	2	0

	GN=GLYMA_18G166400 PE=4 SV=1 - [A0A0R0F0P9_SOYBN]		
C6T529	Ribulose bisphosphate carboxylase small chain OS=Glycine max PE=2 SV=1 - [C6T529_SOYBN]	2	0
A0A0R0F0E0	Uncharacterized protein OS=Glycine max GN=GLYMA_18G153900 PE=4 SV=1 - [A0A0R0F0E0_SOYBN]	2	0
A0A0B2PNU6	Heat shock protein 83 OS=Glycine soja GN=glysoja_029069 PE=3 SV=1 - [A0A0B2PNU6_GLYSO]	0	2
A0A0R4J3I5	Uncharacterized protein OS=Glycine max GN=GLYMA_06G137100 PE=3 SV=1 - [A0A0R4J3I5_SOYBN]	0	2
I1KSR7	Uncharacterized protein OS=Glycine max GN=GLYMA_08G127600 PE=4 SV=1 - [I1KSR7_SOYBN]	0	2
Q852U4	Glycinin A1bB2-784 OS=Glycine max PE=2 SV=1 - [Q852U4_SOYBN]	0	2
I1MU68	Uncharacterized protein OS=Glycine max GN=GLYMA_17G112500 PE=4 SV=2 - [I1MU68_SOYBN]	0	2
C6T8J1	Uncharacterized protein OS=Glycine max GN=GLYMA_13G098500 PE=2 SV=1 - [C6T8J1_SOYBN]	0	2
A0A0B2NZI1	T-complex protein 1 subunit theta OS=Glycine soja GN=glysoja_000342 PE=3 SV=1 - [A0A0B2NZI1_GLYSO]	0	2
A0A0B2NY39	S-formylglutathione hydrolase OS=Glycine soja GN=glysoja_047614 PE=3 SV=1 - [A0A0B2NY39_GLYSO]	0	2
K7K9D0	Uncharacterized protein OS=Glycine max GN=GLYMA_02G184600 PE=4 SV=1 - [K7K9D0_SOYBN]	0	2
C6TN99	Putative uncharacterized protein OS=Glycine max PE=2 SV=1 - [C6TN99_SOYBN]	0	2
C6TCN5	Ferritin OS=Glycine max GN=GLYMA_18G205800 PE=2 SV=1 - [C6TCN5_SOYBN]	0	2
C6TFC7	Putative uncharacterized protein OS=Glycine max PE=2 SV=1 - [C6TFC7_SOYBN]	0	2
Q70EM0	Dehydrin OS=Glycine max GN=lea-D-11 PE=3 SV=1 - [Q70EM0_SOYBN]	0	2
A0A0B2SQI5	Uncharacterized protein OS=Glycine soja GN=glysoja_015000 PE=4 SV=1 - [A0A0B2SQI5_GLYSO]	0	2
I1MNR1	Uncharacterized protein OS=Glycine max GN=GLYMA_16G150000 PE=4 SV=1 - [I1MNR1_SOYBN]	0	2
I1KIE4	Uncharacterized protein OS=Glycine max GN=GLYMA_07G073800 PE=3 SV=1 - [I1KIE4_SOYBN]	0	2
A0A0R0H1C4	Uncharacterized protein OS=Glycine max GN=GLYMA_12G031900 PE=4 SV=1 - [A0A0R0H1C4_SOYBN]	0	2
C6SX26	Putative uncharacterized protein OS=Glycine max PE=2 SV=1 - [C6SX26_SOYBN]	0	3

A0A0R0HKE3	Uncharacterized protein OS=Glycine max GN=GLYMA_11G214800 PE=4 SV=1 - [A0A0R0HKE3_SOYBN]	0	3
I1KYW3	Uncharacterized protein OS=Glycine max GN=GLYMA_08G341000 PE=4 SV=1 - [I1KYW3_SOYBN]	0	3
I1KS58	Uncharacterized protein OS=Glycine max GN=GLYMA_08G108000 PE=4 SV=1 - [I1KS58_SOYBN]	0	3
O22121	Beta subunit of beta conglycinin (Fragment) OS=Glycine max PE=2 SV=2 - [O22121_SOYBN]	0	3
K7K8E5	Uncharacterized protein OS=Glycine max GN=GLYMA_02G147200 PE=4 SV=1 - [K7K8E5_SOYBN]	0	3
I1MAE6	Uncharacterized protein OS=Glycine max GN=GLYMA_14G121200 PE=3 SV=1 - [I1MAE6_SOYBN]	0	3

Table S13. The list of DEPs in the sample 117/113 (A3525/Zhonghuang13).

ID	Description	Sig. (Diff.117/113) times	
		up	down
I1N520	Uncharacterized protein OS=Glycine max GN=GLYMA_18G285800 PE=4 SV=1 - [I1N520_SOYBN]	3	0
A0A0B2PMR9	Em-like protein GEA6 OS=Glycine soja GN=glysoja_045231 PE=4 SV=1 - [A0A0B2PMR9_GLYSO]	3	0
Q42447	Maturation protein OS=Glycine max GN=MAT1 PE=2 SV=1 - [Q42447_SOYBN]	3	0
I1KQW4	Uncharacterized protein OS=Glycine max PE=3 SV=2 - [I1KQW4_SOYBN]	3	0
C6T3R4	60S ribosomal protein L27 OS=Glycine max GN=GLYMA_05G132800 PE=2 SV=1 - [C6T3R4_SOYBN]	3	0
I1LM67	Uncharacterized protein OS=Glycine max GN=GLYMA_13G153000 PE=4 SV=1 - [I1LM67_SOYBN]	3	0
A0A0R0HM17	Uncharacterized protein OS=Glycine max GN=GLYMA_11G079600 PE=3 SV=1 - [A0A0R0HM17_SOYBN]	3	0
I1N334	Uncharacterized protein OS=Glycine max GN=GLYMA_18G208000 PE=4 SV=2 - [I1N334_SOYBN]	3	0
K7N3S3	60S ribosomal protein L36 OS=Glycine max GN=GLYMA_20G160400 PE=3 SV=1 - [K7N3S3_SOYBN]	3	0
C6T3U6	Ribosomal protein L19 OS=Glycine max GN=GLYMA_19G208200 PE=2 SV=1 - [C6T3U6_SOYBN]	3	0
I1KRI7	60S ribosomal protein L27 OS=Glycine max GN=GLYMA_08G087200 PE=3 SV=1 - [I1KRI7_SOYBN]	3	0
A0A0B2RZZ6	Polyadenylate-binding protein-interacting protein 2 OS=Glycine soja GN=glysoja_004220 PE=4 SV=1 - [A0A0B2RZZ6_GLYSO]	2	0
I1LVC1	Uncharacterized protein OS=Glycine max GN=GLYMA_12G235800 PE=4 SV=1 - [I1LVC1_SOYBN]	2	0

I1KN68	Glycosyltransferase OS=Glycine max GN=GmSg-1 PE=3 SV=1 - [I1KN68_SOYBN]	2	0
C6T4I7	Ribosomal protein L19 OS=Glycine max GN=GLYMA_19G213200 PE=2 SV=1 - [C6T4I7_SOYBN]	2	0
A0A0R0GV71	Uncharacterized protein OS=Glycine max GN=GLYMA_13G151600 PE=4 SV=1 - [A0A0R0GV71_SOYBN]	2	0
C6TKM8	Putative uncharacterized protein OS=Glycine max PE=2 SV=1 - [C6TKM8_SOYBN]	2	0
A0A0B2R3J3	Ribosomal protein L19 OS=Glycine soja GN=glysoja_038842 PE=3 SV=1 - [A0A0B2R3J3_GLYSO]	2	0
I1J8Y8	Uncharacterized protein OS=Glycine max GN=GLYMA_01G178200 PE=3 SV=1 - [I1J8Y8_SOYBN]	2	0
I1MYX8	Alpha-SNAP protein OS=Glycine max GN=GLYMA_18G022500 PE=4 SV=1 - [I1MYX8_SOYBN]	2	0
I1MP01	Glycosyltransferase OS=Glycine max GN=GLYMA_16G158100 PE=3 SV=1 - [I1MP01_SOYBN]	2	0
A0A0B2QYA8	Dynein light chain 2, cytoplasmic OS=Glycine soja GN=glysoja_019779 PE=4 SV=1 - [A0A0B2QYA8_GLYSO]	2	0
A0A0B2RHJ1	40S ribosomal protein S24 OS=Glycine soja GN=glysoja_035550 PE=3 SV=1 - [A0A0B2RHJ1_GLYSO]	2	0
A0A0B2S8C8	Uncharacterized protein OS=Glycine soja GN=glysoja_007471 PE=4 SV=1 - [A0A0B2S8C8_GLYSO]	2	0
A0A0B2NSC6	40S ribosomal protein S5 OS=Glycine soja GN=glysoja_034227 PE=3 SV=1 - [A0A0B2NSC6_GLYSO]	2	0
I1LDC2	Uncharacterized protein OS=Glycine max GN=GLYMA_10G220800 PE=4 SV=1 - [I1LDC2_SOYBN]	2	0
I1NAH4	Uncharacterized protein OS=Glycine max GN=GLYMA_19G189600 PE=4 SV=1 - [I1NAH4_SOYBN]	2	0
A0A0B2QKF8	Glucose and ribitol dehydrogenase OS=Glycine soja GN=glysoja_023775 PE=4 SV=1 - [A0A0B2QKF8_GLYSO]	2	0
K7N4A7	Uncharacterized protein OS=Glycine max PE=3 SV=1 - [K7N4A7_SOYBN]	2	0
A0A0R0F0E0	Uncharacterized protein OS=Glycine max GN=GLYMA_18G153900 PE=4 SV=1 - [A0A0R0F0E0_SOYBN]	2	0
A0A0B2RQ67	Argininosuccinate synthase, chloroplastic OS=Glycine soja GN=glysoja_016736 PE=3 SV=1 - [A0A0B2RQ67_GLYSO]	2	0
C6SX26	Putative uncharacterized protein OS=Glycine max PE=2 SV=1 - [C6SX26_SOYBN]	0	2
A0A0R0IIZ3	Uncharacterized protein OS=Glycine max GN=GLYMA_09G178100 PE=3 SV=1 - [A0A0R0IIZ3_SOYBN]	0	2
A0A0B2SQP4	Clustered mitochondria protein homolog OS=Glycine soja GN=glysoja_031237 PE=3 SV=1 - [A0A0B2SQP4_GLYSO]	0	2

A0A0B2RCV2	Puromycin-sensitive aminopeptidase OS=Glycine soja GN=glysoja_016324 PE=4 SV=1 - [A0A0B2RCV2_GLYSO]	0	2
B1Q2X5	Protein disulfide-isomerase OS=Glycine max GN=PDIL-2 PE=2 SV=1 - [B1Q2X5_SOYBN]	0	2
A0A0R0J9Y0	Uncharacterized protein OS=Glycine max GN=GLYMA_07G195900 PE=4 SV=1 - [A0A0R0J9Y0_SOYBN]	0	2
I1JAQ7	Uncharacterized protein OS=Glycine max GN=GLYMA_01G235600 PE=4 SV=2 - [I1JAQ7_SOYBN]	0	2
I1JUY7	ATP-dependent 6-phosphofructokinase OS=Glycine max GN=PFK PE=3 SV=1 - [I1JUY7_SOYBN]	0	2
C6TAR9	Putative uncharacterized protein OS=Glycine max PE=2 SV=1 - [C6TAR9_SOYBN]	0	2
Q7M1R5	Superoxide dismutase [Cu-Zn] OS=Glycine max GN=SOD1 PE=3 SV=1 - [SODC_SOYBN]	0	2
C6ZJY8	Serine hydroxymethyltransferase OS=Glycine max GN=GLYMA_08G274400 PE=2 SV=1 - [C6ZJY8_SOYBN]	0	2
A0A0R0FPQ1	Uncharacterized protein OS=Glycine max GN=GLYMA_16G118300 PE=4 SV=1 - [A0A0R0FPQ1_SOYBN]	0	2
Q70EM0	Dehydrin OS=Glycine max GN=lea-D-11 PE=3 SV=1 - [Q70EM0_SOYBN]	0	2
A0A0B2SQI5	Uncharacterized protein OS=Glycine soja GN=glysoja_015000 PE=4 SV=1 - [A0A0B2SQI5_GLYSO]	0	2
A0A0R0JWC4	Uncharacterized protein OS=Glycine max GN=GLYMA_06G324000 PE=4 SV=1 - [A0A0R0JWC4_SOYBN]	0	2
A0A0B2Q1U6	Uncharacterized protein OS=Glycine soja GN=glysoja_025011 PE=4 SV=1 - [A0A0B2Q1U6_GLYSO]	0	2
I1MTN1	Uncharacterized protein OS=Glycine max GN=GLYMA_17G094800 PE=4 SV=1 - [I1MTN1_SOYBN]	0	2
A0A0R0H1C4	Uncharacterized protein OS=Glycine max GN=GLYMA_12G031900 PE=4 SV=1 - [A0A0R0H1C4_SOYBN]	0	2
A0A0R0ENW6	Uncharacterized protein OS=Glycine max GN=GLYMA_20G210100 PE=3 SV=1 - [A0A0R0ENW6_SOYBN]	0	3
F7J077	Beta-conglycinin beta subunit OS=Glycine max GN=CG-beta-2 PE=4 SV=1 - [F7J077_SOYBN]	0	3
I1M395	Uncharacterized protein OS=Glycine max GN=GLYMA_13G278000 PE=3 SV=1 - [I1M395_SOYBN]	0	3
A0A0B2RJR9	Urease OS=Glycine soja GN=glysoja_036799 PE=3 SV=1 - [A0A0B2RJR9_GLYSO]	0	3
Q9XET1	Seed maturation protein PM31 OS=Glycine max GN=PM31 PE=2	0	3

	SV=1 - [Q9XET1_SOYBN]		
A0A0B2QAS8	Glycine-rich RNA-binding protein 2, mitochondrial OS=Glycine soja GN=glysoja_006768 PE=4 SV=1 - [A0A0B2QAS8_GLYSO]	0	3
P45458	Malate synthase, glyoxysomal (Fragment) OS=Glycine max PE=2 SV=1 - [MASY_SOYBN]	0	3
I1JL08	Non-specific lipid-transfer protein OS=Glycine max GN=GLYMA_03G040000 PE=3 SV=1 - [I1JL08_SOYBN]	0	3
I1JLC8	Protein SLE2 OS=Glycine max GN=SLE2 PE=2 SV=1 - [SLE2_SOYBN]	0	3
Q04672	Sucrose-binding protein OS=Glycine max GN=SBP PE=1 SV=1 - [SBP_SOYBN]	0	3
I1K3Q1	Cysteine proteinase inhibitor OS=Glycine max GN=GLYMA_05G149800 PE=3 SV=1 - [I1K3Q1_SOYBN]	0	3
C6T1V2	Uncharacterized protein OS=Glycine max GN=GLYMA_14G063700 PE=2 SV=1 - [C6T1V2_SOYBN]	0	3
A0A0R0GA14	Uncharacterized protein OS=Glycine max GN=GLYMA_14G063800 PE=3 SV=1 - [A0A0R0GA14_SOYBN]	0	3
P13917	Basic 7S globulin OS=Glycine max GN=BG PE=1 SV=2 - [7SB1_SOYBN]	0	3
C6TAX7	Uncharacterized protein OS=Glycine max GN=GLYMA_04G009400 PE=2 SV=1 - [C6TAX7_SOYBN]	0	3

Table S14. The list of DEPs in the sample 119/113 (FG72-JACK/Zhonghuang13).

ID	Description	Sig. (Diff.119/113) times	
		up	down
C6SX26	Putative uncharacterized protein OS=Glycine max PE=2 SV=1 - [C6SX26_SOYBN]	3	0
Q42447	Maturation protein OS=Glycine max GN=MAT1 PE=2 SV=1 - [Q42447_SOYBN]	3	0
C6T3R4	60S ribosomal protein L27 OS=Glycine max GN=GLYMA_05G132800 PE=2 SV=1 - [C6T3R4_SOYBN]	3	0
A0A0R0HKE3	Uncharacterized protein OS=Glycine max GN=GLYMA_11G214800 PE=4 SV=1 - [A0A0R0HKE3_SOYBN]	3	0
A0A0R0HM17	Uncharacterized protein OS=Glycine max GN=GLYMA_11G079600 PE=3 SV=1 - [A0A0R0HM17_SOYBN]	3	0
C6T571	Histone H2B OS=Glycine max PE=2 SV=1 - [C6T571_SOYBN]	3	0
O22121	Beta subunit of beta conglycinin (Fragment) OS=Glycine max PE=2 SV=2 - [O22121_SOYBN]	3	0
K7K8E5	Uncharacterized protein OS=Glycine max GN=GLYMA_02G147200 PE=4 SV=1 - [K7K8E5_SOYBN]	3	0

I1MAE6	Uncharacterized protein OS=Glycine max GN=GLYMA_14G121200 PE=3 SV=1 - [I1MAE6_SOYBN]	3	0
K7N3S3	60S ribosomal protein L36 OS=Glycine max GN=GLYMA_20G160400 PE=3 SV=1 - [K7N3S3_SOYBN]	3	0
K7K9D0	Uncharacterized protein OS=Glycine max GN=GLYMA_02G184600 PE=4 SV=1 - [K7K9D0_SOYBN]	3	0
A0A0R4J4L3	Annexin OS=Glycine max GN=GLYMA_11G153800 PE=3 SV=1 - [A0A0R4J4L3_SOYBN]	3	0
C6T3U6	Ribosomal protein L19 OS=Glycine max GN=GLYMA_19G208200 PE=2 SV=1 - [C6T3U6_SOYBN]	3	0
I1KIE4	Uncharacterized protein OS=Glycine max GN=GLYMA_07G073800 PE=3 SV=1 - [I1KIE4_SOYBN]	3	0
C6TF48	Putative uncharacterized protein OS=Glycine max PE=2 SV=1 - [C6TF48_SOYBN]	2	0
A0A0B2PMR9	Em-like protein GEA6 OS=Glycine soja GN=glysoja_045231 PE=4 SV=1 - [A0A0B2PMR9_GLYSO]	2	0
Q9FQ95	In2-1 protein OS=Glycine max GN=GSTL5 PE=2 SV=1 - [Q9FQ95_SOYBN]	2	0
C6T4I7	Ribosomal protein L19 OS=Glycine max GN=GLYMA_19G213200 PE=2 SV=1 - [C6T4I7_SOYBN]	2	0
I1KSR7	Uncharacterized protein OS=Glycine max GN=GLYMA_08G127600 PE=4 SV=1 - [I1KSR7_SOYBN]	2	0
C6TKM8	Putative uncharacterized protein OS=Glycine max PE=2 SV=1 - [C6TKM8_SOYBN]	2	0
I1KS58	Uncharacterized protein OS=Glycine max GN=GLYMA_08G108000 PE=4 SV=1 - [I1KS58_SOYBN]	2	0
I1KGH6	Uncharacterized protein OS=Glycine max GN=GLYMA_07G014400 PE=3 SV=1 - [I1KGH6_SOYBN]	2	0
I1MU68	Uncharacterized protein OS=Glycine max GN=GLYMA_17G112500 PE=4 SV=2 - [I1MU68_SOYBN]	2	0
C6T8J1	Uncharacterized protein OS=Glycine max GN=GLYMA_13G098500 PE=2 SV=1 - [C6T8J1_SOYBN]	2	0
I1MYX8	Alpha-SNAP protein OS=Glycine max GN=GLYMA_18G022500 PE=4 SV=1 - [I1MYX8_SOYBN]	2	0
A0A0B2P1M7	U-box domain-containing protein 35 (Fragment) OS=Glycine soja GN=glysoja_009585 PE=4 SV=1 - [A0A0B2P1M7_GLYSO]	2	0
I1N5S0	Uncharacterized protein OS=Glycine max GN=GLYMA_19G009100 PE=3 SV=1 - [I1N5S0_SOYBN]	2	0
A0A0B2QUL3	Tropinone reductase like OS=Glycine soja GN=glysoja_010147 PE=4 SV=1 - [A0A0B2QUL3_GLYSO]	2	0
A0A0B2S8C8	Uncharacterized protein OS=Glycine soja GN=glysoja_007471 PE=4 SV=1 - [A0A0B2S8C8_GLYSO]	2	0
A0A0B2Q5P0	Uncharacterized protein OS=Glycine soja GN=glysoja_011760 PE=4 SV=1 - [A0A0B2Q5P0_GLYSO]	2	0

C6TN99	Putative uncharacterized protein OS=Glycine max PE=2 SV=1 - [C6TN99_SOYBN]	2	0
I1NAH4	Uncharacterized protein OS=Glycine max GN=GLYMA_19G189600 PE=4 SV=1 - [I1NAH4_SOYBN]	2	0
A0A0B2QKF8	Glucose and ribitol dehydrogenase OS=Glycine soja GN=glysoja_023775 PE=4 SV=1 - [A0A0B2QKF8_GLYSO]	2	0
P62163	Calmodulin-2 OS=Glycine max GN=CAM-2 PE=1 SV=2 - [CALM2_SOYBN]	2	0
I1MNR1	Uncharacterized protein OS=Glycine max GN=GLYMA_16G150000 PE=4 SV=1 - [I1MNR1_SOYBN]	2	0
P50346	60S acidic ribosomal protein P0 OS=Glycine max PE=2 SV=1 - [RLA0_SOYBN]	2	0
K7N4A7	Uncharacterized protein OS=Glycine max PE=3 SV=1 - [K7N4A7_SOYBN]	2	0
A0A0B2RFQ6	Oxysterol-binding protein-related protein 3B OS=Glycine soja GN=glysoja_028925 PE=3 SV=1 - [A0A0B2RFQ6_GLYSO]	0	2
K7MGG1	Uncharacterized protein OS=Glycine max GN=GLYMA_16G100400 PE=3 SV=1 - [K7MGG1_SOYBN]	0	2
C6SWU2	Putative uncharacterized protein OS=Glycine max PE=2 SV=1 - [C6SWU2_SOYBN]	0	2
C6TFT6	4-hydroxy-4-methyl-2-oxoglutarate aldolase OS=Glycine max PE=2 SV=1 - [C6TFT6_SOYBN]	0	2
A0A0B2PFY3	60S acidic ribosomal protein P3 OS=Glycine soja GN=glysoja_010887 PE=4 SV=1 - [A0A0B2PFY3_GLYSO]	0	2
I1KQW4	Uncharacterized protein OS=Glycine max PE=3 SV=2 - [I1KQW4_SOYBN]	0	2
I1LUY8	Uncharacterized protein OS=Glycine max GN=GLYMA_12G222400 PE=4 SV=1 - [I1LUY8_SOYBN]	0	2
C6TNT7	Putative uncharacterized protein (Fragment) OS=Glycine max PE=2 SV=1 - [C6TNT7_SOYBN]	0	2
I1J7M1	Non-specific lipid-transfer protein OS=Glycine max GN=GLYMA_01G130200 PE=3 SV=2 - [I1J7M1_SOYBN]	0	2
O22120	Alpha subunit of beta conglycinin (Fragment) OS=Glycine max PE=2 SV=2 - [O22120_SOYBN]	0	2
I1JXM1	HVA22-like protein OS=Glycine max GN=GLYMA_04G197600 PE=3 SV=1 - [I1JXM1_SOYBN]	0	2
A0A0B2S880	5-methyltetrahydropteroyltriglutamate--homocysteine methyltransferase OS=Glycine soja GN=glysoja_048610 PE=3 SV=1 - [A0A0B2S880_GLYSO]	0	2
I1JAQ7	Uncharacterized protein OS=Glycine max GN=GLYMA_01G235600 PE=4 SV=2 - [I1JAQ7_SOYBN]	0	2
A0A0B2RJR9	Urease OS=Glycine soja GN=glysoja_036799 PE=3 SV=1 - [A0A0B2RJR9_GLYSO]	0	2
I1N5I8	Oleosin OS=Glycine max GN=GLYMA_19G004800 PE=3 SV=1	0	2

	- [I1N5I8_SOYBN]		
I1JBI5	Uncharacterized protein OS=Glycine max GN=GLYMA_02G014800 PE=3 SV=1 - [I1JBI5_SOYBN]	0	2
I1KTM3	Uncharacterized protein OS=Glycine max GN=GLYMA_08G154900 PE=3 SV=1 - [I1KTM3_SOYBN]	0	2
I1ND90	Carboxypeptidase OS=Glycine max GN=GLYMA_20G016800 PE=3 SV=2 - [I1ND90_SOYBN]	0	2
Q7M1R5	Superoxide dismutase [Cu-Zn] OS=Glycine max GN=SOD1 PE=3 SV=1 - [SODC_SOYBN]	0	2
G3E7M9	Annexin OS=Glycine max GN=GLYMA_13G088700 PE=2 SV=1 - [G3E7M9_SOYBN]	0	2
C6T9W4	Putative Fe(II)-and 2-oxoglutaratedependent dioxygenase OS=Glycine max GN=F6'H1 PE=2 SV=1 - [C6T9W4_SOYBN]	0	2
A0A0R0JL19	Uncharacterized protein OS=Glycine max GN=GLYMA_06G122600 PE=3 SV=1 - [A0A0R0JL19_SOYBN]	0	2
C6ZJY8	Serine hydroxymethyltransferase OS=Glycine max GN=GLYMA_08G274400 PE=2 SV=1 - [C6ZJY8_SOYBN]	0	2
K7L817	40S ribosomal protein S12 OS=Glycine max GN=GLYMA_08G217700 PE=3 SV=1 - [K7L817_SOYBN]	0	2
I1KQE3	Uncharacterized protein OS=Glycine max GN=GLYMA_08G048200 PE=3 SV=1 - [I1KQE3_SOYBN]	0	2
I1KVR2	Uncharacterized protein OS=Glycine max GN=GLYMA_08G222100 PE=3 SV=1 - [I1KVR2_SOYBN]	0	2
A0A0B2S970	Aminomethyltransferase OS=Glycine soja GN=glysoja_015246 PE=3 SV=1 - [A0A0B2S970_GLYSO]	0	2
I1K6M2	Uncharacterized protein OS=Glycine max GN=GLYMA_05G204800 PE=4 SV=1 - [I1K6M2_SOYBN]	0	2
A0A0R0FPQ1	Uncharacterized protein OS=Glycine max GN=GLYMA_16G118300 PE=4 SV=1 - [A0A0R0FPQ1_SOYBN]	0	2
Q70EM0	Dehydrin OS=Glycine max GN=lea-D-11 PE=3 SV=1 - [Q70EM0_SOYBN]	0	2
Q01417	18 kDa seed maturation protein OS=Glycine max GN=GMPM1 PE=2 SV=1 - [PM1_SOYBN]	0	2
A0A0R0JWC4	Uncharacterized protein OS=Glycine max GN=GLYMA_06G324000 PE=4 SV=1 - [A0A0R0JWC4_SOYBN]	0	2
C6T529	Ribulose bisphosphate carboxylase small chain OS=Glycine max PE=2 SV=1 - [C6T529_SOYBN]	0	2
Q948P5	Ferritin-4, chloroplastic OS=Glycine max PE=1 SV=2 - [FRI4_SOYBN]	0	2
I1MTN1	Uncharacterized protein OS=Glycine max GN=GLYMA_17G094800 PE=4 SV=1 - [I1MTN1_SOYBN]	0	2

I1LDP2	Uncharacterized protein OS=Glycine max GN=GLYMA_10G233300 PE=4 SV=1 - [I1LDP2_SOYBN]	0	2
K7MSK8	Uncharacterized protein OS=Glycine max GN=GLYMA_18G159800 PE=4 SV=1 - [K7MSK8_SOYBN]	0	3
A0A0R0IIZ3	Uncharacterized protein OS=Glycine max GN=GLYMA_09G178100 PE=3 SV=1 - [A0A0R0IIZ3_SOYBN]	0	3
F7J077	Beta-conglycinin beta subunit OS=Glycine max GN=CG-beta-2 PE=4 SV=1 - [F7J077_SOYBN]	0	3
Q01527	Maturation protein OS=Glycine max GN=gGmpm9 PE=2 SV=1 - [Q01527_SOYBN]	0	3
I1M395	Uncharacterized protein OS=Glycine max GN=GLYMA_13G278000 PE=3 SV=1 - [I1M395_SOYBN]	0	3
A0A0R0L186	Uncharacterized protein OS=Glycine max GN=GLYMA_02G252800 PE=3 SV=1 - [A0A0R0L186_SOYBN]	0	3
A0A0B2QVE6	Beta-glucosidase 44 OS=Glycine soja GN=glysoja_033611 PE=3 SV=1 - [A0A0B2QVE6_GLYSO]	0	3
A0A0R0K7M8	Uncharacterized protein OS=Glycine max GN=GLYMA_04G122900 PE=4 SV=1 - [A0A0R0K7M8_SOYBN]	0	3
A0A0R0EDR0	Uncharacterized protein OS=Glycine max GN=GLYMA_20G196600 PE=4 SV=1 - [A0A0R0EDR0_SOYBN]	0	3
P01064	Bowman-Birk type proteinase inhibitor D-II OS=Glycine max PE=1 SV=2 - [IBBD2_SOYBN]	0	3
C6TAR9	Putative uncharacterized protein OS=Glycine max PE=2 SV=1 - [C6TAR9_SOYBN]	0	3
Q9XER5	Seed maturation protein PM22 OS=Glycine max GN=PM22 PE=2 SV=1 - [Q9XER5_SOYBN]	0	3
A0A0B2P4D2	Glycinin G3 OS=Glycine soja GN=glysoja_002527 PE=4 SV=1 - [A0A0B2P4D2_GLYSO]	0	3
A0A0B2PJM6	Seed biotin-containing protein SBP65 OS=Glycine soja GN=glysoja_033843 PE=4 SV=1 - [A0A0B2PJM6_GLYSO]	0	3
Q9XET1	Seed maturation protein PM31 OS=Glycine max GN=PM31 PE=2 SV=1 - [Q9XET1_SOYBN]	0	3
K7K4G2	Uncharacterized protein OS=Glycine max GN=GLYMA_01G177000 PE=4 SV=1 - [K7K4G2_SOYBN]	0	3
A0A0B2QAS8	Glycine-rich RNA-binding protein 2, mitochondrial OS=Glycine soja GN=glysoja_006768 PE=4 SV=1 - [A0A0B2QAS8_GLYSO]	0	3
P45458	Malate synthase, glyoxysomal (Fragment) OS=Glycine max PE=2 SV=1 - [MASY_SOYBN]	0	3
I1JL08	Non-specific lipid-transfer protein OS=Glycine max GN=GLYMA_03G040000 PE=3 SV=1 - [I1JL08_SOYBN]	0	3
I1JLC8	Protein SLE2 OS=Glycine max GN=SLE2 PE=2 SV=1 -	0	3

	[SLE2_SOYBN]		
Q04672	Sucrose-binding protein OS=Glycine max GN=SBP PE=1 SV=1 - [SBP_SOYBN]	0	3
I1K3Q1	Cysteine proteinase inhibitor OS=Glycine max GN=GLYMA_05G149800 PE=3 SV=1 - [I1K3Q1_SOYBN]	0	3
A0A0B2R0Z0	1-Cys peroxiredoxin OS=Glycine soja GN=glysoja_018783 PE=4 SV=1 - [A0A0B2R0Z0_GLYSO]	0	3
C6T1V2	Uncharacterized protein OS=Glycine max GN=GLYMA_14G063700 PE=2 SV=1 - [C6T1V2_SOYBN]	0	3
A0A0B2Q0G3	Outer plastidial membrane protein porin OS=Glycine soja GN=glysoja_016419 PE=4 SV=1 - [A0A0B2Q0G3_GLYSO]	0	3
A1KR24	Dehydrin OS=Glycine max GN=LEA-2-D11 PE=3 SV=1 - [A1KR24_SOYBN]	0	3
A0A0B2S9A1	Basic 7S globulin 2 OS=Glycine soja GN=glysoja_001854 PE=3 SV=1 - [A0A0B2S9A1_GLYSO]	0	3
A0A0R0GA14	Uncharacterized protein OS=Glycine max GN=GLYMA_14G063800 PE=3 SV=1 - [A0A0R0GA14_SOYBN]	0	3
P13917	Basic 7S globulin OS=Glycine max GN=BG PE=1 SV=2 - [7SB1_SOYBN]	0	3
C6TAX7	Uncharacterized protein OS=Glycine max GN=GLYMA_04G009400 PE=2 SV=1 - [C6TAX7_SOYBN]	0	3

Table S15. Primers for event-specific PCR detection.

**Soybean line**	**Primer**	**Sequence (5'-3')**	**Size (bp)**	**Chinese National Standards**
MON87705	MON87705-F	CGCCAAATCGTGAAGTTTCTCATCT	318	MOA-2122-4-2014
	MON87705-R	CAGTGATAACAACACCCTGAGTCT		
MON87708	MON87708-F	CCATCATACTCATTGCTGATCCA	233	MOA-2259-6-2015
	MON87708-R	AGCCAATCAATCTCAGAACTGTC		
FG72	FG72-F	TCGGGCTGCAGGGAATTAATGT	150	MOA-2259-8-2015
	FG72-R	TTTGGAGCAATAAACATGTGATAGC		
MON87701× MON89788	MON87701-MF	GCACGCTTAGTGTGTGTGTCAAAC	150	MOA-2259-7-2015
	MON87701-MR	GGATCCGTCGACCTGCAGTTAAC		
	MON89788-F	CTGCTCCACTCTTCCTTT	223	MOA-1485-6-2010
	MON89788-R	AGACTCTGTACCCTGACCT		
